# Complete Characterization of Incorrect Orthology Assignments in Best Match Graphs

**DOI:** 10.1007/s00285-021-01564-8

**Published:** 2021-02-19

**Authors:** David Schaller, Manuela Geiß, Peter F. Stadler, Marc Hellmuth

**Affiliations:** 1 Max-Planck-Institute for Mathematics in the Sciences, Inselstraße 22, D-04103 Leipzig, Germany; 2 Bioinformatics Group, Department of Computer Science, and Interdisciplinary Center of Bioinformatics, University of Leipzig, Härtelstraße 16-18, D-04107 Leipzig, Germany; 3 Software Competence Center Hagenberg GmbH, Softwarepark 21, A-4232 Hagenberg, Austria; 4 Bioinformatics Group, Department of Computer Science, Interdisciplinary Center of Bioinformatics, German Centre for Integrative Biodiversity Research (iDiv) Halle-Jena-Leipzig, Competence Center for Scalable Data Services and Solutions, and Leipzig Research Center for Civilization Diseases, Leipzig University, Härtelstraße 16-18, D-04107 Leipzig, Germany; 5 Inst. f. Theoretical Chemistry, University of Vienna, Währingerstraße 17, A-1090 Wien, Austria; 6 Facultad de Ciencias, Universidad National de Colombia, Bogotá, Colombia; 7 Santa Fe Institute, 1399 Hyde Park Rd., Santa Fe, NM 87501 USA; 8 Department of Mathematics, Faculty of Science, Stockholm University, SE 106 91 Stockholm, Sweden

**Keywords:** Orthology detection, Best matches, Unambiguous orthologs, Colored graphs, Cograph, Tree reconciliation, Polynomial-time algorithm, 92-08, 92D15, 68R01

## Abstract

Genome-scale orthology assignments are usually based on reciprocal best matches. In the absence of horizontal gene transfer (HGT), every pair of orthologs forms a reciprocal best match. Incorrect orthology assignments therefore are always false positives in the reciprocal best match graph. We consider duplication/loss scenarios and characterize unambiguous false-positive (*u-fp*) orthology assignments, that is, edges in the best match graphs (BMGs) that cannot correspond to orthologs for any gene tree that explains the BMG. Moreover, we provide a polynomial-time algorithm to identify all *u-fp* orthology assignments in a BMG. Simulations show that at least $75\%$ of all incorrect orthology assignments can be detected in this manner. All results rely only on the structure of the BMGs and *not* on any *a priori* knowledge about underlying gene or species trees.

## Introduction

Orthology is one of the key concepts in evolutionary biology: Two genes are orthologs if their last common ancestor was a speciation event Fitch (1970). Distinguishing orthologs from paralogs (originating from gene duplications) or xenologs (i.e., genes that have undergone horizontal gene transfer) is of considerable practical importance for functional genome annotation and thus for a wide array of methods in bioinformatics and computational biology that rely on gene annotation data. In particular, according to the “ortholog conjecture”, orthologous genes in different species are expected to have essentially the same biological and molecular functions, whereas paralogs and xenologs tend to have similar, but distinct functions. Albeit controversial Nehrt et al. (2011), Stamboulian et al. (2020), this assumption is widely made in the computational prediction of gene functions Nehrt et al. (2011), Gabaldón and Koonin (2013), Soria et al. (2014), Zallot et al. (2016). Moreover, the distinction of orthologs and paralogs is crucial in phylogenomics Delsuc et al. (2005). Most of the commonly used tools for large-scale orthology identification compute reciprocal best hits as a first step followed by some filtering and refinement steps to improve the results Tatusov et al. (2000), Roth et al. (2008), Lechner et al. (2011), Linard et al. (2011), Sonnhammer and Östlund (2015), Train et al. (2017), Huerta-Cepas et al. (2018), see also Nichio et al. (2017), Setubal and Stadler (2018), Galperin et al. (2019) for reviews and Altenhoff et al. (2016) for benchmarking results.

Orthology identification has also received increasing attention from a mathematical perspective starting from the concept of an *evolutionary scenario* comprising a gene tree *T* and a species tree *S* together with a *reconciliation map* $\mu$ from *T* to *S*. The map $\mu$ identifies the locations in the species tree at which evolutionary events, represented by the vertices of the gene tree, took place. *In this contribution, we consider exclusively duplication/loss scenarios, i.e., we explicitly exclude horizontal gene transfer.* Characterizations of reconciliation maps are given e.g. in Górecki and Tiuryn (2006), Vernot et al. (2008), Doyon et al. (2011), Rusin et al. (2014). While every gene tree can be reconciled with any species tree Guigó et al. (1996), Page and Charleston (1997), this is no longer true if event-labels are prescribed in the gene tree *T* Hernandez-Rosales et al. (2012), Lafond and El-Mabrouk (2014), Hellmuth (2017).

The orthology relation itself has been characterized as a cograph (i.e., graphs that do not contain induced paths $P_4$ on four vertices) by Hellmuth et al. (2013) based on earlier work by Böcker and Dress (1998). This line of research has led to the idea of editing reciprocal best hit data to conform to the required cograph structure Hellmuth et al. (2015). There are, however, two distinct sources of errors in an orthology assignment pipeline based on best matches:

(i) inaccuracies in the assignment of best matches from sequence similarity data Stadler et al. (2020), and
(ii) limits in the reconstruction of the “true” orthology relation from best match graphs Geiß et al. (2020b).

**Fig. 1:**
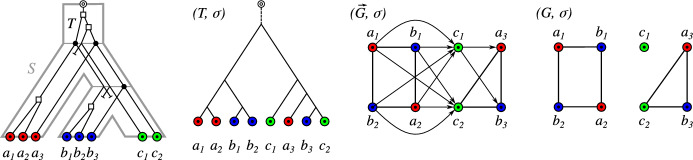
An evolutionary scenario (left) consists of a gene tree $(T,\sigma )$ (whose observable part is shown in the second panel) together with an embedding into a species tree *S*. The coloring $\sigma$ of the leaves of *T* represents the species in which the genes reside. Speciation vertices ($\newmoon$) of the gene tree coincide with the vertices of the species tree, whereas gene duplications ($\square$) are mapped to the edges of *S*. The reciprocal best match graph (RBMG) $(G,\sigma )$ on the right corresponds to the undirected graph underlying the symmetric part of the best match graph (BMG) $(\vec {G},\sigma )$ (third panel)

We consider best matches as an evolutionary concept: A gene *y* in species *s* is a best match of a gene *x* from species $r\ne s$ if *s* contains no gene $y'$ that is more closely related to *x*. That is, best matches capture the idea of phylogenetically most closely related genes. Maybe surprisingly, the combinatorial structure of best matches has become a focus only very recently Geiß et al. (2019). Best match graphs (BMGs) have several appealing properties: They have several alternative characterizations providing polynomial-time recognition algorithms Geiß et al. (2020a), Schaller et al. (2020) and they are “explained” by a unique least resolved tree Geiß et al. (2019). These properties will be introduced formally in the next section and play an important role in our discussion. The reciprocal best match graphs (RBMGs) are the symmetric parts of BMGs and conceptually correspond to the reciprocal best hits used in orthology detection. In contrast to BMGs, RBMGs are much more difficult to handle and are not associated with unique trees Geiß et al. (2020c). An example for an evolutionary scenario with corresponding BMG and RBMG is given Fig. 1.

In this contribution, we are only concerned with the second source of errors, i.e., with the limits in the reconstruction of the true orthology relation from best matches. We therefore assume throughout that a “correct” BMG (cf. Def. 2) is given. *We do not assume, however, that we have any* a priori *knowledge about the underlying gene or species tree*. The problem we aim to solve is to determine the orthology relation that is best supported by the given BMG.

Of course, the *true* orthology relation is not known. Nevertheless, we start our mathematical analysis with the following definition: A pair of genes *x* and *y* that are not true orthologs but reciprocal best matches are false-positive orthologs. If they are orthologs but not reciprocal best matches, they are false-negative orthologs. Geiß et al. (2020b) showed that, for evolutionary scenarios that involve only speciations, gene duplications, and gene losses, there are no false-negative orthology assignments (see also Thm. 2 below). Our task therefore reduces to understanding the false-positive orthology assignments. Being a false positive is a property of the edge *xy* in an RBMG, and equivalently of the symmetric pair (*x*, *y*) and (*y*, *x*) in the BMG. Here, we aim to identify false-positive edges from the structure of the BMG itself.

**Fig. 2:**
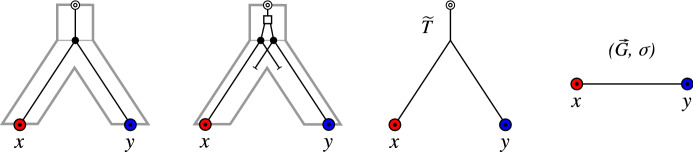
Two scenarios (1st and 2nd panel to the left) for the evolution of a gene family embedded into a species tree (shown in gray), where $\newmoon$ represents speciation and $\square$ duplication events. The second scenario is the simplest example for a complementary gene loss that is not witnessed by any other species. In particular, the two different true histories result in the same topology ${\widetilde{T}}$ of the true (loss-free) gene tree, and thus explain the same BMG $(\vec {G},\sigma )$. However, only for the leftmost scenario the edge *xy* in $(\vec {G},\sigma )$ describes correct orthologs

We first note that false positives cannot be avoided altogether, i.e., not all false positives can be identified from a BMG alone. The simplest example, Fig. 2 (second scenario), comprises a gene duplication and a subsequent speciation and complementary gene losses in the descendant lineages such that each paralog survives only in one of them. In this situation, *xy* is a reciprocal best match. If there are no other descendants that harbor genes witnessing the duplication event, then the framework of best matches provides no information to recognize *xy* as a false-positive assignment.

On the other hand, RBMGs and thus BMGs contain at least some information on false positives. Since the orthology relation forms a cograph but RBMGs are not cographs in general Geiß et al. (2020c), incorrect orthology assignments are associated with induced $P_4$s, the forbidden subgraphs that characterize cographs. $P_4$s arise for instance as a consequence of the complete loss of different paralogous groups in disjoint lineages. Dessimoz et al. (2006) noted that such false-positive orthology assignments can be identified under certain circumstances, in particular, if there is some species in which both paralogs have survived. The corresponding motif in BMGs, the “good quartets”, was investigated in some detail by Geiß et al. (2020c). The removal of such false-positive orthologs already leads to a substantial improvement of the orthology assignments in simulated data Geiß et al. (2020b). Here, we extend the results of Geiß et al. (2020b) to a complete characterization of false-positive orthology assignments for a given BMG.

Good quartets cannot be defined on RBMGs because information on non-reciprocal best matches is also needed explicitly. This suggests to consider BMGs rather than RBMGs as the first step in graph-based orthology detection methods. In practice, best matches are approximated by sequence similarity and thus are subject to noise and biases Stadler et al. (2020). The empirically determined best match relation thus will usually need to be corrected to conform to the formal definition (cf. Def. 2 below) of BMGs. This naturally leads to a graph editing problem that was recently shown to be NP-complete Schaller et al. (2020), Hellmuth et al. (2020b).

Sec. 2 establishes the notation and summarizes properties of BMGs that are needed throughout this contribution. Sec. 3 formalizes the notion of *unambiguous false-positive* (*u-fp*) edges, i.e., reciprocal best matches that cannot be orthologs w.r.t. to *any* gene tree explaining the BMG. Sec. 4 contains the main mathematical contributions of this work:

1. We provide a full characterization of unambiguous false-positive orthology assignments in BMGs.
2. We provide a polynomial-time algorithm to determine all unambiguous false-positive orthology assignments in BMGs.

In Sec. 5, we complement the mathematical results with a computational analysis of simulated scenarios and observe that at least three quarters of all false positives fall into this class. The remaining cases are not recognizable from best matches alone and correspond to complementary losses without surviving witnesses, i.e., cases that cannot be corrected without additional knowledge on the gene tree and/or the species tree.

Since the material is extensive and very technical, we subdivide our presentation into a main narrative part (Secs. 1–6) and a technical part (Secs. A–D) that contains all proofs and additional material in full detail. Together with the definitions and preliminaries in Sec. 2, the technical part is self-contained. Definitions and results appearing in the narrative part are therefore restated. The order of the material in the two parts is slightly different.

## Preliminaries

### Graphs and trees

We consider finite, directed graphs $\vec {G}=(V,E)$, for brevity just called graphs throughout, with arc set $E\subseteq V\times V{\setminus }\{(v,v)\mid v\in V\}$. We say that *xy* is an *edge* in $\vec {G}$ if and only if both $(x,y)\in E(\vec {G})$ and $(y,x)\in E(\vec {G})$. If all arcs of $\vec {G}$ in a graph form edges, we call $\vec {G}$ *undirected*. A graph $H=(W,F)$ is a *subgraph* of $G=(V,E)$, in symbols $H\subseteq G$, if $W\subseteq V$ and $F\subseteq E$. The underlying *symmetric part* of a directed graph $\vec {G}=(V,E)$ is the subgraph $G=(V,F)$ that contains all edges of $\vec {G}$. A subgraph $H=(W,F)$ (of $\vec {G}$) is called *induced*, denoted by $\vec {G}[W]$, if for all $u,v\in W$ it holds that $(u,v) \in E$ implies $(u,v) \in F$. In addition, we consider *vertex-colored* graphs $(\vec {G},\sigma )$ with vertex-coloring $\sigma :V\rightarrow M$ into some set *M* of colors. A vertex-coloring is called *proper* if $\sigma (x)\ne \sigma (y)$ for every arc (*x*, *y*) in $\vec {G}$. We write $\sigma (W) = \{\sigma (w) \mid w\in W\}$ for subsets $W\subseteq V$ and $\sigma _{|W}$ to denote the restriction of the map $\sigma$ to $W\subseteq V$. In particular, $(\vec {G}[W],\sigma _{|W})$ is an induced vertex-colored subgraph of $(\vec {G},\sigma )$.

A *path (of length* $\ell$*)* in a directed graph $\vec {G}$ or an undirected graph *G* is a subgraph induced by a nonempty sequence of pairwise distinct vertices $P(x_0,x_{\ell }) :=(x_0, x_1, \dots , x_{\ell })$ such that $(x_i, x_{i+1}) \in E(\vec {G})$ or $x_ix_{i+1} \in E(G)$, resp., for $0 \le i \le \ell -1$. We use the notation $P(x_0,x_{\ell })$ both for the sequence of vertices and the subgraph they induce.

All *trees* $T=(V,E)$ considered here are *undirected*, *planted* and *phylogenetic*, that is, they satisfy (i) the root $0_T$ has degree 1 and (ii) all inner vertices have degree $\deg _T(u)\ge 3$. We write *L*(*T*) for the leaves (not including $0_T$) and $V^0=V(T){\setminus }(L(T)\cup \{0_T\})$ for the inner vertices (also not including $0_T$). To avoid trivial cases, we will always assume $|L(T)|\ge 2$. An edge *uv* in *T* is an inner edge if $u,v\in V^0(T)$ are inner vertices. The *conventional root* $\rho _T$ of *T* is the unique neighbor of $0_T$. The main reason for using planted phylogenetic trees instead of modeling phylogenetic trees simply as rooted trees, which is the much more common practice in the field, is that we will often need to refer to the time before the first branching event, i.e., the edge $0_T\rho _T$.

We define the *ancestor order* on a given tree *T* as follows: if *y* is a vertex of the unique path connecting *x* with the root $0_T$, we write $x\preceq _T y$, in which case *y* is called an ancestor of *x* and *x* is called a descendant of *y*. We use $x \prec _T y$ for $x \preceq _{T} y$ and $x \ne y$. If $x \preceq _{T} y$ or $y \preceq _{T} x$ the vertices *x* and *y* are *comparable* and, otherwise, *incomparable*. If *xy* is an edge in *T*, such that $y \prec _{T} x$, then *x* is the *parent* of *y* and *y* the *child* of *x*. We denote by $\mathsf {child}_T(x)$ the set of all children of *x*. It will be convenient for the discussion below to extend the ancestor relation $\preceq _T$ to the union of the edge and vertex sets of *T*. More precisely, for a vertex $x\in V(T)$ and an edge $e=uv\in E(T)$ with $v\prec _T u$ we write $x \prec _T e$ if and only if $x\preceq _T v$ and $e \prec _T x$ if and only if $u\preceq _T x$. For edges $e=uv$ with $v\prec _T u$ and $f=ab$ with $b\prec _T a$ in *T* we put $e\preceq _T f$ if and only if $v \preceq _T b$.

For a non-empty subset $A\subseteq V\cup E$, we define ${{\,\mathrm{lca}\,}}_T(A)$, the *last common ancestor of* *A*, to be the unique $\preceq _T$-minimal vertex of *T* that is an ancestor of every vertex or edge in *A*. For simplicity we drop the brackets and write ${{\,\mathrm{lca}\,}}_T(x_1,\dots ,x_k):={{\,\mathrm{lca}\,}}_T(\{x_1,\dots ,x_k\})$ whenever we specify a set of vertices or edges explicitly.

A vertex $v\in V(T)$ is *binary* if $\deg _T(v)=3$, i.e., if *v* has exactly two children. A tree is *binary*, if all vertices $v\in V^0$ are binary. For $v\in V(T)$ we denote by *T*(*v*) the subtree of *T* rooted in *v*. The set of *clusters* of a tree *T* is ${\mathscr {C}}(T) = \{L(T(v))\mid v\in V(T)\}$. It is well-known that ${\mathscr {C}}(T)$ uniquely determines *T*Semple and Steel (2003). We say that a tree *T* is a *refinement* of some tree $T'$ if ${\mathscr {C}}(T')\subseteq {\mathscr {C}}(T)$. A tree $T'$ is *displayed* by a tree *T*, in symbols $T'\le T$, if $T'$ can be obtained from a subtree of *T* by contraction of edges Semple (2003), where the contraction of an edge $e = uv$ in a tree $T = (V ,E)$ refers to the removal of *e* and identification of *u* and *v*. It is easy to verify that every refinement *T* of $T'$ also displays $T'$. However, the converse is not always true since $L(T')\subsetneq L(T)$ and thus, ${\mathscr {C}}(T')\not \subseteq {\mathscr {C}}(T)$ may be possible.

### (Reciprocal) best matches

We consider a pair $T=(V,E)$ and $S=(W,F)$ of planted phylogenetic trees together with a map $\sigma :L(T)\rightarrow L(S)$. We interpret *T* as a *gene tree* and *S* as a *species tree*; the map $\sigma$ describes, for each gene $x\in L(T)$, in the genome of which species $\sigma (x)\in L(S)$ it resides. W.l.o.g. we assume that the “gene-species-association” $\sigma$ is a surjective map to avoid trivial cases. Since $\sigma$ can be viewed as a coloring of the leaves of *T*, we call $(T,\sigma )$ a *leaf-colored tree*. For $s\in L(S)$ we write $L[s]:=\{x\in L(T)|\sigma (x)=s\}$.

#### Definition 1

Let $(T,\sigma )$ be a leaf-colored tree. A leaf $y\in L(T)$ is a *best match* of the leaf $x\in L(T)$ if $\sigma (x)\ne \sigma (y)$ and ${{\,\mathrm{lca}\,}}(x,y)\preceq _T {{\,\mathrm{lca}\,}}(x,y')$ holds for all leaves $y'$ from species $\sigma (y')=\sigma (y)$. The leaves $x,y\in L(T)$ are *reciprocal best matches* if *y* is a best match for *x* and *x* is a best match for *y*.

Neither best matches nor reciprocal best matches are unique. That is, a gene *x* may have two or more (reciprocal) best matches of the same color $r\ne \sigma (x)$. Some orthology detection tools, such as ProteinOrtho Lechner et al. (2011), explicitly attempt to extract all reciprocal best matches from the sequence data. Moreover, neither of the two relations is transitive. These two properties are at odds e.g. with the *clusters of orthologous groups* (COGs) concept (cf. Tatusov et al. 1997, 2000; Roth et al. 2008), which at least conceptually presupposes unique reciprocal best matches.

The graph $\vec {G}(T,\sigma ) = (V,E)$ with vertex set $V=L(T)$, vertex coloring $\sigma$, and with arcs $(x,y)\in E$ if and only if *y* is a best match of *x* w.r.t. $(T,\sigma )$ is known as the (colored) *best match graph* of $(T,\sigma )$ Geiß et al. (2019). The symmetric part $G(T,\sigma )$ of $\vec {G}(T,\sigma )$ obtained by retaining the edges of $\vec {G}(T,\sigma )$ is the (colored) *reciprocal best match graph* Geiß et al. (2020c).

#### Definition 2

An arbitrary vertex-colored graph $(\vec {G},\sigma )$ is a *best match graph (BMG)* if there exists a leaf-colored tree $(T,\sigma )$ such that $(\vec {G},\sigma ) = \vec {G}(T,\sigma )$. In this case, we say that $(T,\sigma )$ *explains* $(\vec {G},\sigma )$. An arbitrary undirected vertex-colored graph $(G,\sigma )$ is a *reciprocal best match graph (RBMG)* if it is the symmetric part of a BMG $(\vec {G},\sigma )$.

For the symmetric part of the BMG $(\vec {G},\sigma )$, i.e., the RBMG $(G,\sigma )$, we have $xy\in E(G)$ if and only if *x* and *y* are reciprocal best matches in $(T,\sigma )$. In this sense, $(T,\sigma )$ also explains $(G,\sigma )$. We note, furthermore, that RBMGs are not associated with a unique least resolved tree Geiß et al. (2020c).

### Reconciliation maps, event-labeling, and orthology relations

An *evolutionary scenario* extends the map $\sigma :L(T)\rightarrow L(S)$ to an embedding of the gene tree into the species tree. It (implicitly) describes different types of evolutionary events: speciations, gene duplications, and gene losses. In this contribution we do not consider other types of events such as horizontal gene transfer. Gene losses do not appear explicitly since *L*(*T*) only contains extant genes. Inner vertices in the gene tree *T* that designate speciations have their correspondence in inner vertices of the species tree. In contrast, gene duplications occur independently of speciations and thus belong to edges of the species tree. The embedding of *T* into *S* is formalized by

#### Definition 3

*(Reconciliation Map)* Let $S=(W,F)$ and $T=(V,E)$ be two planted phylogenetic trees and let $\sigma :L(T) \rightarrow L(S)$ be a surjective map. A reconciliation from $(T,\sigma )$ to *S* is a map $\mu :V \rightarrow W \cup F$ satisfying

*(R0)*: *Root Constraint.* $\mu (x) = 0_S$ if and only if $x=0_T$.
*(R1)*: *Leaf Constraint.* If $x \in L(T)$, then $\mu (x)=\sigma (x)$.
*(R2)*: *Ancestor Preservation.* If $x \prec _T y$, then $\mu (x) \preceq _S \mu (y)$.
*(R3)*: *Speciation Constraints.* Suppose $\mu (x) \in W^0$ for some $x\in V$. Then
  (i): $\mu (x)={{\,\mathrm{lca}\,}}_S(\mu (v'),\mu (v''))$ for at least two distinct children $v',v''$ of *x* in *T*.
  (ii): $\mu (v')$ and $\mu (v'')$ are incomparable in *S* for any two distinct children $v'$ and $v''$ of *x* in *T*.

Several alternative definitions of reconciliation maps for duplication/loss scenarios have been proposed in the literature, many of which have been shown to be equivalent. This type of reconciliation map has been established in Geiß et al. (2020b). Moreover, it has been shown in Geiß et al. (2020b) that the axiom set used here is equivalent to axioms that are commonly used in the literature, see e.g. Górecki and Tiuryn (2006), Vernot et al. (2008), Doyon et al. (2011), Rusin et al. (2014), Hellmuth (2017), Nøjgaard et al. (2018), and the references therein. Without any further constraints, Def. 3 gives rise to a well-known result:

#### Lemma 1

(Geiß et al. 2020b, Lemma 3) For every tree $(T, \sigma )$ there is a reconciliation map $\mu$ to any species tree *S* with leaf set $L(S) = \sigma (L(T ))$.

The reconciliation map $\mu$ from $(T,\sigma )$ to *S* determines the types of evolutionary events in *T*. This can be formalized by associating an event labeling with the vertices of *T*. We use the notation introduced in Geiß et al. (2020b):

#### Definition 4

Given a reconciliation map $\mu$ from $(T,\sigma )$ to *S*, the *event labeling on* *T* *(determined by* $\mu$*)* is the map $t_\mu :V(T)\rightarrow \{\circledcirc ,\odot ,\newmoon ,\square \}$ given by:

$$\begin{aligned} t_\mu (u) = {\left\{ \begin{array}{ll} \circledcirc &{} \, \text {if } u=0_T \text {, i.e., } \mu (u)=0_S \text { (root)}\\ \odot &{} \, \text {if } u\in L(T) \text {, i.e., } \mu (u)\in L(S) \text { (leaf)}\\ \newmoon &{} \, \text {if } \mu (u)\in V^0(S) \text { (speciation)}\\ \square &{} \, \text {else, i.e., } \mu (u)\in E(S) \text { (duplication)}\\ \end{array}\right. } \end{aligned}$$

The following result is a simple but useful consequence of combining the axioms of the reconciliation map with the event labeling of Def. 4.

#### Lemma 2

(Geiß et al. 2020b, Lemma 3) Let $\mu$ be a reconciliation map from $(T,\sigma )$ to a tree *S* and suppose that $u\in V(T)$ is a vertex with $\mu (u)\in V^0(S)$ and thus, $t(\mu (u))=\newmoon$. Then, $\sigma (L(T(v_1)))\cap \sigma (L(T(v_2))) = \emptyset$ for any two distinct $v_1,v_2\in \mathsf {child}(u)$.

We will regularly make use of the observation that, by contraposition of Lemma 2, $\sigma (L(T(v)))\cap \sigma (L(T(v'))) \ne \emptyset$ for two distinct $v_1,v_2\in \mathsf {child}(u)$ implies that $\mu (u)\in E(S)$, and thus $t_{\mu }(u)=\square$.

Lemma 2 suggests to define *event-labeled trees* as trees (*T*, *t*) endowed with a map $t: V(T)\rightarrow \{\circledcirc ,\odot ,\newmoon ,\square \}$ such that $t(0_T)=\circledcirc$ and $t(u)=\odot$ for all $u\in L(T)$. In Geiß et al. (2020b), Lemma 2 also served as a motivation for

#### Definition 5

Let $(T,\sigma )$ be a leaf-colored tree. The *extremal event labeling* of *T* is the map ${{\widehat{t}}_T}:V(T)\rightarrow \{\circledcirc ,\odot ,\newmoon ,\square \}$ defined for $u\in V(T)$ by

$$\begin{aligned} {{\widehat{t}}_T}(u) = {\left\{ \begin{array}{ll} \circledcirc &{} \, \text {if } u=0_{T} \\ \odot &{} \, \text {if } u\in L(T) \\ \square &{} \, \text {if there are two children } v_1,v_2\in \mathsf {child}(u) \text { such that}\\ &{} \qquad \sigma (L(T(v_1)))\cap \sigma (L(T(v_2)))\ne \emptyset \\ \newmoon &{} \, \text {otherwise} \\ \end{array}\right. } \end{aligned}$$

An example of an extremal event labeling is shown in Fig. 9 (rightmost tree). The extremal event labeling is closely related to the concept of apparent duplication (AD) vertices often found in the literature (e.g. Swenson et al. 2012; Lafond et al. 2014). For a (binary) gene tree *T* and a reconciliation of *T* with a species tree *S*, a duplication vertex of *T* is an AD vertex if its two subtrees have at least one color in common. In contrast, it is a non-apparent duplication (NAD) vertex if the color sets of its subtrees are disjoint. This notion is useful for a variety of parsimony problems that usually aim to avoid or minimize the number of NAD vertices Swenson et al. (2012), Lafond et al. (2014). However, the extremal event labeling ${{\widehat{t}}_T}$ is completely defined by $(T,\sigma )$. That is, in contrast to both the event labeling in Def. 4 and the concept of AD and NAD vertices, ${{\widehat{t}}_T}$ does not depend on a specific reconciliation map. On the other hand, there is no guarantee that there always exists a reconciliation map $\mu$ from $(T,\sigma )$ to some species tree *S* such that $t_{\mu } = {{\widehat{t}}_T}$, cf. (Geiß et al. 2020b, Fig. 2) and Fig. 9 in Sec. 4.2 for counterexamples. Nevertheless, we shall see below that the extremal labeling is a key step towards identifying false-positive orthology assignments.

The event labeling on *T* defines the orthology graph.

#### Definition 6

The *orthology graph* $\Theta (T,t)$ of an event-labeled tree (*T*, *t*) has vertex set *L*(*T*) and edges $uv\in E(\Theta )$ if and only if $t({{\,\mathrm{lca}\,}}(u,v))=\newmoon$.

The orthology graph is often referred to as the orthology relation. Orthology graphs coincide with a well-known graph class:

#### Theorem 1

(Hellmuth et al. 2013, Cor. 4) A graph *G* is an orthology graph for some event-labeled tree (*T*, *t*), i.e. $G=\Theta (T,t)$, if and only if *G* is a cograph.

One of many equivalent characterizations of cographs identifies them with the graphs that do not contain an induced path $P_4$ on four vertices Corneil et al. (1981).

The orthology graph is a subgraph of the RBMG (and thus also of the BMG) for any given reconciliation map connecting a gene with a species tree.

#### Theorem 2

(Geiß et al. 2020b, Lemma 4 & 5) Let $(T,\sigma )$ be a leaf-colored tree and $\mu$ a reconciliation map from $(T,\sigma )$ to some species tree *S*. Then $\Theta (T,t_{\mu }) \subseteq \Theta (T,{{\widehat{t}}_T})\subseteq G(T,\sigma ) \subseteq \vec {G}(T,\sigma )$.

In particular, $t_{\mu }(v) =\newmoon$ implies ${{\widehat{t}}_T}(v) =\newmoon$ for any reconciliation map. By contraposition, therefore, if ${{\widehat{t}}_T}(v) =\square$ then $t_{\mu }(v) =\square$ for all possible reconciliation maps $\mu$ from $(T,\sigma )$ to any species tree *S*. A crucial implication of Thm. 2 is that edges in a BMG $\vec {G}(T,\sigma )$ always correspond to either correct orthologous pairs of genes or false-positive orthology assignments. Hence, $\vec {G}(T,\sigma )$ never contains false-negative orthology assignments.

## False-positive orthology assignments

As discussed in the introduction, we are not concerned here with the errors that arise in the reconstruction of best matches from sequence similarity data. We therefore assume that we are given a BMG $(\vec {G},\sigma )$ as specified in Def. 2. More precisely, we assume that $(\vec {G},\sigma )$ derives from a duplication/loss scenario that is unknown to us. Denote by $({\widetilde{T}},{\widetilde{t}},\sigma )$ the corresponding true leaf-colored and event-labeled gene tree. An edge *xy* of $(\vec {G},\sigma )$, or equivalently of the corresponding RBMG $(G,\sigma )$, is a false-positive orthology assignment if $xy\in E(G)$ but $xy\notin E(\Theta ({\widetilde{T}},{\widetilde{t}}))$. By Thm. 2, $(G,\sigma )$ cannot contain false-negative orthology assignments, i.e., there is no $xy\in E(\Theta ({\widetilde{T}},{\widetilde{t}}))$ with $xy\notin E(G)$. We assume no additional information about the gene tree or the species tree, i.e., the only data about the evolutionary scenario that is available to us is the BMG $(\vec {G},\sigma )$.

In order to study false-positive orthology assignments, we first consider a tree $(T,\sigma )$ that explains the BMG $(\vec {G},\sigma )$. We neither make the assumption that $(T,\sigma )$ is least resolved nor that $(T,\sigma )$ reflects the true history, i.e., that $(T,\sigma )$ is related to the true gene tree $({\widetilde{T}},\sigma )$.

### Definition 10

*(*${(T,\sigma )}$-*false-positive)* Let $(T,\sigma )$ be a tree explaining the BMG $(\vec {G},\sigma )$. An edge *xy* in $\vec {G}$ is called $(T,\sigma )$-*false-positive*, or $(T,\sigma )$-*fp* for short, if for every reconciliation map $\mu$ from $(T,\sigma )$ to any species tree *S* we have $t_\mu ({{\,\mathrm{lca}\,}}_T(x,y))=\square$, i.e., $\mu ({{\,\mathrm{lca}\,}}_T(x,y))\in E(S)$,

In other words, *xy* is called $(T,\sigma )$-*fp* whenever *x* and *y* cannot be orthologous w.r.t. any possible reconciliation $\mu$ from $(T,\sigma )$ to any species tree. Interestingly, $(T,\sigma )$-*fp* s can be identified without considering reconciliation maps explicitly.

### Lemma 10

Let $(\vec {G},\sigma )$ be a BMG, *xy* be an edge in $\vec {G}$ and $(T,\sigma )$ be a tree that explains $(\vec {G},\sigma )$. Then, the following statements are equivalent:

1. The edge *xy* is $(T,\sigma )$-*fp*.
2. There are two children $v_1$ and $v_2$ of ${{\,\mathrm{lca}\,}}_T(x,y)$ such that $\sigma (L(T(v_1)))\cap \sigma (L(T(v_2)))\ne \emptyset$.
3. For the extremal labeling ${{\widehat{t}}_T}$ of $(T,\sigma )$ it holds that ${{\widehat{t}}_T}({{\,\mathrm{lca}\,}}_T(x,y)) = \square$.

Lemma 10 implies that $(T,\sigma )$-*fp* can be verified in polynomial time for any given gene tree $(T,\sigma )$. By contraposition of Lemma 2, inner vertices with two distinct children $v_1$ and $v_2$ satisfying $\sigma (L(T(v_1)))\cap \sigma (L(T(v_2)))\ne \emptyset$ are duplication vertices for every possible reconciliation map to every possible species tree. Therefore, the property of being an AD vertex only depends on $(T,\sigma )$. In particular, $(T,\sigma )$-*fp* edges coincide with the edges *xy* in $(\vec {G},\sigma )$ for which ${{\,\mathrm{lca}\,}}_{T}(x,y)$ is an AD vertex.

**Fig. 3:**
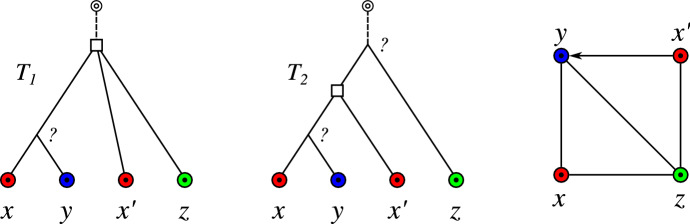
The BMG $(\vec {G},\sigma )$ shown on the right is explained by both $(T_1,\sigma )$, which is the unique least resolved tree for $(\vec {G},\sigma )$, and $(T_2,\sigma )$. The vertices labeled $\square$ must be duplications due to Lemma 2, whereas the vertices labeled “?” could be both duplications or speciations. The edges *xz*, $x'z$ and *yz* are $(T_1,\sigma )$-*fp* but not $(T_2,\sigma )$-*fp* (cf. Lemma 10). Thus, neither of the edges *xz*, $x'z$ and *yz* is *u-fp*

As shown in Fig. 3, there are trees $(T_1,\sigma )$ and $(T_2,\sigma )$ that explain the same BMG for which, however, the edges *xz*, $x'z$, and *yz* are $(T_1,\sigma )$-*fp* but not $(T_2,\sigma )$-*fp*. Since we assume that no information on $(T,\sigma )$ is available *a priori*, it is natural to consider the set of edges that are false positives for all trees explaining a given BMG.

### Definition 11

*(Unambiguous false-positive)* Let $(\vec {G},\sigma )$ be a BMG. An edge *xy* in $\vec {G}$ is called *unambiguous false-positive (u-fp)* if for all trees $(T,\sigma )$ that explain $(\vec {G},\sigma )$ the edge *xy* is $(T,\sigma )$-*fp*.

Hence, if an edge *xy* in $\vec {G}$ is *u-fp*, then it is in particular $(T,\sigma )$-*fp* in the true history that explains $(\vec {G},\sigma )$. Thus, *u-fp* edges are always correctly identified as false positives. Not all “correct” false-positive edges are *u-fp*, however. It is possible that, for an edge *xy* in $\vec {G}$, we have $t_\mu ({{\,\mathrm{lca}\,}}_T(x,y))=\square$ for the true gene tree and the true species tree, but *xy* is not $(T',\sigma )$-*fp* for some gene tree $(T',\sigma )$ possibly different from $(T,\sigma )$. One of the simplest examples is shown in Fig. 2, assuming that $(\vec {G},\sigma )$ is the “true” BMG. Since $t_\mu ({{\,\mathrm{lca}\,}}_{{\widetilde{T}}}(x,y))=\newmoon$ may be possible (Fig. 2, leftmost scenario, the edge *xy* is not $({\widetilde{T}},\sigma )$-*fp* and therefore not *u-fp*.

## Main results

### Characterization of *u-fp* edges

In order to adapt the concept of AD vertices for our purposes, we introduce the color-intersection ${\mathcal {S}}^{\cap }$ associated with a gene tree $(T,\sigma )$. For a pair of distinct leaves $x,y\in L(T)$ we denote by $v_x, v_y \in \mathsf {child}_T({{\,\mathrm{lca}\,}}_T(x,y))$ the unique children of the last common ancestor of *x* and *y* for which $x\preceq _T v_x$ and $y\preceq _T v_y$. That is, $T(v_x)$ and $T(v_y)$ are the subtrees of *T* rooted in the children of ${{\,\mathrm{lca}\,}}_T(x,y)$ with $x\in L(T(v_x))$ and $y\in L(T(v_y))$. The set

$$\begin{aligned} {\mathcal {S}}_T^{\cap }(x,y):=\sigma (L(T(v_x)))\cap \sigma (L(T(v_y))) \end{aligned}$$

contains the colors, i.e. species, that are common to both subtrees. The existence of common colors, ${\mathcal {S}}_T^{\cap }(x,y)\ne \emptyset$, determines whether or not the inner vertex ${{\,\mathrm{lca}\,}}_T(x,y)$ is AD. Lemma 11 (Sec. B.2) shows that the color-intersection ${\mathcal {S}}_T^{\cap }(x,y)$ of an edge in a BMG $(\vec {G},\sigma )$ is independent of the corresponding tree. Hence, it suffices to consider the color-intersection for the unique least resolved tree $(T^*,\sigma )$ explaining $(\vec {G},\sigma )$. From here on, we drop the explicit reference to the tree and simply write ${\mathcal {S}}^{\cap }(x,y)$; see also Remark 1 in Sec. B.2. The color-intersection provides a sufficient condition for *u-fp* edges in a BMG.

#### Prop. 1 and Cor. 3

Every edge *xy* in a BMG $(\vec {G},\sigma )$ with ${\mathcal {S}}^{\cap }(x,y)\ne \emptyset$ is $(T,\sigma )$-*fp* for every tree $(T,\sigma )$ that explains $(\vec {G},\sigma )$, and thus *u-fp*.

As we shall see below, the converse of Prop. 1 and Cor. 3 is not true in general. It does hold for the special case of binary trees, however:

#### Theorem 4

Let $(\vec {G},\sigma )$ be a BMG that is explained by a binary tree $(T,\sigma )$. Then, for every edge *xy* in $(\vec {G},\sigma )$, the following three statements are equivalent:

1. The edge *xy* is $(T,\sigma )$-*fp*.
2. $${\mathcal {S}}^{\cap }(x,y)\ne \emptyset $$.
3. The edge *xy* is *u-fp*.

Prop. 8 in Sec. 4.3 provides a characterization of BMGs that can be explained by binary trees; a property that can be tested in polynomial time (cf. Cor. 6). However, not every BMG can be explained by a binary tree as shown by the simple example in Fig. 6(A). This BMG can only be explained by the unique non-binary tree as shown in Fig. 6(B).

Since every orthology graph is a cograph (Thm. 1) and thus free of induced $P_4$s, every induced $P_4$ in the RBMG necessarily contains a false-positive orthology assignments. The subgraphs of the BMG spanned by a $P_4$ in its symmetric part (i.e., the RBMG) are known as quartets. The quartets on three colors of a BMG $(\vec {G},\sigma )$ fall into three distinct classes depending on the coloring and the additional, non-symmetric edges (cf. (Geiß et al. 2020c, Lemma 32)). We write $\langle abcd \rangle$ or, equivalently, $\langle dcba \rangle$ for an induced $P_4$ with edges *ab*, *bc*, and *cd*.

#### Definition 12

*(Good, bad, and ugly quartets)* Let $(\vec {G},\sigma )$ be a BMG with symmetric part $(G,\sigma )$ and vertex set *L*, and let $Q:=\{x,y,z,z'\} \subseteq L$ with $x\in L[r]$, $y\in L[s]$, and $z,z'\in L[t]$. The set *Q*, resp., the induced subgraph $(\vec {G}[Q],\sigma _{|Q})$ is

- a *good quartet* if (i) $\langle zxyz'\rangle$ is an induced $P_4$ in $(G,\sigma )$ and (ii) $(z,y),(z',x)\in E(\vec {G})$ and $(y,z),(x,z')\notin E(\vec {G})$,
- a *bad quartet* if (i) $\langle zxyz'\rangle$ is an induced $P_4$ in $(G,\sigma )$ and (ii) $(y,z),(x,z')\in E(\vec {G})$ and $(z,y),(z',x)\notin E(\vec {G})$,
- an *ugly quartet* if $\langle zxz'y\rangle$ is an induced $P_4$ in $(G,\sigma )$.

The edge *xy* in a good quartet $\langle zxyz'\rangle$ is its *middle* edge. The edge *zx* of an ugly quartet $\langle zxz'y\rangle$ or a bad quartet $\langle zxyz'\rangle$ is called its *first* edge. First edges in ugly quartets are uniquely determined due to the colors. In bad quartets, this is not the case and therefore, the edge $yz'$ in $\langle zxyz'\rangle$ is a first edge as well.

**Fig. 4:**
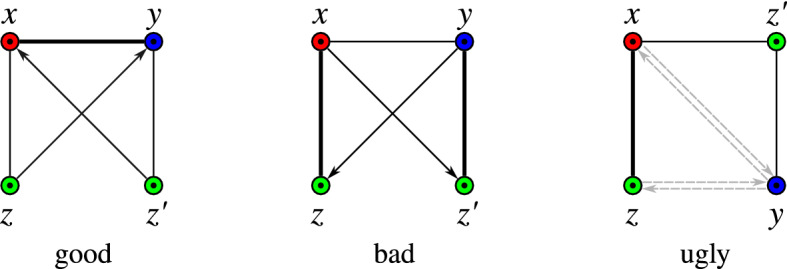
The three types of quartets in BMGs. Ugly quartets may or may not contain either of the two (dashed) arcs between *x* and *y*, and *y* and *z*, respectively. Bold edges highlight the middle and first edges of the respective quartets as specified in Def. 12

The three different types of quartets are shown in Fig. 4. RBMGs never contain induced $P_4$s on two colors (Geiß et al. 2020c, Obs. 5). This, in particular, implies that for the induced $P_4$s in Def. 12 the colors *r*, *s*, and *t* must be pairwise distinct. Note that (R)BMGs may also contain induced $P_4$s on four colors. These are investigated in some more detail in Secs. 4.3 and D.3.

Good quartets are characteristic of a complementary gene loss (as shown in Fig. 2) that is “witnessed” by a third species in which both child branches of the problematic duplication event survive. That is, good quartets appear if there is a pair of genes *z* and $z'$ with $\sigma (z)=\sigma (z')$ and ${{\,\mathrm{lca}\,}}(z,z')={{\,\mathrm{lca}\,}}(x,y)$ in the true gene tree. We remark that previous work also noted that complementary gene loss can be resolved successfully under certain circumstances Dessimoz et al. (2006) such as this one. An in-depth analysis of quartets shows that they can be used to identify many of the *u-fp* edges. We collect here the main results of Sec. B.3:

#### Prop. 2, 3 and 4

Let ${\mathcal {Q}} = \langle xyzw \rangle$ be a quartet in a BMG $(\vec {G},\sigma )$.

(i) If ${\mathcal {Q}}$ is good, then its middle edge *yz* is *u-fp*.
(ii) If ${\mathcal {Q}}$ is ugly, then its first edge *xy* and its middle edge *yz* are *u-fp*.
(iii) If ${\mathcal {Q}}$ is bad, then its first edges *xy* and *zw* are *u-fp*.

Not surprisingly, quartets are intimately linked to color-intersections:

#### Corollary 4

Let $(\vec {G},\sigma )$ be a BMG that contains the edge *xy*. Then, ${\mathcal {S}}^{\cap }(x,y)\ne \emptyset$ implies that *xy* is either the middle edge of some good quartet or the first edge of some ugly quartet, which in turn implies that *xy* is *u-fp*.

**Fig. 5:**
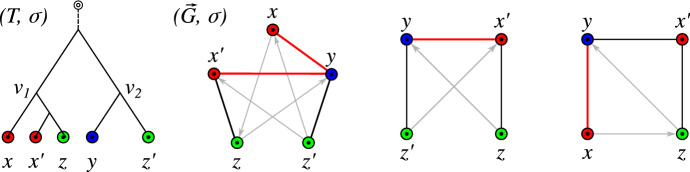
Example for a $(T,\sigma )$-*fp* edge *xy* in $(\vec {G},\sigma )$ which is not the middle edge of a good quartet, but the first edge in an ugly quartet (right). Note, $(\vec {G},\sigma )$ does not contain bad quartets

All *u-fp* edges *xy* with ${\mathcal {S}}^{\cap }(x,y)\ne \emptyset$ in $(\vec {G},\sigma )$ are therefore completely determined by the middle edges of good quartets and the first edges of ugly quartets. In particular, not all such edges are the middle edge of a good quartet as the example in Fig. 5 shows. Therein, the edge *xy* must be *u-fp* since ${\mathcal {S}}^{\cap }(x,y)=\{\sigma (z)\}\ne \emptyset$ (cf. Prop. 1). The only good quartet is $\langle zx'yz'\rangle$ identifying $x'y$ as *u-fp*. Moreover, $(\vec {G},\sigma )$ does not contain any bad quartet. The edge *xy*, on the other hand, is the first edge of the ugly quartet $\langle xyx'z\rangle$.

**Fig. 6:**
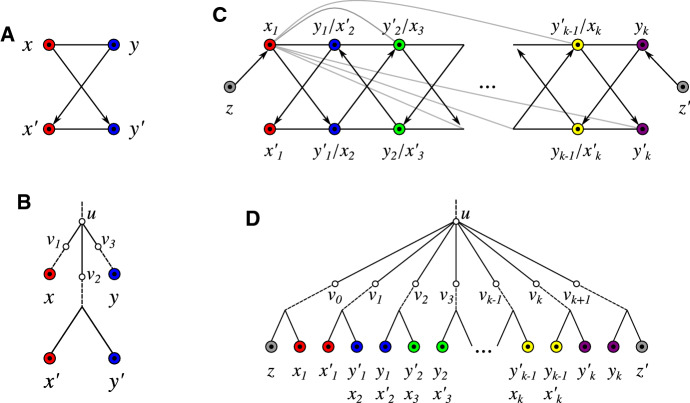
A: Hourglass. B: Visualization of Lemma 14. C: Hourglass chain with left tail *z* and right tail $z'$ for an odd number of hourglasses in the chain. Edges of the form $x_i y'_j\in E(G)$ are only shown for $x_1$, the others are omitted. An hourglass chain ${\mathfrak {H}}$ is a subgraph but not necessarily induced and thus additional arcs may exist. In particular, the elements $e\in \{x_1y_k, zy_k, x_1z', zz'\}$ are not necessarily edges in an hourglass chain. However, whenever they exist, they are *u-fp* (cf. Lemma 17). Moreover, each single hourglass in ${\mathfrak {H}}$ is an induced subgraph of the BMG; by definition, therefore, there are no arcs $(z,x'_1)$ or $(z',y'_k)$. Note, $\sigma (z)\ne \sigma (z')$ is possible. D: Visualization of Lemmas 15 and 16

Furthermore, if an edge *xy* is the middle edge of a good quartet, then ${\mathcal {S}}^{\cap }(x,y)\ne \emptyset$. Therefore, only ugly quartets may provide additional information about *u-fp* edges that are not identified with the help of the color-intersection ${\mathcal {S}}^{\cap }$ (see Fig. 14 in Sec. B.3 for an example). Ugly quartets, however, do not convey all the missing information on *u-fp* edges. The edge *xy* in the BMG shown in Fig. 6(A) is *u-fp*, but it is not contained in a good, bad, or ugly quartet.

In order to characterize the *u-fp* edges that are not identified by quartets, we first introduce an additional motif that may occur in vertex-colored graphs.

#### Definition 13

*(Hourglass)* An *hourglass* in a proper vertex-colored graph $(\vec {G},\sigma )$, denoted by , is a subgraph $(\vec {G}[Q],\sigma _{|Q})$ induced by a set of four pairwise distinct vertices $Q=\{x, x', y, y'\}\subseteq V(\vec {G})$ such that (i) $\sigma (x)=\sigma (x')\ne \sigma (y)=\sigma (y')$, (ii) *xy* and $x'y'$ are edges in $\vec {G}$, (iii) $(x,y'),(y,x')\in E(\vec {G})$, and (iv) $(y',x),(x',y)\notin E(\vec {G})$.

Note that Condition (i) rules out arcs between $x,x'$ and $y,y'$, respectively, i.e., the only arcs in an hourglass are the ones specified by Conditions (ii) and (iii). An example is shown in Fig. 6(A).

#### Observation 5

Every hourglass is a BMG since it can be explained by a tree as shown in Fig. 6(B).

Hourglasses are not necessarily part of an induced $P_4$. In particular, an hourglass does not contain an induced $P_4$ (see Fig. 6(A)).

Hourglasses  can be used to identify false-positive edges *xy* with ${\mathcal {S}}^{\cap }(x,y)=\emptyset$. More precisely, we have

#### Proposition 6

If a BMG $(\vec {G},\sigma )$ contains an hourglass , then the edge *xy* is *u-fp*.

Prop. 6 implies that there are *u-fp* edges that are not contained in a quartet, see Fig. 6(A). In this example, we have ${\mathcal {S}}^{\cap }(x,y)=\emptyset$ and no induced $P_4$. However, as shown in Fig. 6(B), the subtree $T(v_2)$ contains both colors $\sigma (x)$ and $\sigma (y)$ and thus, “bridges” the color sets of the subtrees $T(v_1)$ and $T(v_3)$. Similarly, in the tree $(T,\sigma )$ in Fig. 6(D), each subtree $T(v_i)$, $1\le i \le k$ “bridges” the color sets of the subtrees $T(v_{i-1})$ and $T(v_{i+1})$. This observation suggests the concept of hourglass chains, a generalization of hourglasses.

#### Definition 14

*(Hourglass chain)* An *hourglass chain* ${\mathfrak {H}}$ in a graph $(\vec {G},\sigma )$ is a sequence of $k\ge 1$ hourglasses  such that the following two conditions are satisfied for all $i\in \{1,\dots ,k-1\}$:

1. $$y_i=x'_{i+1}$$ and $$y'_i=x_{i+1}$$, and
2. $x_i y'_j$ is an edge in $\vec {G}$ for all $j\in \{i+1,\dots ,k\}$

A vertex *z* is called a *left* (resp., *right*) *tail* of the hourglass chain ${\mathfrak {H}}$ if it holds that $(z,x_1)\in E(\vec {G})$ and $(z,x'_1)\notin E(\vec {G})$ (resp., $(z,y_k)\in E(\vec {G})$ and $(z,y'_k)\notin E(\vec {G})$). We call ${\mathfrak {H}}$ *tailed* if it has a left or right tail.

In contrast to the quartets and the hourglass, an hourglass chain in $(\vec {G},\sigma )$ is not necessarily an induced subgraph. Hourglass chains are “overlapping” hourglasses. The additional condition that $x_i y'_j\in E(G)$ for all $1\le i<j\le k$ ensures that the two pairs $x'_k,y'_k$ and $x'_l,y'_l$ with $k\ne l$ cannot lie in the same subtree below the last common ancestor *u* which is common to all hourglasses in the chain (cf. Lemma 15 and 16 in Sec. B.4).

#### Definition 16

An edge *xy* in a vertex-colored graph $(\vec {G},\sigma )$ is a *hug-edge* if it satisfies at least one of the following conditions:

1. *xy* is the middle edge of a good quartet in $(\vec {G},\sigma )$;
2. *xy* is the first edge of an ugly quartet in $(\vec {G},\sigma )$; or
3. there is an hourglass chain  in $(\vec {G},\sigma )$, and one of the following cases holds:
  1. $$x_1=x$$ and $$y_k=y$$;
  2. $y_k=y$ and $z:=x$ is a left tail of ${\mathfrak {H}}$;
  3. $x_1=x$ and $z':=y$ is a right tail of ${\mathfrak {H}}$; or
  4. $z:=x$ is a left tail and $z':=y$ is a right tail of ${\mathfrak {H}}$.

The term **hug**-edge refers to the fact that *xy* is a particular edge of an **h**ourglass-chain, an **u**gly quartet, or a **g**ood quartet. In Sec. C.4, we show that hug-edges coincide with the *u-fp* edges.

#### Theorem 11

An edge *xy* in a BMG $(\vec {G},\sigma )$ is *u-fp* if and only if *xy* is a hug-edge of $(\vec {G},\sigma )$.

Interestingly, bad quartets turn out to be redundant for the identification of *u-fp* edges in the sense that every *u-fp* edge in a bad quartet appears as a *u-fp* edge in a good quartet, an ugly quartet, or an hourglass chain. At present, we do not know whether hourglass chains in a colored graph $(\vec {G},\sigma )$ can be found efficiently. We shall see in the following section, however, that the identification of *u-fp* edges does not require the explicit enumeration of hourglass chains.

The fact that all hug-edges are *u-fp* by Thm. 11 suggests to consider the subgraph of a BMG that is left after removing all these unambiguously recognizable false-positive orthology assignments.

#### Definition 17

Let $(\vec {G},\sigma )$ be a BMG with symmetric part *G* and let *F* be the set of its hug-edges. The *no-hug*^1^ graph ${\mathbb {N}}{\mathbb {H}}(\vec {G},\sigma )$ is the subgraph of *G* with vertex set $V(\vec {G})$, coloring $\sigma$ and edge set $E(G){\setminus } F$.

By Thm. 11, ${\mathbb {N}}{\mathbb {H}}(\vec {G},\sigma )$ is therefore the subgraph of the underlying RBMG of $(\vec {G},\sigma )$ that does not contain any *u-fp* edge. Importantly, it contains the orthology graph for every reconciliation map $\mu$ as well as the orthology graph induced by the extremal event labeling as subgraphs:

#### Corollary 5

Let $(T,\sigma )$ be a leaf-colored tree and $\mu$ a reconciliation map from $(T,\sigma )$ to some species tree *S*. Then,

$$\begin{aligned} \Theta (T,t_{\mu }) \subseteq \Theta (T, {{\widehat{t}}_T}) \subseteq {\mathbb {N}}{\mathbb {H}}(\vec {G}(T,\sigma )) \subseteq \vec {G}(T,\sigma ). \end{aligned}$$

The no-hug graph still may contain false-positive orthology assignments, i.e., ${\mathbb {N}}{\mathbb {H}}(\vec {G}(T,\sigma ))=\Theta (T,{{\widehat{t}}_T})$ does not hold in general. As an example, consider the BMG $\vec {G}(T_1,\sigma )$ in Fig. 3. Here, none of the edges *xz*, $x'z$ and *yz* are *u-fp* and thus, by Thm. 11 also not hug-edges. Hence, they still remain in ${\mathbb {N}}{\mathbb {H}}(\vec {G}(T_1,\sigma ))$. However, these edges are not contained in $\Theta (T_1,{{\widehat{t}}_T})$, since ${{\widehat{t}}_T}({{\,\mathrm{lca}\,}}_{T_1}(x,x',y,z)) = \square$ and thus, $\Theta (T_1,{{\widehat{t}}_T}) \subsetneq {\mathbb {N}}{\mathbb {H}}(\vec {G}(T_1,\sigma ))$.

### Algorithms

In this section, we provide a polynomial-time algorithm to identify all *u-fp* edges in a given BMG. To this end, we take a closer look at hourglass chains and the trees that explain them. In Fig. 6(D), each subtree $T(v_i)$, $1\le i \le k$, “bridges” the color sets of the subtrees $T(v_{i-1})$ and $T(v_{i+1})$. That is, $\sigma (L(T(v_{i-1})))\cap \sigma (L(T(v_i)))$ and $\sigma (L(T(v_i)))\cap \sigma (L(T(v_{i+1})))$ are non-empty. This suggests to consider the children of a vertex *u* as the vertices of a “color-set intersection graph” with edges connecting children with non-empty color-set intersection:

#### Definition 7

The *color-set intersection graph* ${\mathfrak {C}}_T(u)$ of an inner vertex *u* of a leaf-colored gene tree $(T,\sigma )$ is the undirected graph with vertex set $V:=\mathsf {child}_T(u)$ and edge set

$$\begin{aligned} E:=\{ v_1v_2 \mid v_1,v_2\in V \text {, }v_1\ne v_2 \text { and } \sigma (L(T(v_1)))\cap \sigma (L(T(v_2)))\ne \emptyset \}. \end{aligned}$$

This construction is similar to the definition of intersection graphs e.g. used in McKee and McMorris (1999). ${\mathfrak {C}}_T(u)$ can be viewed as a natural generalization of ${\mathcal {S}}^{\cap }(x,y)$ in the following sense: if $u={{\,\mathrm{lca}\,}}_T(x,y)$ is a binary vertex, then ${\mathfrak {C}}_T(u)=K_2$ *iff* ${\mathcal {S}}^{\cap }(x,y)\ne \emptyset$ and therefore, ${\mathfrak {C}}_T(u)=K_1\cup K_1$ *iff* ${\mathcal {S}}^{\cap }(x,y)=\emptyset$. In the non-binary case, there is an edge $v_1v_2$ *iff* ${\mathcal {S}}^{\cap }(x,y)\ne \emptyset$ for some $x\in L(T(v_1))$ and $y\in L(T(v_2))$.

Every BMG $(\vec {G},\sigma )$ contains all information necessary to determine the trees $(T,\sigma )$ by which it is explained. Since *u-fp* edges are defined in terms of the explaining trees, every BMG $(\vec {G},\sigma )$ also contains – at least implicitly – all information needed to identify its *u-fp* edges. Since $(\vec {G},\sigma )$ is determined by its unique least resolved tree $(T^*,\sigma )$, the *u-fp* edges must also be determined by $(T^*,\sigma )$. It is not sufficient for this purpose, however, to find an event labeling *t* of the vertices of $T^*$.

**Fig. 7:**
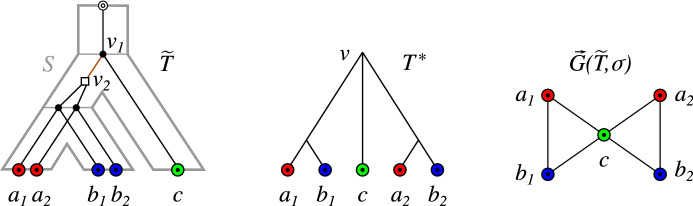
The evolutionary scenario (left) shows the event-labeled gene tree $({\widetilde{T}},{\widetilde{t}},\sigma )$ embedded into a species tree *S*. In the least resolved tree $(T^*,\sigma )$ of $\vec {G}({\widetilde{T}},\sigma )$, the edge $v_1v_2$ of ${\widetilde{T}}$ has been contracted into vertex *v*. The BMG $\vec {G}({\widetilde{T}},\sigma )$ does not contain any *u-fp* edge. *See text for further explanations*

To see this, consider for example the “true” history $({\widetilde{T}},{\widetilde{t}},\sigma )$ of the BMG $\vec {G}({\widetilde{T}},\sigma )$ as shown in Fig. 7. The unique least resolved tree $(T^*,\sigma )$ for $\vec {G}({\widetilde{T}},\sigma )$ is obtained by merging the two vertices $v_1$ and $v_2$ of ${\widetilde{T}}$ resulting in the vertex *v* of $T^*$. We have ${\widetilde{t}}(v_1)= \newmoon \ne \square ={\widetilde{t}}(v_2)$. For vertex *v* and every reconciliation map $\mu$ from $(T^*,\sigma )$ to any species tree *S*, it must hold that $\mu (v)\in E(S)$ and thus $t^*_{\mu }(v)=\square$, since *v* has two children with overlapping color sets and by Lemma 2. Thus, the edges *cx* with $x\in \{a_1,a_2,b_1,b_2\}$ are $(T^*,\sigma )$-*fp* although they are not false positives at all. Since speciation and duplication vertices may be merged into the same vertex *v* of $T^*$, the least resolved tree $T^*$ in general cannot simply inherit the event labeling from the true gene history, and thus there may not be a “correct” labeling $t^*$ of $T^*$ that provides evidence for all *u-fp* edges.

The example in Fig. 7 shows that the least resolved tree $T^*$ simply may not be “resolved enough”. In the following, we therefore describe how the unique least resolved tree can be resolved further to provide more evidence about *u-fp* edges. Eventually, this will lead us to a characterization of the *u-fp* edges. To this end, we need to gain more insights into the structure of redundant edges, i.e., those edges *e* in *T* for which $(T_e,\sigma )$ still explains $\vec {G}(T,\sigma )$.

Since the color sets of distinct subtrees below a speciation vertex cannot overlap by Lemma 2, Cor. 1 (Sec. A) implies that all edges below a speciation vertex are redundant and thus can be contracted. More precisely, we have

#### Observation 8

Let $\mu$ be a reconciliation map from $(T,\sigma )$ to *S* and assume that there is a vertex $u\in V^0(T)$ such that $\mu (u)\in V^0(S)$ and thus, $t_{\mu }(u)=\newmoon$. Then every inner edge *uv* of *T* with $v\in \mathsf {child}_{T}(u)$ is redundant w.r.t. $\vec {G}(T,\sigma )$. Moreover, if an inner edge *uv* with $v\in \mathsf {child}_{T}(u)$ is non-redundant, then *u* must have two children with overlapping color sets, and hence, $t_{\mu }(u)=\square$.

Our goal is to identify those vertices in $(T^*,\sigma )$ that can be expanded to yield a tree that still explains $\vec {G}(T^*,\sigma )$. To this end, we need to introduce a particular way of “augmenting” a leaf-colored tree.

#### Definition 18

Let $(T,\sigma )$ be a leaf-colored tree, *u* be an inner vertex of *T*, ${\mathfrak {C}}_T(u)$ the corresponding color-set intersection graph, and ${\mathcal {C}}$ the set of connected components of ${\mathfrak {C}}_T(u)$. Then the tree $T_u$ *augmented at vertex* *u* is obtained by applying the following editing steps to *T*:

- If ${\mathfrak {C}}_T(u)$ is connected, do nothing.
- Otherwise, for each $C\in {\mathcal {C}}$ with $|C|>1$
  - introduce a vertex *w* and attach it as a child of *u*, i.e., add the edge *uw*,
  - for every element $v_i\in C$, substitute the edge $uv_i$ by the edge $wv_i$.

The augmentation step is *trivial* if $T_u=T$, in which case we say that *no edit step was performed*.

**Fig. 8:**
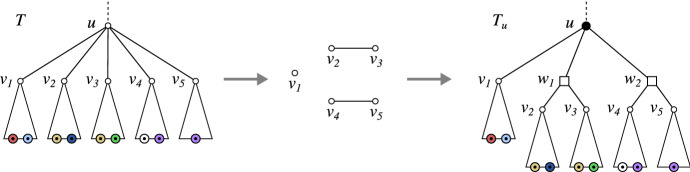
Left, a (part of a) leaf-colored tree $(T,\sigma )$. The tree $(T_u,\sigma )$ on the right is obtained from $(T,\sigma )$ by augmenting *T* at vertex *u*. The color-set intersection graph ${\mathfrak {C}}_T(u)$ (shown in the middle) has more than one connected component and there are connected components consisting of more than two vertices $v_i\in \mathsf {child}_T(u)$. According to Lemma 21, $\sigma (L(T_u(v)))\cap \sigma (L(T_u(v')))=\emptyset$ for any two distinct vertices $v,v'\in \mathsf {child}_{T_u}(u) = \{v_1,w_1,w_2\}$. By Cor. 1 (Sec. A), the edges $uw_1$ and $uw_2$ are redundant w.r.t. $\vec {G}(T_u,\sigma )$ and thus, both trees explain the same BMG

An example of an augmentation is shown in Fig. 8. The tree $T_u$ obtained by an augmentation of a phylogenetic tree *T* is again a phylogenetic tree.

A key property of the procedure in Def. 18 is that repeated augmentation of the same inner vertex leads to at most one expansion and that the order of augmenting multiple vertices does not matter. More precisely, Lemma 23 in Sec. C.3 ensures the existence of a unique augmented tree:

#### Definition 19

*(Augmented tree)* Let $(T,\sigma )$ be a leaf-colored tree. The *augmented tree of* $(T,\sigma )$, denoted by $({{\,\mathrm{{\mathcal {A}}}\,}}(T),\sigma )$, is obtained by augmenting all inner vertices of $(T,\sigma )$ (in an arbitrary order).

In particular, the augmented tree preserves the best match relation:

#### Proposition 7

For every leaf-colored tree $(T,\sigma )$, it holds $\vec {G}(T,\sigma )=\vec {G}({{\,\mathrm{{\mathcal {A}}}\,}}(T),\sigma )$.

We now have everything in place to present the main results of this section.

#### Theorem 10

Let $(\vec {G},\sigma )$ be a BMG, $(T^*,\sigma )$ its unique least resolved tree, and ${{\widehat{t}}}:={{\widehat{t}}_{{{\,\mathrm{{\mathcal {A}}}\,}}(T^*)}}$ the extremal event labeling of the augmented tree $({{\,\mathrm{{\mathcal {A}}}\,}}(T^*),\sigma )$. Then $(\Theta ({{\,\mathrm{{\mathcal {A}}}\,}}(T^*),{{\widehat{t}}}),\sigma ) = {\mathbb {N}}{\mathbb {H}}(\vec {G},\sigma )$.

Since $(\Theta ({{\,\mathrm{{\mathcal {A}}}\,}}(T^*),{{\widehat{t}}}),\sigma ) = {\mathbb {N}}{\mathbb {H}}(\vec {G},\sigma )$ is the subgraph of the underlying RBMG of $(\vec {G},\sigma )$ that does not contain any *u-fp* edges (cf. Def. 17 and Thm. 11), the set of all *u-fp* edges can readily be obtained by comparing the edges of $(\vec {G},\sigma )$ with the edges in the orthology graph obtained from $({{\,\mathrm{{\mathcal {A}}}\,}}(T^*),{{\widehat{t}}})$. Since only *u-fp* edges have been removed to obtain $(\Theta ({{\,\mathrm{{\mathcal {A}}}\,}}(T^*),{{\widehat{t}}}),\sigma )$ and since $({{\,\mathrm{{\mathcal {A}}}\,}}(T^*),\sigma )$ still explains $(\vec {G},\sigma )$, the graph $(\Theta ({{\,\mathrm{{\mathcal {A}}}\,}}(T^*),{{\widehat{t}}}),\sigma )$ is, in the sense of an unambiguous editing, the best estimate of the orthology relation that we can make by solely utilizing the structural information of a given BMG $(\vec {G},\sigma )$. Note, Thm. 1 implies that ${\mathbb {N}}{\mathbb {H}}(\vec {G},\sigma )$ must, in particular, be a cograph.

Since $(\Theta ({{\,\mathrm{{\mathcal {A}}}\,}}(T^*),{{\widehat{t}}}),\sigma ) = {\mathbb {N}}{\mathbb {H}}(\vec {G},\sigma )$, the computation of ${\mathbb {N}}{\mathbb {H}}(\vec {G},\sigma )$ can be achieved in polynomial time and avoids the need to find the hourglass chains of $(\vec {G},\sigma )$. In fact, the effort is dominated by computing the least resolved tree $(T^*,\sigma )$ for a given BMG.

#### Theorem 12

For a given BMG $(\vec {G},\sigma )$, the set of all *u-fp* edges can be computed in $O(|L|^3 |{\mathscr {S}}|)$ time, where $L=V(\vec {G})$ and ${\mathscr {S}} = \sigma (L(T))$ is the set of species under consideration.

As argued in (Geiß et al. 2019, Sec. 5), the number of genes between different species will be comparable in practical applications, i.e., $O(\ell ) = O(|L|/|{\mathscr {S}}|)$ with $\ell = \max _{s\in {\mathscr {S}}} |L[s]|$. In this case, the running time to compute $(T^*,\sigma )$ reduces to $O(|L|^3/|{\mathscr {S}}|)$ and we obtain an overall running time to compute the set of all *u-fp* edges of $O(|L|^3/|{\mathscr {S}}| + |L|^2 |{\mathscr {S}}|)$. Thms. 10 and 12 imply that we do not need to find induced quartets and hourglasses explicitly, nor do we need to identify the hourglass chains. Instead, it is more efficient to compute the least resolved tree $(T^*,\sigma )$, its augmented tree $({{\,\mathrm{{\mathcal {A}}}\,}}(T^*),\sigma )$, and the corresponding extremal event labeling ${{\widehat{t}}}$.

**Fig. 9:**
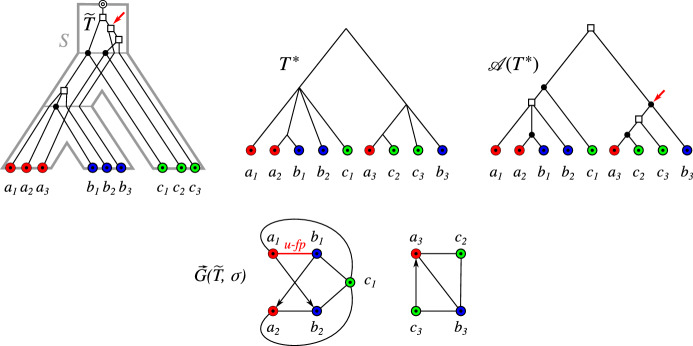
An evolutionary scenario (left) with a no-hug graph ${\mathbb {N}}{\mathbb {H}}(\vec {G},\sigma )$ that still contains false-positive edges. Deletion of the highlighted *u-fp* edge $a_1b_1$ for $\vec {G}({\widetilde{T}},\sigma )$ yields ${\mathbb {N}}{\mathbb {H}}(\vec {G},\sigma ) = (\Theta ({{\,\mathrm{{\mathcal {A}}}\,}}(T^*),{{\widehat{t}}}),\sigma )$ and thus, an orthology graph. However, none of its cotrees can be reconciled with any species tree since each of them contains the contradictory species triples $\sigma (a_1)\sigma (b_1)|\sigma (c_1)$ and $\sigma (a_1)\sigma (c_1)|\sigma (b_1)$ (see e.g. Hernandez-Rosales et al. (2012), Hellmuth (2017)). Note, the trees $({\widetilde{T}},{\widetilde{t}})$ and $({{\,\mathrm{{\mathcal {A}}}\,}}(T^*),{{\widehat{t}}})$ differ in the event label marked by the arrows, resulting in the three additional *fp* edges $a_3b_3$, $c_2b_3$ and $c_3b_3$ in ${\mathbb {N}}{\mathbb {H}}(\vec {G},\sigma )$

Deletion of all *u-fp* edges is necessary to obtain an orthology relation without false positives. It is not sufficient, however, since ${\mathbb {N}}{\mathbb {H}}(\vec {G},\sigma )$ may contain additional false-positive orthology assignments. In order to construct an example, we consider for a BMG $(\vec {G},\sigma )$ the set ${\mathfrak {T}}$ of all trees $(T,t,\sigma )$ for which ${\mathbb {N}}{\mathbb {H}}(\vec {G},\sigma ) = (\Theta (T,t),\sigma )$. The example in Fig. 9 shows that it may be the case that none of the trees $(T,t,\sigma )\in {\mathfrak {T}}$ admits a reconciliation map $\mu$ to any species tree such that $t_{\mu } = t$. Lemma 29 in Sec. C.5 shows that the augmented tree $({{\,\mathrm{{\mathcal {A}}}\,}}(T^*),{{\widehat{t}}},\sigma )$ is sufficient to test in polynomial time whether or not ${\mathfrak {T}}$ contains a reconcilable tree. In the negative case, we have clear evidence that ${\mathbb {N}}{\mathbb {H}}(\vec {G},\sigma )$ still contains a false-positive edge and thus must be edited further. This type of false-positive orthology assignments is the topic of ongoing work.

In contrast to the LRT of a BMG, its augmented tree is not necessarily displayed by the true gene tree of the underlying evolutionary scenario. Hence, we advocate the augmented tree endowed with the corresponding extremal event labeling $({{\,\mathrm{{\mathcal {A}}}\,}}(T^*),{{\widehat{t}}},\sigma )$ primarily as convenient tool to identify false-positive orthology assignments. Whether or not $({{\,\mathrm{{\mathcal {A}}}\,}}(T^*),{{\widehat{t}}},\sigma )$ is a plausible representation of the gene phylogeny depends on whether it admits a reconciliation of the (phylogenetically correct) species tree. As discussed above, this is not always the case. The following result, however, shows that $({{\,\mathrm{{\mathcal {A}}}\,}}(T^*),{{\widehat{t}}},\sigma )$ is informative in an important special case.

#### Lemma 30

Let $(T,t,\sigma )$ be an event-labeled tree explaining the BMG $(\vec {G},\sigma )$, and let $(T^*,\sigma )$ be the least resolved tree of $(\vec {G},\sigma )$. If $(\Theta (T,t),\sigma ) = {\mathbb {N}}{\mathbb {H}}(\vec {G},\sigma )$, then ${{\,\mathrm{{\mathcal {A}}}\,}}(T^*)$ is displayed by *T*.

Lemma 30 guarantees that ${{\,\mathrm{{\mathcal {A}}}\,}}(T^*)$ is displayed by the true gene tree ${\widetilde{T}}$ whenever ${\mathbb {N}}{\mathbb {H}}(\vec {G},\sigma )$ equals the true orthology relation. In a practical workflow, it can be checked efficiently whether there is evidence for additional false-positive edges because ${\mathfrak {T}}$ contains no reconcilable tree. If this is not the case, then it is likely that ${\mathbb {N}}{\mathbb {H}}(\vec {G},\sigma )$ equals the true orthology relation. In this case, ${\widetilde{T}}$ also displays the unique discriminating cotree of ${\mathbb {N}}{\mathbb {H}}(\vec {G},\sigma )$.

One has to keep in mind, however, that it is not possible to find a mathematical guarantee for ${\mathbb {N}}{\mathbb {H}}(\vec {G},\sigma )$ to be the true orthology relation, because it cannot be ruled out that the true scenario contains unwitnessed duplications that are compensated by additional gene losses. In the extreme case, it is logically possible for every BMG that, in the true scenario, all inner vertices of the gene tree predate the root of the species tree, resulting in a true orthology graph without any edges Guigó et al. (1996), Page and Charleston (1997), Geiß et al. (2020b). Of course, this is extremely unlikely for real data.

### Quartets, hourglasses, and the structure of reciprocal best match graphs

The characterization of *u-fp* edges is in a way surprising when compared to previous results on the structure of RBMGs Geiß et al. (2020b, 2020c), which were focused on $P_4$s and quartets. The expected connection between good and ugly quartets and *u-fp* edges is captured by Cor. 4. However, Prop. 6 implies that there are also *u-fp* edges entirely unrelated to quartets and thus induced $P_4$s. In this section, we aim to close this gap in our understanding.

*Hourglass-free BMGs.* We start with an important special case for which quartets are sufficient.

#### Definition 20

A BMG $(\vec {G},\sigma )$ is *hourglass-free* if it does not contain an hourglass as an induced subgraph.

In particular, an hourglass-free BMG does not contain an hourglass chain. It turns out that hourglasses are the forbidden induced subgraph characterizing BMGs that can be explained by binary trees.

#### Prop. 8 and Cor. 6

A BMG $(\vec {G},\sigma )$ can be explained by a binary tree if and only if it is hourglass-free. In particular, it can be decided in polynomial time whether $(\vec {G},\sigma )$ can be explained by a binary tree.

The RBMGs that are already cographs are called *co-RBMGs*. As shown in Sec. D.1, we obtain

#### Corollary 7

Let $(\vec {G},\sigma )$ be an hourglass-free BMG. Then its symmetric part $(G,\sigma )$ is either a co-RBMG or it contains an induced $P_4$ on three colors whose endpoints have the same color, but no induced cycle $C_n$ on $n\ge 5$ vertices.

As outlined in Sec. D.1, all *u-fp* edges in an hourglass-free BMG are identified by the good and ugly quartets, which are 3-colored by construction. In hourglass-free BMGs, it is indeed sufficient to consider only the 3-colored $P_4$s to identify all *u-fp* edges and thus, to obtain an orthology graph, even though the BMG may also contain 4-colored $P_4$s. Since hourglasses can only appear in BMGs that require multifurcations for their explanation (cf. Lemma 14), the case of hourglass-free BMGs is the most relevant for practical applications.

Since all *u-fp* edges in an hourglass-free BMG are contained in quartets, it is also easy to identify the hourglass-free BMGs that are already orthology graphs.

#### Corollary 8

Let $(\vec {G},\sigma )$ be an hourglass-free BMG. Then, its symmetric part $(G,\sigma )$ is a co-RBMG if and only if there are no *u-fp* edges in $(\vec {G},\sigma )$.

*u-fp* *Edges in Hourglass Chains.* The situation is much more complicated in the presence of hourglasses. We start by providing sufficient conditions for *u-fp* edges that are identified by hourglass chains.

#### Proposition 9

Let  be an hourglass chain in $(\vec {G},\sigma )$, possibly with a left tail *z* or a right tail $z'$. Then, an edge in $\vec {G}$ is *u-fp* if it is contained in the set

$$\begin{aligned} F =&\{x_iy_j\mid 1\le i \le j \le k\} \cup \{zz'\} \cup \{zy_{i}, x_iz', zy'_{i}, x'_{i}z' \mid 1 \le i \le k \}\\&\cup \{ x_{i}x_{j+1} \mid 1\le i< j< k \} \cup \{ y_{i}y_{j+1} \mid 1\le i< j < k \} \\&\cup \{x'_1 y'_i, x'_1 y_i \mid 2 \le i \le k \} \cup \{x_i y'_k, x'_i y'_k \mid 1 \le i \le k-1 \} \\&\cup \{x'_1 z, x'_1 z', y'_k z, y'_k z'\} \end{aligned}$$

As outlined in Sec. D.2, hourglass chains identify false-positive edges that are not associated with quartets in the BMG and, in particular, false-positive edges that are not even part of an induced $P_4$. This observation limits the use of cograph editing in the context of orthology detection, at least in the case of gene trees with polytomies: On one hand, an RBMG can be a cograph and still contain *u-fp* edges and, on the other hand, there are examples where deletion of the *u-fp* edge identified by quartets (and thus, by induced $P_4$s) is not sufficient to arrive at a cograph (cf. Sec. D.2).

*Four-colored* $P_4$*s* Geiß et al (2020c, Thm. 8) established that the RBMG $(G,\sigma )$ is a co-RBMG, i.e., a cograph, if and only if every subgraph induced on three colors is a cograph. Therefore, if $(G,\sigma )$ contains an induced 4-colored $P_4$, it also contains an induced 3-colored $P_4$. For hourglass-free BMGs $(\vec {G},\sigma )$ it is clear that a 4-colored $P_4$ always overlaps with a 3-colored $P_4$: In this case ${\mathbb {N}}{\mathbb {H}}(\vec {G},\sigma )$ is obtained by deleting middle edges of good quartets and first edges of ugly quartets. Since ${\mathbb {N}}{\mathbb {H}}(\vec {G},\sigma )$ is a cograph, there is no $P_4$ left, and thus at least one edge of any 4-colored $P_4$ was among the deleted edges. It is natural to ask whether this is true for BMGs in general. However, as shown in Sec. D.3, good and ugly quartets are not sufficient on their own and there are examples with 4-colored $P_4$s that do not overlap with the middle edge of a good quartet or the first edge of an ugly quartet.

Still, in the context of cograph-editing approaches it is of interest whether the 3-colored $P_4$s are sufficient. In the following we provide an affirmative answer.

#### Lemma 34

Let $(\vec {G},\sigma )$ be a BMG and ${\mathscr {P}}$ a 4-colored induced $P_4$ in the symmetric part of $(\vec {G},\sigma )$. Then at least one of the edges of ${\mathscr {P}}$ is either the middle edge of some good quartet or the first edge of a bad or ugly quartet in $(\vec {G},\sigma )$.

It is important to recall in this context, however, that the deletion of all *u-fp*-edges identified by quartets does not necessarily lead to a cograph (see Fig. 17(C) in Sec. D.3 for an example). Hence, the quartets alone therefore cannot provide a complete algorithm for correcting an RBMG to an orthology graph.

## Simulation results

We illustrate the potential impact of our mathematical results discussed in the previous sections with the help of simulated data. To this end, we focus on the accuracy of the inferred orthology graph *assuming* that the best matches are accurate. Of course, this is only one of several components in complete orthology detection pipeline, which would also need to consider the genome annotation, pairwise alignments of genes or predicted protein sequences, and the conversion of sequence similarities into best match data. The latter step has been investigated in considerable detail by Stadler et al. (2020). Here, we start from simulated evolutionary scenarios and extract the BMG directly from the ground truth using the simulation library AsymmeTreeStadler et al. (2020).

In brief, AsymmeTree generates realistic evolutionary scenarios in four steps. (1) A planted species tree *S* is generated using the Innovation Model Keller-Schmidt and Klemm (2012), which models observed phylogenies well. (2) A dating map $\tau$ assigns time points to all vertices of *S* and thus branch lengths to the edges of *S*. (3) On *S*, we use a variant of the well-known constant-rate birth-death process with a given age (see e.g. Kendall 1948; Hagen and Stadler 2018) to simulate an event-labeled gene tree $(T,t,\sigma )$ containing duplication and loss events. Speciations are included as additional branching events that generate copies of all genes present at a speciation vertex in all descendant lineages. The simulated gene trees are constrained to have at least one surviving gene in each species to avoid trivial cases. (4) The observable part of the gene tree is extracted by recursively removing leaves that correspond to loss events and suppressing inner vertices with a single child. AsymmeTree can also assign rates to edges of $(T,t,\sigma )$ to convert evolutionary time differences into general additive distances; however, this is not relevant here since the rates do not affect evolutionary relatedness and thus the BMG.

**Fig. 10:**
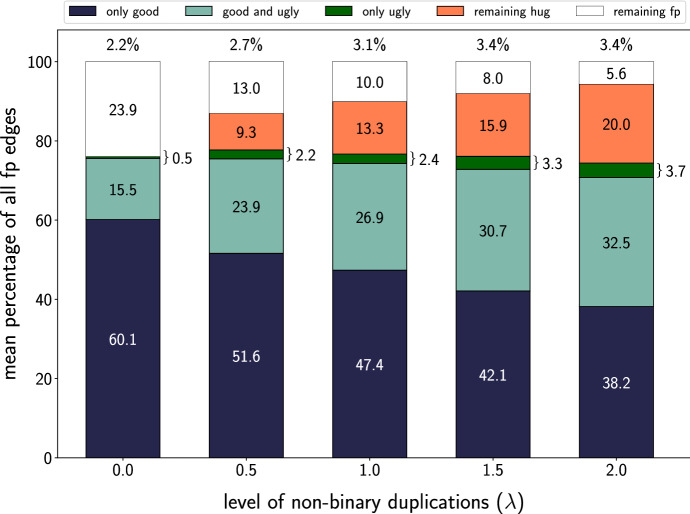
Average relative abundance of the different types of hug-edges and undetectable false positives in the BMGs of simulated evolutionary scenarios. We distinguish hug-edges in good and ugly quartets as well as hug-edges appearing only in hourglass chains (orange). In the simulations, the fraction of *u-fp* edges that are first edges of bad quartets is too small too be visible and therefore not shown here. The undetectable false positives correspond to complementary gene losses without surviving witnesses of the duplication event. Species trees are binary, while gene trees contain multifurcations. The number of offsprings is modeled as $2+k$, where *k* is drawn from a Poisson distribution with parameter $\lambda$. For $\lambda =0$, the gene trees are binary. In the experiments, we observed that on average 62.4% of the 25000 simulated BMGs do not contain any false-positive edge (cf. Fig. 11). Those instances are included in the computation of the fraction $|{\mathfrak {F}}|/|E(G)|$ (percentage above the bars). However, for the computation of all other values only scenarios that contain false-positives are considered

Extending the simulations used in Geiß et al. (2020b), Stadler et al. (2020), we also consider non-binary gene trees. This is important here since, by Lemma 14, hourglasses cannot appear in BMGs that are explained by a binary tree. There is an ongoing discussion to what extent polytomies in phylogenetic trees are biological reality as opposed to an artifact of insufficient resolution. At the level of species trees, the assumption that cladogenesis occurs by a series of bifurcations (e.g. Maddison 1989; DeSalle et al. 1994) seems to be prevailing, several authors have argued quite convincingly that there is evidence for a least some *bona fide* multifurcations of species Kliman et al. (2000), Takahashi et al. (2001), Sayyari and Mirarab (2018). In the simulation, polytomies in species trees are introduced after the first step by edge contraction with a user-defined probability *p*.

The reality of polytomies is less clear for gene trees. One reason is the abundance of tandem duplications. Although the majority of tandem arrays comprises only a pair of genes, larger clusters are not at all rare Pan and Zhang (2008). Although one may argue that mechanistically they likely arise by stepwise duplications, such arrangements are often subject to gene conversion and non-homologous recombination that keeps the sequences nearly identical for some time before they eventually escape from concerted evolution and diverge functionally Liao (1999), Hanada et al. (2018). As a consequence, duplications in tandem arrays may not be resolvable unless witnesses of different stages of an ongoing duplication process have survived. To model polytomies in the gene tree, we modify step (3) of the simulation procedure by replacing a simple duplication by the generation of $2+k$ offspring genes. The number *k* of additional copies is drawn from a Poisson distribution with parameter $\lambda >0$.

The simulated data set of evolutionary scenarios comprises species trees with 10 to 30 species (drawn uniformly). The time difference between the planted root and the leaves of *S* is set to unity. The duplication and loss rates in the gene trees are drawn i.i.d. from the uniform distribution on the interval [0.5, 1.5). Multifurcating gene trees were produced for $\lambda =\{0.0, 0.5, 1.0, 1.5, 2.0\}$. In total, we generated 5000 scenarios for each choice of *p* and $\lambda$. Since the true scenarios, and thus the true gene tree *T*, the true BMG $\vec {G}$, and the corresponding RBMG *G* are known, we can also determine the set

1 $$\begin{aligned} {\mathfrak {F}}:=\left\{ xy \;\mid \; xy\in E(G) \;\;\text {and}\;\; t({{\,\mathrm{lca}\,}}_T(x,y))=\square \right\} . \ \end{aligned}$$

of false-positive edges. From the BMG, we compute the set ${\mathfrak {U}}$ of *u-fp* edges as well as the subsets ${\mathfrak {U}}_M$ and ${\mathfrak {U}}_U$ of *u-fp* edges that are middle edges of a good or first edges of an ugly quartet, respectively. Note that in general we have ${\mathfrak {U}}_M\cap {\mathfrak {U}}_U\ne \emptyset$. We only discuss the results for binary species trees in some detail, since species trees with polytomies yield qualitatively similar results. We observe that the relative abundance of *u-fp* edges in good and ugly quartets increases moderately for larger *p*.

**Fig. 11:**
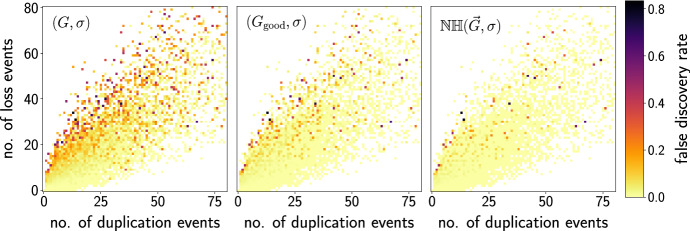
False discovery rates computed as proportion of *fp* among all edges averaged over all scenarios with given number of duplications and losses. *Left:* RBMGs $(G,\sigma )$, i.e., $|{\mathfrak {F}}|/|E(G)|$. *Middle:* edited RBMG $(G_\text {good},\sigma )$ with all middle edges of good quartets removed, i.e., $|{\mathfrak {F}}{\setminus }{\mathfrak {U}}_M|/|E(G_\text {good})|$. *Right:* no-hug graphs ${\mathbb {N}}{\mathbb {H}}(\vec {G},\sigma )$, i.e., $|{\mathfrak {F}}{\setminus }{\mathfrak {U}}|/|E({\mathbb {N}}{\mathbb {H}})|$. Scenarios with more than 80 duplication/loss events are not shown

First, we note that, consistent with Geiß et al. (2020b), Stadler et al. (2020), the fraction $|{\mathfrak {F}}|/|E(G)|$ of false positive orthology assignments is small in our data set, on the order of $3\%$. This indicates that, in real-life data, the main source of errors is likely the accurate determination of best matches from sequence data rather than false-positive edges contained in the BMG. Considering the fraction $|{\mathfrak {U}}|/|{\mathfrak {F}}|$ of *u-fp* edges in Fig. 10, we find that even in the most adverse case of all gene trees being binary, the BMG identifies more than three quarters of ${\mathfrak {F}}$. It may be surprising at first glance that the problem becomes easier with increasing $\lambda$ and barely $6\%$ of the false positives escape discovery. A likely explanation is that multifurcations increase the likelihood that an inner vertex has two surviving lineages that serve as witnesses of the event; in addition, multifurcations increase the vertex degree in the BMG, so that in principle more information is available to resolve the tree structure. It is also interesting to note that ${\mathfrak {U}}_U{\setminus } {\mathfrak {U}}_M$ is small, i.e., there are few cases of first edges in an ugly quartet that are not also middle edges in a good quartet. The fraction of *u-fp* edges that appear only as first edges of bad quartets is even smaller; only 2-3% of the *u-fp* edges associated with hourglass chains, i.e., less than 0.15% of all *u-fp* edges are of this type. The overwhelming majority of *u-fp* edges associated with quartets thus appear (also) as middle edges of good quartets. This observation provides an explanation for the excellent performance of removing the ${\mathfrak {U}}_M$-edges proposed in Geiß et al. (2020b). In particular in the case of binary trees, which was considered by Geiß et al. (2020b), there is only a small number of other *u-fp* edges, which are completely covered by ${\mathfrak {U}}_U$. Fig. 11 visualizes the appearance of false-positive edges depending on the number of duplication and loss events. Not surprisingly, ${\mathfrak {F}}$ is enriched in scenarios with a large number of losses compared to the duplications, and depleted when losses are rare. In fact, in the absence of losses, the RBMG equals the orthology graph, i.e., ${\mathfrak {F}}=\emptyset$ (Geiß et al. 2020b, Thm. 4). Removal of ${\mathfrak {U}}_M$, already reduced the false positives considerably.

## Summary and outlook

We have shown here how all unambiguously false-positive orthology assignments can be identified in polynomial time provided that all best matches are known. In particular, we have provided several characterizations for *u-fp* edges in terms of underlying subgraphs and refinements of trees. Since the best match graph contains only false positives, we have obtained a characterization of *all* unambiguously incorrect orthology assignments. Simulations showed that the majority of false positives comprises middle edges of good quartets, while *u-fp* edges that appear only as first edges of an ugly quartet are rare. Not surprisingly, the hourglass-related *u-fp* edges become important in gene trees with many multifurcations. They do not appear in scenarios derived from binary gene trees. For the theory developed here, it makes no difference whether polytomies in the gene tree appear as genuine features, or whether limited accuracy of the approximation from underlying sequence data produced the equivalent of a soft polytomy in the BMG.

The augmented tree $({{\,\mathrm{{\mathcal {A}}}\,}}(T^*),\sigma )$ is the least resolved tree that admits an event labeling such that all inner vertices with child trees that have overlapping colors are designated as duplications while all inner vertices with color-disjoint child trees are designated as speciations. The tree $({{\,\mathrm{{\mathcal {A}}}\,}}(T^*),\sigma )$ therefore does not contain “non-apparent duplications” in the sense of Lafond et al. (2014), i.e., duplication vertices with species-disjoint subtrees. This is an interesting connection linking the literature concerned with polytomy refinement in given gene trees Chang and Eulenstein (2006), Lafond et al. (2014) with best match graphs.

The extremal event labeling ${{\widehat{t}}}$ of $({{\,\mathrm{{\mathcal {A}}}\,}}(T^*),\sigma )$ is the one that minimizes the necessary number of duplications on $({{\,\mathrm{{\mathcal {A}}}\,}}(T^*),\sigma )$. In a conceptual sense, therefore, $({{\,\mathrm{{\mathcal {A}}}\,}}(T^*),{{\widehat{t}}})$ is a “most parsimonious” solution, matching the idea of most parsimonious reconciliations Guigó et al. (1996), Page and Charleston (1997). From a technical point of view, however, the problem we solve here is very different. Instead of considering a given pair of gene tree *T* and species tree *S*, we ask here about the information contained in the BMG $(\vec {G},\sigma )$, i.e., we only consider the information on the species tree that is already implicitly contained in $(\vec {G},\sigma )$. The construction of the event-labeled gene tree $({{\,\mathrm{{\mathcal {A}}}\,}}(T^*),{{\widehat{t}}})$ in fact *implies* a set ${\mathfrak {S}}$ of informative triples, namely those $\sigma (x)\sigma (y)|\sigma (z)$ with $\sigma (x)$, $\sigma (y)$, $\sigma (z)$ pairwise distinct and ${{\widehat{t}}}({{\,\mathrm{lca}\,}}_{{{\,\mathrm{{\mathcal {A}}}\,}}(T^*)}(x,y,z))=\newmoon$, that are displayed by the species tree *S* Hernandez-Rosales et al. (2012), Hellmuth (2017). Nothing in our theory, however, ensures that ${\mathfrak {S}}$ is a consistent set of triples, much less that ${\mathfrak {S}}$ is consistent with a given species tree *S*. A lack of consistency, however, implies that the no-hug graph ${\mathbb {N}}{\mathbb {H}}(\vec {G},\sigma )$ cannot be the correct orthology relation, and thus, necessarily contains additional false-positive edges. Consistency, on the other hand, cannot provide a mathematical proof for biological correctness. It makes ${\mathbb {N}}{\mathbb {H}}(\vec {G},\sigma )$ a very likely candidate for the true orthology relation, however, because alternative scenarios require additional gene duplications and multiple, strategically placed gene losses to compensate for them.

Since constraints on reconciliation maps deriving from the species phylogeny are fully expressed by informative triples, no such constraint exists in particular for any vertex *u* of ${{\,\mathrm{{\mathcal {A}}}\,}}(T^*)$ that has only leaves as children. That is, false-positive orthology assignments among the children of *u* cannot be identified from the BMG alone because there are no further descendants to witness *u* as duplication event. Additional evidence, such as the assumption of a molecular clock or synteny must be used to resolve situations such as the complementary loss shown in Fig. 2.

Every gene tree *T* can be reconciled with every species tree *S*Guigó et al. (1996), Page and Charleston (1997), Geiß et al. (2020b) at the expense of reassigning events as duplications. If ${{\,\mathrm{{\mathcal {A}}}\,}}(T^*)$ is already binary, consistency will require the relabeling of some speciation nodes as duplications. Can one characterize and efficiently compute the minimal relabelings? In the general case, a further refinement of ${{\,\mathrm{{\mathcal {A}}}\,}}(T^*)$ may be sufficient. Is a refinement of speciation nodes sufficient, or are there in general speciation nodes in $({{\,\mathrm{{\mathcal {A}}}\,}}(T^*),{{\widehat{t}}})$ that need to be refined into separate speciation and duplication events?

Since orthology graphs are cographs contained in the RBMG $(G,\sigma )$, it is of interest to compare the deletion of all *u-fp* edges in $(G,\sigma )$ with finding a (minimal) edge-deletion set to obtain a cograph. These two problems are clearly distinct: The simplest example is the BMG $(\vec {G},\sigma )$ in Fig. 6(A): its symmetric part *G* is already a cograph but $(\vec {G},\sigma )$ contains the hug-edge *xy*, which must be deleted. Despite its practical use Hellmuth et al. (2015), Lafond et al. (2016), this observation relegates cograph editing Liu et al. (2012), Hellmuth et al. (2020a), Tsur (2020) to the status of a heuristic approximation for the purpose of orthology detection.

For practical applications, one has to keep in mind that best matches are inferred from sequence similarity data. Despite efforts to convert best (blast) hits into evolutionary best matches in a systematic manner Stadler et al. (2020), estimated BMGs will contain errors, which in most cases will violate the definition of best match graphs. This begs the question how an empirical estimate of a BMG can be corrected to a closest “correct” BMG that (approximately) fits the data. Not surprisingly, BMG editing Schaller et al. (2020) and the analogous RBMG editing problem Hellmuth et al. (2020b) are NP-hard. Efficient, accurate heuristics are a topic of ongoing research.

Orthology prediction tools intended for large data sets often do not attempt to infer the orthology graph, but instead are content with summarizing the information as *clusters of orthologous groups* (COGs) in an empirically estimated RBMG Tatusov et al. (1997), Roth et al. (2008). Formally, this amounts to editing the BMG to a set of disjoint cliques. The example in Fig. 7 shows that this approach can destroy correct orthology information: the BMG $(\vec {G},\sigma )$ does not contain *u-fp* edges and thus, it is the closest orthology graph. However, $(\vec {G},\sigma )$ is not the disjoint union of cliques.

## Figures and Tables

**Fig. 12:**
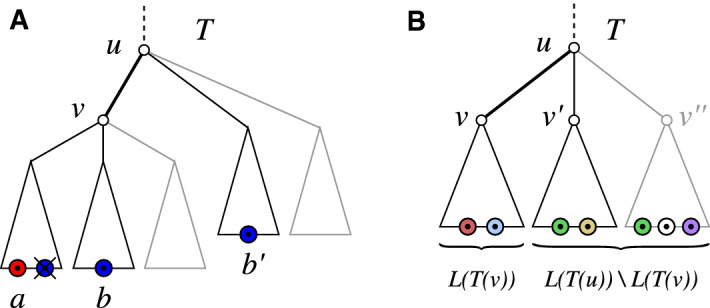
Visualization of (A) a *non-*redundant edge *uv* for Lemma 7 and (B) a redundant edge *uv* as in Cor. 1. The gray subtrees may or may not exist. In (A), the crossed out leaf indicates that the blue color must not be present in this subtree and thus (*a*, *b*) is a best match. In (B), $\sigma (L(T(v)))$ must not have elements in common with $\sigma (L(T(u)){\setminus } L(T(v)))$. See text for further details

**Fig. 13:**
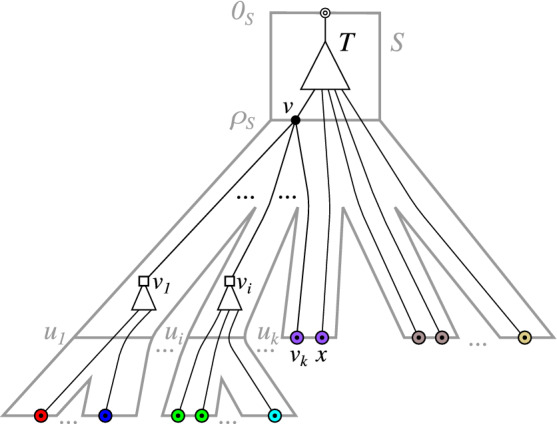
Visualization of the construction of a species tree *S* and reconciliation map $\mu$ as described in the proof of Lemma 10. Note that, in the example, $v_k$ is already a leaf in the gene tree *T*. Hence, the corresponding $u_k$ is also a leaf since $|\sigma (L(T(v_k)))|=1$. Moreover, note that for $x\in L(T){\setminus } L(T(v))$, it is possible that $\mu (x)=u_j$ or $\mu (x)=t$ with $t\in \mathsf {child}_{S}(u_j)$ for some $u_j$

**Fig. 14:**
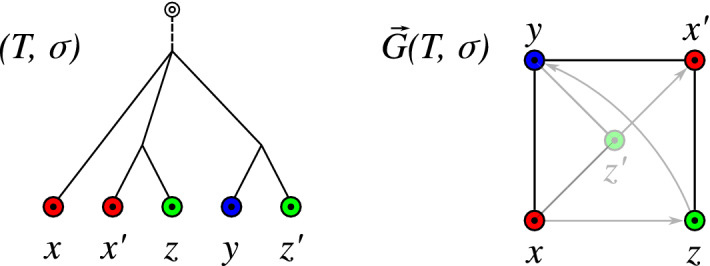
The edge *xy* is *u-fp* since it is the first edge of an ugly quartet. However, ${\mathcal {S}}^{\cap }(x,y)=\emptyset$ and thus, the converse of Prop. 5 is not satisfied

**Fig. 15:**
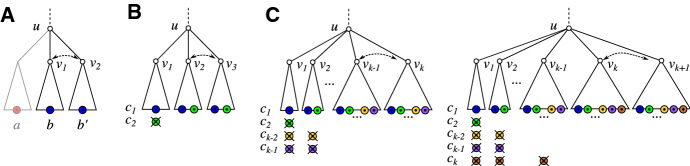
Illustration of the induction argument in the proof of Prop. 8. (A) Base case: an hourglass-free pair (hf-pair) $({\mathscr {M}}=\{v_1,v_2\},{\mathscr {N}}=\{\sigma (b)\})$ of order 2. Note that vertex *a* is only required to show the existence of $({\mathscr {M}},{\mathscr {N}})$. (B) An hf-pair $({\mathscr {M}}=\{v_1,v_2,v_3\},{\mathscr {N}}=\{c_1,c_2\})$ of order 3. (C) Induction step: The existence of an hf-pair of order *k* implies the existence of an hf-pair of order $k+1$, and thus, an infinite number of children of *u*. This gives the desired contradiction in the proof of Prop. 8. The dashed arrow indicates the last two elements in the ordered set ${\mathscr {M}}$ of an hf-pair $({\mathscr {M}},{\mathscr {N}})$ are interchangeable

**Fig. 16:**
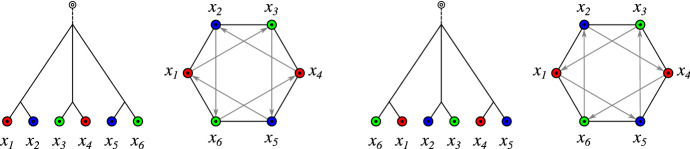
Two examples of trees whose BMGs $\vec {G}(T,\sigma )$ contain a hexagon $\langle x_1x_2x_3x_4x_5x_6\rangle$. There are exactly two distinct possibilities for the placement of the non-symmetric arcs in the subgraph of the BMG induced by the hexagon

**Fig. 17:**
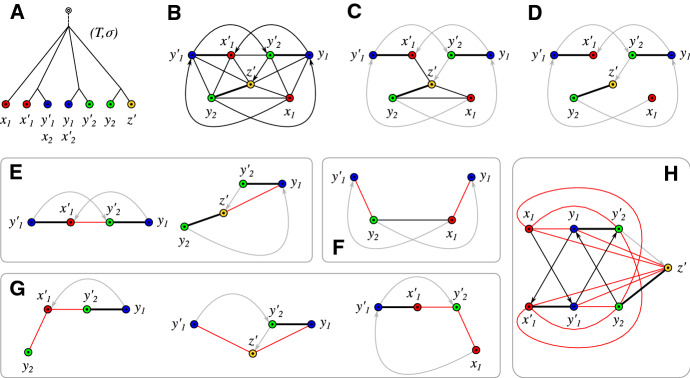
The (non-binary) tree $(T,\sigma )$ in Panel (A) explains the BMG $(\vec {G},\sigma )$ in Panel (B), which contains several induced $P_4$s and an hourglass chain of length $k=2$ with right tail $z'$. Edges that are not $(T,\sigma )$-*fp* (and thus not *u-fp*) are shown as thick lines. Thin edges correspond to those that can be identified as *u-fp* by the subgraphs in (E–H), where they are highlighted in red. (C) The graph after deletion of all edges that can be identified by good, bad and ugly quartets according to Props. 2, 3, and 4. Note that it contains the induced $P_4$s $\langle y_1'x_1'z'y_2\rangle$ and $\langle y_1'x_1'z'x_1\rangle$, which were not induced subgraphs of the original BMG in (B). Its symmetric part $(H,\sigma )$ differs from ${\mathbb {N}}{\mathbb {H}}(\vec {G},\sigma )$ (cf. Def. 17) since it still contains *u-fp* edges. (D) The BMG after deletion of all *u-fp* edges. Its symmetric part, comprising the thick edges, is ${\mathbb {N}}{\mathbb {H}}(\vec {G},\sigma )$. (E) The two good quartets. (F) The single bad quartet. (G) Examples for ugly quartets that cover the remaining *u-fp* edges that are identifiable via quartets. Panel (H) shows the BMG $(\vec {G},\sigma )$ in a different layout that highlights the hourglass chain with right tail $z'$. All edges that are *u-fp* according to Prop. 9 are in red. To identify the *u-fp* edges in $(\vec {G},\sigma )$, only the subgraphs in Panel (E), (G) and (H) are necessary (cf. Def. 16 and Thm. 10).

**Fig. 18:**
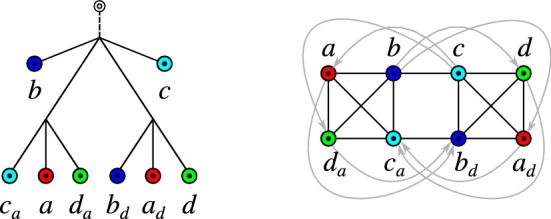
The symmetric part of the BMG $(\vec {G},\sigma )$ contains the 4-colored induced $P_4$ $\langle abcd \rangle$. None of its edges is the middle edge of a good quartet or the first edge of an ugly quartet. According to Lemma 34, there is the bad quartet $\langle abca_d \rangle$ that contains as first edge the edge *ab*

## (Reciprocal) best matches

We start by collecting some useful properties of BMGs and RBMGs that will be needed for later reference.

### Lemma 3

(Geiß et al. 2020c, Lemma 10) Let $(T,\sigma )$ be a leaf-colored tree on *L* and let $v\in V(T)$. Then, for any two distinct colors $r,s\in \sigma (L(T(v)))$, there is an edge *xy* in $\vec {G}(T,\sigma )$ with $x\in L[r]\cap L(T(v))$ and $y\in L[s]\cap L(T(v))$.

### Lemma 4

Let $(\vec {G},\sigma )$ be a BMG explained by a tree $(T,\sigma )$. Moreover, let $x,y \in L(T)$ with $\sigma (x)\ne \sigma (y)$ and $v_x,v_y\in \mathsf {child}({{\,\mathrm{lca}\,}}_T(x,y))$ with $x\preceq _Tv_x$ and $y\preceq _Tv_y$. Then, $\sigma (x)\notin \sigma (L(T(v_y)))$ and $\sigma (y)\notin \sigma (L(T(v_x)))$ if and only if *xy* is an edge in $\vec {G}$.

### Proof

By the definition of best matches, it holds that *xy* is an edge in $\vec {G}$ if and only if ${{\,\mathrm{lca}\,}}_T(x,y) \preceq _T {{\,\mathrm{lca}\,}}_T(x,y')$ for all $y'\in L(T)$ of color $\sigma (y)$ and ${{\,\mathrm{lca}\,}}_T(x,y) \preceq _T {{\,\mathrm{lca}\,}}_T(x',y)$ for all $x'\in L(T)$ of color $\sigma (x)$. Clearly, ${{\,\mathrm{lca}\,}}_T(x,y) \preceq _T {{\,\mathrm{lca}\,}}_T(x,y')$ for all such $y'$ if and only if $\sigma (y)\notin \sigma (L(T(v_x)))$, and ${{\,\mathrm{lca}\,}}_T(x,y) \preceq _T {{\,\mathrm{lca}\,}}_T(x',y)$ for all such $x'$ if and only if $\sigma (x)\notin \sigma (L(T(v_y)))$. $\square$

### Definition 8

Suppose that $(T,\sigma )$ explains $(\vec {G},\sigma )$. Then we say that $(T,\sigma )$ is *least resolved* (w.r.t. $(\vec {G},\sigma )$) if no tree $(T',\sigma )$ displayed by $(T,\sigma )$ explains $(\vec {G},\sigma )$.

Recall all trees in this contribution are planted, and thus least resolved trees (LRTs) are also considered as planted. Strictly speaking, this differs from the construction in Geiß et al. (2019, 2020c, 2020b), the additional (non-contractible) edge $0_T\rho _T$ is a trivial detail that does not affect the properties of LRTs.

### Theorem 3

(Geiß et al. 2019, Thm. 8 and Cor. 4) Every BMG $(\vec {G},\sigma )$ is explained by a unique least resolved tree $(T^*,\sigma )$. In particular, every other tree $(T,\sigma )$ explaining $(\vec {G},\sigma )$ is a refinement of $(T^*,\sigma )$. The least resolved tree $(T^* , \sigma )$ of a BMG $(\vec {G}, \sigma )$ can be constructed in polynomial time.

The following definition of informative triples is equivalent to the version given by Geiß et al. (2019).

### Definition 9

Let $(\vec {G},\sigma )$ be a colored digraph. We say that a triple $ab|b'$ is *informative* for $(\vec {G},\sigma )$ if *a*, *b* and $b'$ are three different vertices with $\sigma (a)\ne \sigma (b)=\sigma (b')$ in $\vec {G}$ such that $(a,b)\in E(\vec {G})$ and $(a,b')\notin E(\vec {G})$.

### Lemma 5

Let $(\vec {G},\sigma )$ be a BMG and $ab|b'$ an informative triple for $(\vec {G},\sigma )$. Then, every tree *T* that explains $(\vec {G},\sigma )$ displays the triple $ab|b'$, i.e. ${{\,\mathrm{lca}\,}}_T(a,b)\prec _T{{\,\mathrm{lca}\,}}_T(a,b')={{\,\mathrm{lca}\,}}_T(b,b')$.

### Proof

The definition of informative triples implies that $(a,b)\in E(\vec {G})$ and $(a,b')\notin E(\vec {G})$. Using $\sigma (b)=\sigma (b')$ and the definition of best matches we immediately conclude ${{\,\mathrm{lca}\,}}_T(a,b)\prec _T{{\,\mathrm{lca}\,}}_T(a,b')$. $\square$

### Lemma 6

Let $ab|b'$ and $cb'|b$ be informative triples for a BMG $(\vec {G},\sigma )$. Then every tree $(T,\sigma )$ that explains $(\vec {G},\sigma )$ contains two distinct children $v_1, v_2\in \mathsf {child}_{T}({{\,\mathrm{lca}\,}}_{T}(a,c))$ such that $a,b\prec _T v_1$ and $b',c\prec _T v_2$.

### Proof

Let $(T,\sigma )$ be an arbitrary tree that explains $(\vec {G},\sigma )$. By Lemma 5, *T* displays the informative triples $ab|b'$ and $cb'|b$. Thus we have ${{\,\mathrm{lca}\,}}_{T}(a,b)\prec _T{{\,\mathrm{lca}\,}}_{T}(a,b')={{\,\mathrm{lca}\,}}_{T}(b,b')$ and ${{\,\mathrm{lca}\,}}_{T}(c,b')\prec _T{{\,\mathrm{lca}\,}}_{T}(c,b)={{\,\mathrm{lca}\,}}_{T}(b,b')$. In particular, ${{\,\mathrm{lca}\,}}_{T}(a,b')={{\,\mathrm{lca}\,}}_{T}(b,b')={{\,\mathrm{lca}\,}}_{T}(c,b)=:u$. Therefore, $a\preceq _{T} v_1$ and $b'\preceq _{T} v_2$ for distinct $v_1, v_2\in \mathsf {child}_{T}(u)$. Since ${{\,\mathrm{lca}\,}}_{T}(a,b)\prec _T u$, we have $a,b\prec _T v_1$ and thus $v_1$ is an inner node. Likewise, ${{\,\mathrm{lca}\,}}_{T}(b',c)\prec _{T}u$ implies $b',c\prec _{T}v_2$. $\square$

Given a tree *T* and an edge *e*, denote by $T_e$ the tree obtained from *T* by contracting the edge *e*. An edge $e\ne 0_T\rho _T$ in $(T,\sigma )$ is *redundant (w.r.t.* $(\vec {G},\sigma )$*)* if $(T,\sigma )$ explains $(\vec {G},\sigma )$ and $\vec {G}(T_e,\sigma )=\vec {G}(T,\sigma )$. Redundant edges have already been characterized in (Geiß et al. 2019, Lemma 15, Thm. 8) in terms of equivalence classes using a more complicated notation. Here we give a simpler characterization:

### Lemma 7

Let $(\vec {G},\sigma )$ be a BMG explained by a tree $(T,\sigma )$. The edge $e=uv$ with $v\prec _T u$ in $(T,\sigma )$ is redundant w.r.t. $(\vec {G},\sigma )$ if and only if (i) *e* is an inner edge of *T* and (ii) there is no arc $(a,b)\in E(\vec {G})$ such that ${{\,\mathrm{lca}\,}}_T(a,b)=v$ and $\sigma (b)\in \sigma (L(T(u)){\setminus } L(T(v)))$.

### Proof

Let $w_e$ be the vertex in $T_e$ resulting from the contraction $e=uv$ with $v\prec _T u$ in *T*. By assumption we have $(\vec {G},\sigma )=\vec {G}(T,\sigma )$.

First, assume that *e* is redundant and thus, $\vec {G}(T_e,\sigma )=\vec {G}(T,\sigma )$. Then *e* must be an inner edge, since otherwise $L(T)\ne L(T_e)$ and, therefore, $(T_e,\sigma )$ does not explain $(\vec {G},\sigma )$. Now assume, for contradiction, that there is an arc $(a,b)\in E(\vec {G})$ such that ${{\,\mathrm{lca}\,}}_T(a,b)=v$ and $\sigma (b)\in \sigma ( L(T(u)){\setminus } L(T(v)))$. Then there is a leaf $b'\in L(T(u)){\setminus } L(T(v))$ with $\sigma (b')=\sigma (b)$ and ${{\,\mathrm{lca}\,}}_T(a,b)=v\prec _{T} u = {{\,\mathrm{lca}\,}}_T(a,b')$. Thus, $(a,b')\notin E(\vec {G})$. After contraction of *e*, we have ${{\,\mathrm{lca}\,}}_T(a,b)={{\,\mathrm{lca}\,}}_T(a,b') = w_e$. Hence, by definition of best matches, (*a*, *b*) is an arc in $\vec {G}(T_e,\sigma )$ if and only if $(a,b')$ is an arc in $\vec {G}(T_e,\sigma )$; a contradiction to the assumption that $(T_e,\sigma )$ explains $(\vec {G},\sigma )$.

Conversely, assume that $e=uv$ with $v\prec _T u$ is an inner edge in *T* and that there is no arc $(a,b)\in E(\vec {G})$ such that ${{\,\mathrm{lca}\,}}_T(a,b)=v$ and $\sigma (b)\in \sigma (L(T(u)){\setminus } L(T(v)))$. In order to show that an edge *e* is redundant, we need to verify that $\vec {G}(T,\sigma ) = \vec {G}(T_e,\sigma )$. To this end, consider an arbitrary leaf $c\in L(T)$. Then we have either Case (1) $c\in L(T){\setminus } L(T(v))$, or Case (2) $c\in L(T(v))$.

In Case (1) it is easy to verify that ${{\,\mathrm{lca}\,}}_{T}(c,d)={{\,\mathrm{lca}\,}}_{T_e}(c,d)$ for every $d\in L(T)$. In particular, therefore, $(c,d)\in E(\vec {G}(T,\sigma ))$ if and only if $(c,d)\in E(\vec {G}(T_e,\sigma ))$.

In Case (2), i.e. $c\in L(T(v))$, consider another, arbitrary, leaf $d\in L(T)$. Note, if $\sigma (c)=\sigma (d)$, then *c* and *d* never form a best match. Thus, we assume $\sigma (c)\ne \sigma (d)$. Now, we consider three mutually exclusive Subcases (a) ${{\,\mathrm{lca}\,}}_T(c,d)\preceq _{T} v$, (b) ${{\,\mathrm{lca}\,}}_T(c,d)=u$ and (c) ${{\,\mathrm{lca}\,}}_T(c,d)\succ _T u$.

*Case (a).* Since no edge below *v* is contracted, we have for every $d'$ with $\sigma (d')=\sigma (d)$, ${{\,\mathrm{lca}\,}}_{T}(c,d')\prec _{T}{{\,\mathrm{lca}\,}}_{T}(c,d)\preceq _{T}v$ if and only if ${{\,\mathrm{lca}\,}}_{T_e}(c,d')\prec _{T_e}{{\,\mathrm{lca}\,}}_{T_e}(c,d)\preceq _{T_e} w_e$. In particular, therefore, $(c,d)\in E(\vec {G}(T,\sigma ))$ if and only if $(c,d)\in E(\vec {G}(T_e,\sigma ))$.

*Case (b).* ${{\,\mathrm{lca}\,}}_T(c,d)=u$ and $c\prec _T v$ implies that $d\in L(T(u){\setminus } L(T(v))$ and thus, $\sigma (d)\in \sigma ( L(T(u)){\setminus } L(T(v)) )$. If $(c,d)\in E(\vec {G}(T,\sigma ))$, then $\sigma (d)\notin \sigma (L(T(v)))$ must hold. Therefore, (*c*, *d*) is still an arc after contraction of *e*. For the case $(c,d)\notin E(\vec {G}(T,\sigma ))$, assume for contradiction $(c,d)\in E(\vec {G}(T_e,\sigma ))$. Then $(c,d)\notin E(\vec {G}(T,\sigma ))$ implies that there must be a vertex $d'$ with $\sigma (d')=\sigma (d)$ and ${{\,\mathrm{lca}\,}}_T(c,d')\preceq _T v\prec _T u={{\,\mathrm{lca}\,}}_T(c,d)$. In particular, $d'\in L(T(v))$ can be chosen such that ${{\,\mathrm{lca}\,}}_T(c,d')$ is farthest away from *v* and thus, $(c,d')\in E(\vec {G}(T,\sigma ))$. Now, ${{\,\mathrm{lca}\,}}_T(c,d')\preceq _T v$ and $(c,d)\in E(\vec {G}(T_e,\sigma ))$ imply that ${{\,\mathrm{lca}\,}}_{T_e}(c,d')= w_e={{\,\mathrm{lca}\,}}_{T_e}(c,d)$, which is only possible if ${{\,\mathrm{lca}\,}}_T(c,d')= v$. In summary, we found an arc $(c,d')\in E(\vec {G}(T,\sigma ))$ with ${{\,\mathrm{lca}\,}}_T(c,d')= v$ and $\sigma (d') \in \sigma ( L(T(u)){\setminus } L(T(v)))$; a contradiction to our assumption. Hence, in Case (b) we have $(c,d)\in E(\vec {G}(T,\sigma ))$ if and only if $(c,d)\in E(\vec {G}(T_e,\sigma ))$.

*Case (c).* Since ${{\,\mathrm{lca}\,}}_T(c,d)\succ _T u$, it is again easy to see that, for every $d'$ with $\sigma (d')=\sigma (d)$, ${{\,\mathrm{lca}\,}}_T(c,d')\prec _T {{\,\mathrm{lca}\,}}_T(c,d)$ if and only if ${{\,\mathrm{lca}\,}}_{T_e}(c,d')\prec _{T_e} {{\,\mathrm{lca}\,}}_{T_e}(c,d)$ and thus, $(c,d)\in E(\vec {G}(T,\sigma ))$ if and only if $(c,d)\in E(\vec {G}(T_e,\sigma ))$.

In summary, we have $(c,d)\in E(\vec {G}(T,\sigma ))$ if and only if $(c,d)\in E(\vec {G}(T_e,\sigma ))$ for all $c,d\in L(T)$. Thus, *e* is redundant. $\square$

As a consequence of Lemma 7, we obtain

### Corollary 1

Let $(T,\sigma )$ be a leaf-colored tree explaining $(G,\sigma )$ and *uv* an inner edge inner of *T* with $v\prec _T u$. If $\sigma (L(T(v)))\cap \sigma (L(T(v')))=\emptyset$ for every $v'\in \mathsf {child}_{T}(u){\setminus }\{v\}$, then *uv* is redundant in *T* (w.r.t. $(G,\sigma )$).

### Proof

If there is an arc $e=(a,b)\in E(\vec {G})$ with ${{\,\mathrm{lca}\,}}_T(a,b)=v$ we have $\sigma (b)\notin L(T(u)){\setminus } L(T(v)) = \cup _{v'\in \mathsf {child}(u){\setminus } \{v\}} L(T(v'))$ because $\sigma (L(T(v)))\cap \sigma (L(T(v')))=\emptyset$ for every $v'\in \mathsf {child}_{T}(u){\setminus }\{v\}$. By Lemma 7, the inner edge *uv* is redundant. $\square$

Both Lemma 7 and Cor. 1 are illustrated in Fig. 12: In (A), *uv* is a non-redundant inner edge since (*a*, *b*) is a best match such that *a* and *b* have *v* as their last common ancestor and the color of *b* is present in another subtree below vertex *u*. Contraction of the edge *uv* would result in a tree $T_{uv}$ in which ${{\,\mathrm{lca}\,}}_{T_{uv}}(a,b)={{\,\mathrm{lca}\,}}_{T_{uv}}(a,b')$, and thus, introduce the additional best match $(a,b')$. Clearly, this cannot occur whenever the other subtrees of *u* do not share any colors with the subtree *T*(*v*), a situation that is shown in (B), i.e., the edge *uv* is redundant w.r.t. the BMG $\vec {G}(T,\sigma )$.

Finally, we show that redundant edges can be contracted in arbitrary order, similar to (Geiß et al. 2019, Lemma 6 & Cor. 2). To this end, we first prove a more general statement.

### Lemma 8

If $T_A$ is obtained from *T* by contracting all edges in a subset *A* of inner edges in *T*, then $\vec {G}(T,\sigma )\subseteq \vec {G}(T_A,\sigma )$.

### Proof

First note that $L(T_A)=L(T)$ since *A* only contains inner edges. Let (*x*, *y*) be an arc in $\vec {G}(T,\sigma )$. This implies that there is no $y'$ with $\sigma (y')=\sigma (y)$ such that ${{\,\mathrm{lca}\,}}_T(x,y')\prec _T{{\,\mathrm{lca}\,}}_T(x,y)$. It is easy to verify that the latter is still true after contraction of an arbitrary edge *e*, i.e. there is no $y'$ with $\sigma (y')=\sigma (y)$ such that ${{\,\mathrm{lca}\,}}_{T_e}(x,y')\prec _{T_e}{{\,\mathrm{lca}\,}}_{T_e}(x,y)$. Hence, (*x*, *y*) is an arc in $\vec {G}(T_e,\sigma )$. Now consider the subsets $A_1\subset A_2\subset \cdots \subset A_{|A|}=A$ where each $|A_i|=i$, $1\le i\le |A|$. The argument above implies $\vec {G}(T,\sigma )\subseteq \vec {G}(T_{A_1},\sigma ) \subseteq \cdots \subseteq \vec {G}(T_{A},\sigma )$, which completes the proof. $\square$

### Lemma 9

Let *A* and *B* be disjoint sets of redundant edges in $(T,\sigma )$ w.r.t. $(\vec {G},\sigma )$ and denote by $T_A$ the tree obtained by contraction of all edges in *A* in arbitrary order. Then *B* is a set of redundant edges in $T_A$ w.r.t. $\vec {G}(T_A,\sigma )=\vec {G}(T,\sigma )$.

### Proof

By Lemma 8, contraction of *any* inner edge $e=uv\in E(T)$ never leads to a loss of arcs in the BMG $(\vec {G},\sigma ) = \vec {G}(T,\sigma )$. Furthermore, the redundant edges in *T* w.r.t. $(G,\sigma )$ are completely characterized by Lemma 7. Thm. 8 in Geiß et al. (2019) states that by contraction of all redundant edges (in an arbitrary order), one obtains the unique least resolved tree $(T^*,\sigma )$ of $(\vec {G},\sigma )$. As argued above, no arc of $\vec {G}(T,\sigma )$ can be lost in the stepwise contraction of redundant edges. Together with $\vec {G}(T,\sigma )=\vec {G}(T^*,\sigma )=(\vec {G},\sigma )$ this implies $\vec {G}(T_A,\sigma )=(\vec {G},\sigma )$. Since by assumption $A\cap B=\emptyset$ and $A\cup B$ is a set of redundant edges w.r.t. $(\vec {G},\sigma )$, we have $(T_A)_B=T_{A\cup B}$ and $\vec {G}(T_A,\sigma )=(\vec {G},\sigma )=\vec {G}(T_{A\cup B},\sigma )=\vec {G}((T_A)_B,\sigma )$. Hence, *B* is a set of redundant edges in $T_A$ w.r.t. $\vec {G}(T_A,\sigma )$. $\square$

## False-positive orthology assignments

### $(T,\sigma )$-fp and *u-fp* edges

The aim of this contribution is to characterize all those false-positive edges in a given BMG $(\vec {G},\sigma )$ that can be identified from the structure of the BMG alone, i.e., without any *a priori* knowledge about the gene tree, the species tree, or the reconciliation map. In this section, we start by considering false-positive edges identifiable with respect to a given $(T,\sigma )$ that explains $(\vec {G},\sigma )$ and then proceed by considering those edges that are identified by *all* trees explaining $(\vec {G},\sigma )$.

#### Definition 10

*(*${(T,\sigma )}$*-false-positive)* Let $(T,\sigma )$ be a tree explaining the BMG $(\vec {G},\sigma )$. An edge *xy* in $\vec {G}$ is called $(T,\sigma )$-*false-positive*, or $(T,\sigma )$-*fp* for short, if for every reconciliation map $\mu$ from $(T,\sigma )$ to any species tree *S* we have $t_\mu ({{\,\mathrm{lca}\,}}_T(x,y))=\square$, i.e., $\mu ({{\,\mathrm{lca}\,}}_T(x,y))\in E(S)$.

In other words, *xy* is called $(T,\sigma )$-*fp* whenever *x* and *y* cannot be orthologous w.r.t. every possible reconciliation $\mu$ from $(T,\sigma )$ to any species tree. Interestingly, $(T,\sigma )$-*fp* s can be identified without considering reconciliation maps explicitly.

#### Lemma 10

Let $(\vec {G},\sigma )$ be a BMG, *xy* be an edge in $\vec {G}$ and $(T,\sigma )$ be a tree that explains $(\vec {G},\sigma )$. Then, the following statements are equivalent:

1. The edge *xy* is $(T,\sigma )$-*fp*.
2. There are two children $v_1$ and $v_2$ of ${{\,\mathrm{lca}\,}}_T(x,y)$ such that $\sigma (L(T(v_1)))\cap \sigma (L(T(v_2)))\ne \emptyset$.
3. For the extremal labeling ${{\widehat{t}}_T}$ of $(T,\sigma )$ it holds that ${{\widehat{t}}_T}({{\,\mathrm{lca}\,}}_T(x,y)) = \square$.

#### Proof

*(2) implies (1).* Suppose that there are two children $v_1$ and $v_2$ of ${{\,\mathrm{lca}\,}}_T(x,y)$ such that $\sigma (L(T(v_1)))\cap \sigma (L(T(v_2)))\ne \emptyset$. By Lemma 2, $\mu ({{\,\mathrm{lca}\,}}_T(x,y))\in E(S)$ and thus, $t_{\mu }({{\,\mathrm{lca}\,}}_T(x,y))=\square$ for all possible reconciliation maps $\mu$ from $(T,\sigma )$ to any species tree *S*. Hence, *xy* is $(T,\sigma )$-*fp*.

*(1) implies (2).* By contraposition, let $v = {{\,\mathrm{lca}\,}}_T(x,y)$ and suppose that for all distinct children $v_i,v_j\in \mathsf {child}(v)=\{v_1,\dots ,v_k\}$, $k\ge 2$ we have $\sigma (L(T(v_i)))\cap \sigma (L(T(v_j)))=\emptyset$. In the following, we show that there is a species tree *S* and a reconciliation map $\mu$ from $(T,\sigma )$ to *S* such that $t_{\mu }({{\,\mathrm{lca}\,}}(x,y))=\newmoon$, which implies that *xy* is not $(T,\sigma )$-*fp*.

We construct the species tree *S* as follows: *S* has root edge $0_S\rho _S$. Now add *k* children $u_1,\dots ,u_k$ to $\rho _S$. For each of these children $u_i$ with $|\sigma (L(T(v_i)))|>1$, we add a leaf *t* for every color $t\in \sigma (L(T(v_i)))$ and the edge $u_it$. Any other $u_i$ is considered to be a leaf in *S*, and we identify $u_i$ with the single element in $\sigma (L(T(v_i)))$. Furthermore, add for all $t\in \sigma (L(T)){\setminus } \sigma (L(T(v)))$ a leaf *t* that is adjacent to $\rho _S$. Since the color sets $\sigma (L(T)){\setminus } \sigma (L(T(v))), \sigma (L(T(v_1))), \dots , \sigma (L(T(v_k))$ are pairwise distinct, *S* is well-defined, and, by construction, a planted phylogenetic tree. To construct a reconciliation map we put (i) $\mu (0_T)= 0_S$; (ii) $\mu (x)=\sigma (x)$ for all $x\in L(T)$; (iii) $\mu (v)=\rho _S$; (iv) $\mu (w)= 0_S\rho _S$ for all $w\in V^0(T {\setminus } T(v))$; and (v) $\mu (w)=\rho _Su_i$ for all $w\in V^0(T(v_i))$. By Condition (i) and (ii), the Axioms (R0) and (R1) are satisfied, respectively. By Condition (v), we have $\mu (v_i)=\rho _Su_i$ if $v_i$ is an inner vertex. Otherwise, $v_i$ is a leaf and $|\sigma (L(T(v_i)))|=1$. Therefore, $\mu (v_i)=\sigma (v_i)=u_i$ by (ii) and by construction. It is easy to verify that $\mu$ satisfies (R2). A sketch of construction of the species tree *S* and the reconciliation map $\mu$ is provided in Fig. 13.

The only vertex of *T* that is mapped to a vertex in *S* is *v*. Hence, it remains to show that $\mu (v)=\rho _S\in V^0(S)$ satisfies (R3). Note that for every two distinct children $v_i, v_j$ of *v* we have $\mu (v_i)\in \{\rho _Su_i, u_i\}$ and $\mu (v_j)\in \{\rho _Su_j, u_j\}$. In any case, $\mu (v_i)$ and $\mu (v_j)$ are incomparable in *S*. Hence, (R3.ii) is satisfied. In particular, $\mu (v) = \rho _S = {{\,\mathrm{lca}\,}}_S(\mu (v_i),\mu (v_j))$ for all distinct $v_i,v_j\in \mathsf {child}(v)$. Hence, (R3.i) is satisfied. In summary, $\mu$ is a reconciliation map from $(T,\sigma )$ to *S*. Since $\mu (v)=\rho _S\in V^0(S)$, we have $t_{\mu }(v)=\newmoon$.

Statements (2) and (3) are equivalent by definition of the extremal event labeling. $\square$

Lemma 10 implies that $(T,\sigma )$-*fp* can be verified in polynomial time for any given gene tree $(T,\sigma )$.

#### Definition 11

*(Unambiguous false-positive)* Let $(\vec {G},\sigma )$ be a BMG. An edge *xy* in $\vec {G}$ is called *unambiguous false-positive (**u-fp**)* if for all trees $(T,\sigma )$ that explain $(\vec {G},\sigma )$ the edge *xy* is $(T,\sigma )$-*fp*.

Hence, if an edge *xy* in $\vec {G}$ is *u-fp*, then it is in particular $(T,\sigma )$-*fp* in the true history that explains $(\vec {G},\sigma )$. Thus, *u-fp* edges are always “correct” false-positives.

### The color-intersection ${\mathcal {S}}^{\cap }$

Given a gene tree $(T,\sigma )$ and a pair of distinct leaves $x,y\in L(T)$, we denote by $v_x, v_y \in \mathsf {child}_T({{\,\mathrm{lca}\,}}_T(x,y))$ the unique children of the last common ancestor of *x* and *y* for which $x\preceq _T v_x$ and $y\preceq _T v_y$. That is, $T(v_x)$ and $T(v_y)$ are the subtrees of *T* rooted in the children of ${{\,\mathrm{lca}\,}}_T(x,y)$ with $x\in L(T(v_x))$ and $y\in L(T(v_y))$. The set

2 $$\begin{aligned} {\mathcal {S}}_T^{\cap }(x,y):=\sigma (L(T(v_x)))\cap \sigma (L(T(v_y))) \end{aligned}$$

contains the colors, i.e. species, that are common to both subtrees. Lemma 4 immediately implies

#### Corollary 2

Let *xy* be an edge in a BMG $(\vec {G},\sigma )$. Then $\sigma (\{x,y\})\cap {\mathcal {S}}_T^{\cap }(x,y)=\emptyset$ for all trees $(T,\sigma )$ that explain $(\vec {G},\sigma )$.

The following result shows that the color-intersection of a given edge in a BMG $(\vec {G},\sigma )$ in fact does not depend on the tree representation of $(\vec {G},\sigma )$.

#### Lemma 11

Let $(\vec {G},\sigma )$ be a BMG and $(T^*,\sigma )$ the corresponding unique least resolved tree explaining $(\vec {G},\sigma )$. Then, for each tree $(T,\sigma )$ that explains $(\vec {G},\sigma )$, every edge *xy* in $(\vec {G},\sigma )$ satisfies ${\mathcal {S}}^{\cap }_{T^*}(x,y)={\mathcal {S}}^{\cap }_T(x,y)$. Thus, in particular, ${\mathcal {S}}_{T^*}^{\cap }(x,y)\ne \emptyset$ if and only if ${\mathcal {S}}_T^{\cap }(x,y) \ne \emptyset$.

#### Proof

Let $(T,\sigma )$ be an arbitrary tree that explains $(\vec {G},\sigma )$. Moreover, let *xy* be an edge in $\vec {G}$ and denote by $v_x$ and $v_y$ be the unique children $v_x, v_y \in \mathsf {child}_T({{\,\mathrm{lca}\,}}_T(x,y))$ with $x\preceq _T v_x$ and $y\preceq _T v_y$. Analogously, $v^*_x$ and $v^*_y$ are the unique children $v^*_x, v^*_y \in \mathsf {child}_{T^*}({{\,\mathrm{lca}\,}}_{T^*}(x,y))$ with $x\preceq _{T^*} v^*_x$ and $y\preceq _{T^*} v^*_y$.

First, we show that $t\in {\mathcal {S}}^{\cap }_{T^*}(x,y)$ implies $t\in {\mathcal {S}}_T^{\cap }(x,y)$. Since $(T,\sigma )$ explains $(\vec {G}, \sigma )$, we apply Thm. 3 to conclude that *T* is a refinement of $T^*$ and thus, ${\mathscr {C}}(T^*)\subseteq {\mathscr {C}}(T)$. Therefore, $L(T^*({{\,\mathrm{lca}\,}}_{T^*}(x,y))$, $L(T^*(v^*_x))$ and $L(T^*(v^*_y))$ are contained in ${\mathscr {C}}(T)$. This implies that there must be vertices *u*, $w_x$, and $w_y$ in *T* with $L(T(u))= L(T^*({{\,\mathrm{lca}\,}}_{T^*}(x,y))$, $L(T(w_x))=L(T^*(v^*_x))$ and $L(T(w_y))=L(T^*(v^*_y))$. Note that $L(T^*(v^*_x))\cap L(T^*(v^*_y))=\emptyset$, and thus $L(T(w_x))\cap L(T(w_y))=\emptyset$. In particular, $w_x$ and $w_y$ are incomparable in *T*. Moreover, $u = {{\,\mathrm{lca}\,}}_T(x,y)={{\,\mathrm{lca}\,}}_T(w_x,w_y)$, thus we have $w_x\preceq _T v_x$ and $w_y\preceq _T v_y$. Therefore, $L(T^*(v^*_x))\subseteq L(T(v_x))$ and $L(T^*(v^*_y))\subseteq L(T(v_y))$. Therefore, $t\in {\mathcal {S}}_{T^*}^{\cap }(x,y)$ implies $t\in {\mathcal {S}}_T^{\cap }(x,y)$.

Now, we show that $t\in {\mathcal {S}}_T^{\cap }(x,y)$ implies $t\in {\mathcal {S}}^{\cap }_{T^*}(x,y)$. Let $t\in {\mathcal {S}}^{\cap }_T(x,y)\ne \emptyset$. In this case, $t\in \sigma (L(T(v_x)))$ and we can choose a vertex $z_1\in L(T(v_x))$ such that $\sigma (z_1)=t$ and ${{\,\mathrm{lca}\,}}_T(x,z_1)$ is as far away as possible from $v_x$ compared to all ${{\,\mathrm{lca}\,}}_T(x,z)$ with $z\in L[t]$, i.e., ${{\,\mathrm{lca}\,}}_T(x,z_1) \preceq _T {{\,\mathrm{lca}\,}}_T(x,z)$ for all $z\in L[t]$. Thus, $(x,z_1)\in E(\vec {G})$. An analogous argument ensures that there is a vertex $z_2\in L(T(v_y))$ such that $\sigma (z_2)=t$ and $(y,z_2)\in E(\vec {G})$. Clearly, ${{\,\mathrm{lca}\,}}_{T}(x,z_2)={{\,\mathrm{lca}\,}}_{T}(x,y)={{\,\mathrm{lca}\,}}_{T}(y,z_1)$ and thus ${{\,\mathrm{lca}\,}}_{T}(x,z_1)\preceq _{T}v_x\prec _T{{\,\mathrm{lca}\,}}_{T}(x,z_2)$, which in turn implies that $(x,z_2)\notin E(\vec {G})$. Since $(x,z_1)\in E(\vec {G})$ and $(x,z_2)\notin E(\vec {G})$, we obtain the informative triple $xz_1|z_2$ for $(\vec {G},\sigma )$. Analogously, $yz_2|z_1$ is an informative triple for $(\vec {G},\sigma )$. Lemma 6 and the fact that $T^*$ explains $(\vec {G},\sigma )$ implies that there are distinct vertices $v_1,v_2\in \mathsf {child}_{T^*}({{\,\mathrm{lca}\,}}_{T^*}(x,y))$ such that $x,z_1\preceq _{T^*}v_1$ and $y,z_2\preceq _{T^*}v_2$. Since $t=\sigma (z_1)=\sigma (z_2)$, we have $t\in {\mathcal {S}}^{\cap }_{T^*}(x,y)$.

Finally, $t\in {\mathcal {S}}^{\cap }_{T^*}(x,y)$ if and only if $t\in {\mathcal {S}}^{\cap }_{T}(x,y)$ implies both ${\mathcal {S}}^{\cap }_{T^*}(x,y)={\mathcal {S}}^{\cap }_{T}(x,y)$ and ${\mathcal {S}}^{\cap }_{T^*}(x,y)\ne \emptyset$ *if and only if* ${\mathcal {S}}^{\cap }_{T}(x,y)\ne \emptyset$. $\square$

#### Remark 1

By Lemma 11, we have ${\mathcal {S}}^{\cap }_{T}(x,y)={\mathcal {S}}^{\cap }_{T^*}(x,y)$ for every tree $(T,\sigma )$ explaining a BMG $(\vec {G},\sigma )$ with corresponding least resolved tree $(T^*,\sigma )$. Therefore, it is sufficient to consider ${\mathcal {S}}^{\cap }_{T^*}(x,y)$. We will therefore drop the explicit reference to the tree and simply write ${\mathcal {S}}^{\cap }(x,y)$. We can verify in polynomial time whether or not ${\mathcal {S}}^{\cap }(x,y)=\emptyset$ because the least resolved tree $(T^*,\sigma )$ explaining $(\vec {G},\sigma )$ can be computed in polynomial time.

#### Proposition 1

Every edge *xy* in a BMG $(\vec {G},\sigma )$ with ${\mathcal {S}}^{\cap }(x,y)\ne \emptyset$ is *u-fp*.

#### Proof

By Lemma 11 and Remark 1, ${\mathcal {S}}^{\cap }(x,y)\ne \emptyset$ if and only if ${\mathcal {S}}^{\cap }_T(x,y)\ne \emptyset$ for all trees $(T,\sigma )$ that explain $(\vec {G},\sigma )$. By Lemma 2, $\mu ({{\,\mathrm{lca}\,}}_T(x,y))\in E(S)$ and thus, $t_{\mu }({{\,\mathrm{lca}\,}}_T(x,y))=\square$ for all trees $(T,\sigma )$ that explain $(\vec {G},\sigma )$. Hence, *xy* is *u-fp*. $\square$

As we shall see later, the converse of Prop. 1 is not always satisfied (cf. also Fig. 14). An immediate consequence of Prop. 1 is:

#### Corollary 3

An edge *xy* in a BMG $\vec {G}(T,\sigma )$ with ${\mathcal {S}}^{\cap }(x,y)\ne \emptyset$ is $(T,\sigma )$-*fp*.

Although not necessarily true in general, we show next that the converse of Prop. 1 and Cor. 3 does hold for the special case of binary trees.

#### Lemma 12

Let *xy* be an edge in $\vec {G}(T,\sigma )$ and suppose ${{\,\mathrm{lca}\,}}_T(x,y)$ is a binary vertex. Then, the following three statements are equivalent:

1. The edge *xy* is $(T,\sigma )$-*fp*.
2. $${\mathcal {S}}^{\cap }(x,y)\ne \emptyset $$.
3. The edge *xy* is *u-fp*.

#### Proof

*(1) implies (2).* Suppose *xy* is $(T,\sigma )$-*fp*. Since *v* is binary, it has precisely two children $v_1$ and $v_2$. In particular, $v={{\,\mathrm{lca}\,}}_T(x,y)$ implies that that $x\preceq _T v_i$ and $x\preceq _T v_j$ for $i,j\in \{1,2\}$ being distinct. By Lemma 10, the two children $v_1$ and $v_2$ of *v* satisfy $\sigma (L(T(v_1)))\cap \sigma (L(T(v_2)))\ne \emptyset$. By Lemma 11 and Remark 11, we have ${\mathcal {S}}^{\cap }(x,y)\ne \emptyset$.

*(2) implies (3).* If ${\mathcal {S}}^{\cap }(x,y)\ne \emptyset$, we can apply Prop. 1 to conclude that *xy* is *u-fp*.

*(3) implies (1).* By definition, if *xy* is *u-fp*, then it is in particular also $(T,\sigma )$-*fp*. $\square$

#### Theorem 4

Let $(\vec {G},\sigma )$ be a BMG that is explained by a binary tree $(T,\sigma )$. Then, for every edge *xy* in $(\vec {G},\sigma )$, the following three statements are equivalent:

1. The edge *xy* is $(T,\sigma )$-*fp*.
2. $${\mathcal {S}}^{\cap }(x,y)\ne \emptyset $$.
3. The edge *xy* is *u-fp*.

#### Proof

For every edge *xy* in $\vec {G}$ the last common ancestor ${{\,\mathrm{lca}\,}}_T(x,y)$ is binary. Now apply Lemma 12. $\square$

Thm. 4 implies that all *u-fp* edges can be detected in a BMG that is explained by a known binary gene tree. However, not all BMGs $(\vec {G},\sigma )$ can be explained by a binary tree, as e.g. the BMG in Fig. 6(A). Thm. 4 does not generalize to the non-binary case, and ${\mathcal {S}}^{\cap }(x,y)$ is not sufficient to identify all *u-fp* edges. Furthermore, it is not difficult to find non-binary trees in which $(T,\sigma )$-*fp* and *u-fp* edges are not the same: As show in Fig. 3, the edge *xz* in is $(T_1,\sigma )$-*fp* but not $(T_2,\sigma )$-*fp* according to Lemma 10. Since both trees explain the same BMG, the edge *xy* is not *u-fp*.

### ${\mathcal {S}}^{\cap }(x,y)\ne \emptyset$: quartets

Since every orthology graph is a cograph (cf. Thm. 1), we know that every induced $P_4$ in the RBMG is associated with false-positive edges. The induced subgraphs of the BMG spanned by a $P_4$ in its symmetric part (i.e., the RBMG) are called quartets. We write $\langle abcd \rangle$ or, equivalently, $\langle dcba \rangle$ for an induced $P_4$ with edges *ab*, *bc*, and *cd*. The quartets on three colors fall into three classes:

#### Definition 12

*(Good, bad, and ugly quartets)* Let $(\vec {G},\sigma )$ be a BMG with symmetric part $(G,\sigma )$ and vertex set *L*, and let $Q:=\{x,y,z,z'\} \subseteq L$ with $x\in L[r]$, $y\in L[s]$, and $z,z'\in L[t]$. The set *Q*, resp., the induced subgraph $(\vec {G}[Q],\sigma _{|Q})$ is

- a *good quartet* if (i) $\langle zxyz'\rangle$ is an induced $P_4$ in $(G,\sigma )$ and (ii) $(z,y),(z',x)\in E(\vec {G})$ and $(y,z),(x,z')\notin E(\vec {G})$,
- a *bad quartet* if (i) $\langle zxyz'\rangle$ is an induced $P_4$ in $(G,\sigma )$ and (ii) $(y,z),(x,z')\in E(\vec {G})$ and $(z,y),(z',x)\notin E(\vec {G})$,
- an *ugly quartet* if $\langle zxz'y\rangle$ is an induced $P_4$ in $(G,\sigma )$.

The edge *xy* in a good quartet $\langle zxyz'\rangle$ is its *middle* edge. The edge *zx* of an ugly quartet $\langle zxz'y\rangle$ or a bad quartet $\langle zxyz'\rangle$ is called its *first* edge. First edges in ugly quartets are uniquely determined due to the colors. In bad quartets, this is not the case and therefore, the edge $yz'$ in $\langle zxyz'\rangle$ is a first edge as well.

An RBMG never contains induced $P_4$s on two colors (Geiß et al. 2020c, Obs. 5). This, in particular, implies that for the induced $P_4$s in Def. 12 the colors *r*, *s*, and *t* must be pairwise distinct. Induced $P_4$s on four colors are investigated in some more detail in Sec. D.3 below.

The key property of good quartets is a consequence of (Geiß et al. 2020b, Cor. 5):

#### Proposition 2

If $\langle zxyz'\rangle$ is a good quartet in the BMG $(\vec {G},\sigma )$, then ${\mathcal {S}}^{\cap }(x,y)\ne \emptyset$ and thus, *xy* is *u-fp*.

#### Proof

Let $\langle zxyz' \rangle$ in $(\vec {G},\sigma )$ be a good quartet in $(\vec {G},\sigma )$ and let $(T,\sigma )$ be an arbitrary tree explaining $(\vec {G},\sigma )$. Then (Geiß et al. 2020c, Lemma 36) implies that $v:={{\,\mathrm{lca}\,}}_T(x,y,z,z')$ has two distinct children $v_1, v_2\in \mathsf {child}(v)$ such that $x,z \preceq _T v_1$ and $y,z'\preceq _T v_2$. Hence, $v={{\,\mathrm{lca}\,}}_T(x,y)$. Since $\sigma (z)\in \sigma (L(T(v_1)))\cap \sigma (L(T(v_2)))$, we have ${\mathcal {S}}^{\cap }(x,y)\ne \emptyset$ and, by Prop. 1, the edge *xy* is *u-fp*. $\square$

Prop. 2 provides a convenient way to identify unambiguous false-positive edges in a BMG.

#### Lemma 13

If *xy* is an edge in a BMG $\vec {G}(T,\sigma )$ and $t\in {\mathcal {S}}^{\cap }(x,y)$, then there is a good quartet $\langle z_1x^*y^*z_2\rangle$ such that

- $$\sigma (x^*)=\sigma (x)$$, $$\sigma (y^*)=\sigma (y)$$, and $$\sigma (z_1)=\sigma (z_2)=t$$;
- $x^*,z_1\in L(T(v_x))$ and $y^*,z_2\in L(T(v_y))$ with $v_x$ and $v_y$ being the unique children in $\mathsf {child}_T ({{\,\mathrm{lca}\,}}_T (x, y))$ such that with $x \preceq _T v_x$ and $y \preceq _T v_y$.

#### Proof

Consider an edge *xy* of $\vec {G}(T,\sigma )$ and a color $t\in {\mathcal {S}}^{\cap }(x,y)$. By Cor. 2, $t\ne \sigma (x),\sigma (y)$. Lemma 3 ensures the existence of an edge $x^*z_1$ in $\vec {G}$ for some leaves $x^*\in L(T(v_x))\cap L[\sigma (x)]$ and $z_1\in L(T(v_x))\cap L[t]$. By the same arguments as in the proof of Cor. 2, we can conclude that $z_1y'$ is not an edge in $\vec {G}$ for all $y'\in L(T(v_y))\cap L[\sigma (y)]$. However, $(z_1,y')\in E(\vec {G})$ since the color of $y'$ is not present in $T(v_x)$. Likewise, there are leaves $y^*\in L(T(v_y))\cap L[\sigma (y)]$ and $z_2\in L(T(v_y))\cap L[t]$ such that $y^*z_2$ forms an edge in $\vec {G}$. Reusing the arguments from $L(T(v_x))$, we find that $x'z_2$ is not an edge in $\vec {G}$ and $(z_2,x')\in E(\vec {G})$ for any $x'\in L(T(v_x))\cap L[\sigma (x)]$. Finally, $\sigma (x)\notin \sigma (L(T(v_y)))$ and $\sigma (y)\notin \sigma (L(T(v_x)))$ implies that $x^*y^*$ forms an edge in $\vec {G}$. Hence, $\langle z_1x^*y^*z_2\rangle$ is a good quartet. $\square$

The edge $x^*y^*$ in Lemma 13 is the middle edge of a good quartet. For completeness, we also provide a result for the identification of *u-fp* edges using bad quartets:

#### Proposition 3

Let $\langle zxyz'\rangle$ be a bad quartet in a BMG $(\vec {G},\sigma )$. Then, the edges *xz* and $yz'$ are *u-fp* and every tree that explains $(\vec {G},\sigma )$ is non-binary.

#### Proof

Let $(T,\sigma )$ be an arbitrary tree that explains $(\vec {G},\sigma )$, set $u:={{\,\mathrm{lca}\,}}_T(x,z)$ and let $v_x,v_z\in \mathsf {child}_T(u)$ be the two distinct children of *u* such that $x\preceq _T v_x$ and $z\preceq _T v_z$. By symmetry, it suffices to show that *xz* is *u-fp*. Since $\langle zxyz'\rangle$ is a bad quartet, we have $(x,z),(x,z')\in E(\vec {G})$ and thus ${{\,\mathrm{lca}\,}}_T(x,z')={{\,\mathrm{lca}\,}}_T(x,z)=u$. Let $v_{z'}\in \mathsf {child}_T(u)$ be the child of *u* such that $z'\preceq _T v_{z'}$. Since ${{\,\mathrm{lca}\,}}_T(x,z')=u$ we have $v_x\ne v_{z'}$. Now, assume for contradiction that $v_z=v_{z'}$, and thus $z'\in L(T(v_z))$. Since $\langle zxyz'\rangle$ is a bad quartet, we have $(z',x)\notin E(\vec {G})$, which implies the existence of a vertex $x'$ with $\sigma (x)=\sigma (x')$ and ${{\,\mathrm{lca}\,}}_T(x', z')\prec _T{{\,\mathrm{lca}\,}}_T(x,z')=u$ and therefore, $x'\in L(T(v_z))$. However, this implies that ${{\,\mathrm{lca}\,}}_T(x',z)\preceq _{T}v_z\prec _T u={{\,\mathrm{lca}\,}}_T(x,z)$, which together with $\sigma (x)=\sigma (x')$ contradicts the fact that *xz* is an edge in $\vec {G}$. Hence, $v_z\ne v_{z'}$. Therefore, $\sigma (z)= \sigma (z')\in \sigma (L(T(v_z)))\cap \sigma (L(T(v_{z'})))\ne \emptyset$ for distinct children $v_z,v_{z'}\in \mathsf {child}_T(u)$. By Lemma 10, the edge *xz* is $(T,\sigma )$-*fp* and since $(T,\sigma )$ was chosen arbitrarily, the edge *xz* is *u-fp*. Moreover, we have shown that $v_x$, $v_z$ and $v_{z'}$ must be pairwise distinct and thus, $(T,\sigma )$ is non-binary. $\square$

Fig. 5 shows that *u-fp* edges *xy* with ${\mathcal {S}}^{\cap }(x,y)\ne \emptyset$ exist that are neither middle edges of good quartets or first edges of bad quartets. Thus we next consider ugly quartets.

#### Proposition 4

If $\langle xyx'z\rangle$ is an ugly quartet in a BMG $(\vec {G},\sigma )$, then the edges *xy* and $yx'$ are *u-fp*.

#### Proof

Consider an ugly quartet $\langle xyx'z\rangle$. Let $(T,\sigma )$ be an arbitrary tree explaining $(\vec {G},\sigma )$, put $u:={{\,\mathrm{lca}\,}}_T(x,y)$ and let $v_x,v_y\in \mathsf {child}_T(u)$ be the two distinct children of *u* such that $x\preceq _T v_x$ and $y\preceq _T v_y$.

Since $x'y$ and *xy* are edges in $\vec {G}$ we have ${{\,\mathrm{lca}\,}}_T(x',y)\preceq _T u$. Moreover, Cor. 2 implies $\sigma (x')=\sigma (x)\notin \sigma (L(T(v_y)))$ and thus $x'\notin L(T(v_y))$. Therefore, ${{\,\mathrm{lca}\,}}_T(x',y)={{\,\mathrm{lca}\,}}_T(x,y)=u$.

Now consider an arbitrary reconciliation map $\mu$ from $(T,\sigma )$ to some species tree *S*. The existence of $\mu$ is guaranteed by Lemma 1. If $x'\notin L(T(v_x))$, then there is a vertex $v_3\in \mathsf {child}_T(u)$, $v_3\ne v_x,v_y$ such that $x'\preceq _T v_3$ and $\sigma (x)=\sigma (x')\in \sigma (L(T(v_x)))\cap \sigma (L(T(v_3))) \ne \emptyset$, which by Lemma 2 implies $t_\mu (u)=\square$.

Now suppose $x'\in L(T(v_x))$ and recall that $x'z$ is an edge in $\vec {G}$ by assumption. Since ${{\,\mathrm{lca}\,}}_T(x',z)$ and ${{\,\mathrm{lca}\,}}_T(x,x')$ are both ancestors of $x'$ they are comparable. If ${{\,\mathrm{lca}\,}}_T(x',z)\succ _T {{\,\mathrm{lca}\,}}_T(x,x')$, then ${{\,\mathrm{lca}\,}}_T(x,z)={{\,\mathrm{lca}\,}}_T(x',z)$. Together with the fact that $x'z$ is an edge in $\vec {G}$ but not *xz*, this implies that there is a $z'\in L[\sigma (z)]$ such that ${{\,\mathrm{lca}\,}}_T(x,z')\prec _T{{\,\mathrm{lca}\,}}_T(x,z)$. This in turn implies ${{\,\mathrm{lca}\,}}_T(x',z')\prec _T{{\,\mathrm{lca}\,}}_T(x',z)$, which contradicts that $x'z$ is an edge in $\vec {G}$. Therefore, $x'\in L(T(v_x))$ implies ${{\,\mathrm{lca}\,}}_T(x',z)\preceq _T {{\,\mathrm{lca}\,}}_T(x,x')$ and $x,x',z\in L(T(v_x))$. Since *yz* is not an edge in $\vec {G}$ by assumption and Cor. 2 implies $\sigma (y)\notin \sigma (L(T(v_x))$, there is a leaf $z'$ with color $\sigma (z')=\sigma (z)$ such that ${{\,\mathrm{lca}\,}}_T(y,z')\prec _T {{\,\mathrm{lca}\,}}_T(y,z)$. This is only possible if $z'\in L(T(v_y))\cap L[\sigma (z)]$. Therefore, $\sigma (z)\in \sigma (L(T(v_x)))\cap \sigma (L(T(v_y)))$ and Lemma 2 implies that $t_\mu (u)=\square$.

In summary, ${{\,\mathrm{lca}\,}}_T(x',y)={{\,\mathrm{lca}\,}}_T(x,y)=u$ and $t_\mu (u)=\square$ for every tree explaining $(\vec {G},\sigma )$ and every possible reconciliation map $\mu$ from $(T,\sigma )$ to any species tree. Thus both *xy* and $x'y$ are *u-fp*. $\square$

#### Proposition 5

Let $(\vec {G},\sigma )$ be a BMG and *xy* an edge in $\vec {G}$ with ${\mathcal {S}}^{\cap }(x,y)\ne \emptyset$. Then *xy* is either the middle edge of some good quartet $\langle zxyz'\rangle$ or the first edge in some ugly quartet $\langle xyx'z\rangle$ or $\langle yxy'z\rangle$.

#### Proof

Let $(T,\sigma )$ be a leaf-colored tree explaining the BMG $(\vec {G},\sigma )$ with symmetric part $(G,\sigma )$. Let $v_x, v_y \in \mathsf {child}_T({{\,\mathrm{lca}\,}}_T(x,y))$ such that $x\preceq _T v_x$ and $y\preceq _T v_y$. Since ${\mathcal {S}}^{\cap }(x,y)\ne \emptyset$, Lemma 13 implies that there is a good quartet $\langle z_1x^*y^*z_2\rangle$ with $\sigma (x^*)=\sigma (x)$, $\sigma (y^*)=\sigma (y)$, $\sigma (z_1)=\sigma (z_2)=t\in {\mathcal {S}}^{\cap }(x,y)$, $x^*,z_1\in L(T(v_x))$ and $y^*,z_2\in L(T(v_y))$.

If $x=x^*$ and $y=y^*$ we are done. By symmetry it suffices to consider the case $x\ne x^*$. Before we proceed, we consider the (non-)existence of certain edges in the RBMG $G(T,\sigma )$ and the BMG $\vec {G}(T,\sigma )$. By definition of good quartets, we have $x^*z_1,x^*y^*,y^*z_2\in E(G)$ and Cor. 2 implies $\sigma (x),\sigma (y)\notin {\mathcal {S}}^{\cap }(x,y)$. Hence, $\sigma (x^*)=\sigma (x)\notin \sigma (L(T(v_y)))$ and $\sigma (y^*)=\sigma (y)\notin \sigma (L(T(v_x)))$, and thus $x^*y\in E(G)$ and $xy^*\in E(G)$. Moreover, since ${{\,\mathrm{lca}\,}}_T(y,z_2)\prec _T{{\,\mathrm{lca}\,}}_T(y,z_1)$, we have $yz_1\notin E(G)$. Similarly, $xz_2\notin E(G)$. However, $\sigma (x)\notin \sigma (L(T(v_y)))$ implies that ${{\,\mathrm{lca}\,}}_T(z_2,x) = {{\,\mathrm{lca}\,}}_T(x,y)\preceq {{\,\mathrm{lca}\,}}_T(z_2,x')$ for all $x'\in L[\sigma (x)]$ and thus, $(z_2,x)\in E(\vec {G})$. Similarly, $(z_1,y)\in E(\vec {G})$. Furthermore, we note that neither *x* and $x^*$ nor *y* and $y^*$ can be adjacent in *G* or $\vec {G}$ since $\sigma (x)=\sigma (x^*)$ and $\sigma (y)=\sigma (y^*)$.

If $xz_1 \notin E(G)$, then $\langle xyx^*z_1\rangle$ forms an ugly quartet. Now suppose that $xz_1 \in E(G)$. Assume that there is an edge $yz'\in E(G)$ with $z'\in L(T(v_y))\cap L[t]$. Then, ${{\,\mathrm{lca}\,}}(x,z_1)\prec _T{{\,\mathrm{lca}\,}}(x,z')$ implies $xz'\notin E(G)$. Moreover, since $\sigma (x)\notin \sigma (L(T(v_y)))$ we have, by similar arguments as above, that $(z',x)\in E(\vec {G})$. Thus, $\langle z'yxz_1\rangle$ forms a good quartet. Finally, if there is no such edge $yz'\in E(G)$ then, in particular, $yz_2\notin E(G)$ and $y\ne y^*$. In this case, $\langle yxy^*z_2\rangle$ forms an ugly quartet. $\square$

The example Fig. 14 shows that the converse of Prop. 5 is not true in general.

We summarize the results of Props. 1, 2, 4 and 5 in the following

#### Corollary 4

Let $(\vec {G},\sigma )$ be a BMG that contains the edge *xy*. Then, ${\mathcal {S}}^{\cap }(x,y)\ne \emptyset$ implies that *xy* is either the middle edge of some good quartet or the first edge of some ugly quartet, which in turn implies that *xy* is *u-fp*.

### ${\mathcal {S}}^{\cap }(x,y)=\emptyset$: hourglasses

The case ${\mathcal {S}}^{\cap }(x,y)\ne \emptyset$ is sufficient to detect the edge *xy* as *u-fp*. In this section we turn to the case ${\mathcal {S}}^{\cap }(x,y)=\emptyset$ and show how to identify further *u-fp* edges.

#### Definition 13

*(Hourglass)* An *hourglass* in a proper vertex-colored graph $(\vec {G},\sigma )$, denoted by , is a subgraph $(\vec {G}[Q],\sigma _{|Q})$ induced by a set of four pairwise distinct vertices $Q=\{x, x', y, y'\}\subseteq V(\vec {G})$ such that (i) $\sigma (x)=\sigma (x')\ne \sigma (y)=\sigma (y')$, (ii) *xy* and $x'y'$ are edges in $\vec {G}$, (iii) $(x,y'),(y,x')\in E(\vec {G})$, and (iv) $(y',x),(x',y)\notin E(\vec {G})$.

Note that Condition (i) rules out arcs between $x,x'$ and $y,y'$, respectively, i.e., the only arcs in an hourglass are the ones specified by Conditions (ii) and (iii). An example is shown in Fig. 6(A).

#### Observation 5

Every hourglass is a BMG since it can be explained by a tree as shown in Fig. 6(B).

We first show that hourglasses cannot appear in a BMG that can be explained by a binary tree.

#### Lemma 14

If $(\vec {G},\sigma )$ is a BMG containing the hourglass , then every tree $(T,\sigma )$ that explains $(\vec {G},\sigma )$ contains a vertex $u\in V^0(T)$ with three distinct children $v_1$, $v_2$, and $v_3$ such that $x\preceq _T v_1$, ${{\,\mathrm{lca}\,}}_T(x',y')\preceq _T v_2$ and $y\preceq _T v_3$.

#### Proof

By assumption, *xy* and $x'y'$ are edges in $\vec {G}$, $(x,y'),(y,x')\in E(\vec {G})$, and $(y',x),(x',y)\notin E(\vec {G})$. By Lemma 5, the informative triples $x'y'|x$ and $x'y'|y$ thus must be displayed by every tree $(T,\sigma )$ that explains $(\vec {G},\sigma )$. Thus $u_{x'y'}:={{\,\mathrm{lca}\,}}_T(x',y') \prec _T u_x:={{\,\mathrm{lca}\,}}_T(x,u_{x'y'})$ and $u_{x'y'} \prec _T u_y:={{\,\mathrm{lca}\,}}_T(y,u_{x'y'})$. Furthermore, $u_x$ and $u_y$ are both ancestors of $u_{x'y'}$ and thus comparable w.r.t. $\preceq _T$. If $u_x\prec _T u_y$, then ${{\,\mathrm{lca}\,}}_T(x,y')\prec _T{{\,\mathrm{lca}\,}}_T(x,y)$ which implies that *xy* cannot form an edge in $\vec {G}$; a contradiction. By similar arguments, $u_y\prec _T u_x$ is not possible and therefore, $u_x=u_y=:u$.

Since $u_{x'y'}\prec _T u$, there are two distinct children $v_1,v_2\in \mathsf {child}_T(u)$ of *u* such that $x\preceq _T v_1$ and $u_{x'y'}\preceq _T v_2$. Clearly, $y\notin L(T(v_2))$ since ${{\,\mathrm{lca}\,}}_T(y,u_{x'y'})=u\succ _T v_2$. We also have $y\notin L(T(v_1))$ since $y\in L(T(v_1))$ would imply ${{\,\mathrm{lca}\,}}_T(x,y)\preceq _T v_1\prec _T u={{\,\mathrm{lca}\,}}_T(x,u_{x'y'})={{\,\mathrm{lca}\,}}_T(x,y')$, contradicting $(x,y')\in E(\vec {G})$. Together with $y\in L(T(u))$, this implies the existence of a vertex $v_3\in \mathsf {child}(u)$ such that $v_3\notin \{v_1,v_2\}$ and $y\preceq _T v_3$. $\square$

The result shows that hourglasses  can be used to identify false-positive edges *xy* with ${\mathcal {S}}^{\cap }(x,y)=\emptyset$.

#### Proposition 6

If a BMG $(\vec {G},\sigma )$ contains an hourglass , then the edge *xy* is *u-fp*.

#### Proof

According to Lemma 14, every tree $(T,\sigma )$ that explains $(\vec {G},\sigma )$ contains a vertex $u\in V^0(T)$ with three distinct children $v_1$, $v_2$, and $v_3$ such that $x\preceq _T v_1$, ${{\,\mathrm{lca}\,}}_T(x',y')\preceq _T v_2$ and $y\preceq _T v_3$. Thus, $u={{\,\mathrm{lca}\,}}_T(x,y)$ and $\sigma (x)\in \sigma (L(T(v_1)))\cap \sigma (L(T(v_2)))$. Hence, we can apply Lemma 10 to conclude that *xy* is $(T,\sigma )$-*fp* for every tree that explains $(\vec {G},\sigma )$. Therefore, the edge *xy* is *u-fp*. $\square$

Prop. 6 implies that there are *u-fp* edges that are not contained in a quartet, since an hourglass (see Fig. 6(A)) does not contain a $P_4$. We next generalize the concept of hourglasses.

#### Definition 14

*(Hourglass chain)* An *hourglass chain* ${\mathfrak {H}}$ in a graph $(\vec {G},\sigma )$ is a sequence of $k\ge 1$ hourglasses  such that the following two conditions are satisfied for all $i\in \{1,\dots ,k-1\}$:

*(H1)*: $$y_i=x'_{i+1}$$ and $$y'_i=x_{i+1}$$, and
*(H2)*: $x_i y'_j$ is an edge in $\vec {G}$ for all $j\in \{i+1,\dots ,k\}$

A vertex *z* is called a *left* (resp., *right*) *tail* of the hourglass chain ${\mathfrak {H}}$ if it holds that $(z,x_1)\in E(\vec {G})$ and $(z,x'_1)\notin E(\vec {G})$ (resp., $(z,y_k)\in E(\vec {G})$ and $(z,y'_k)\notin E(\vec {G})$). We call ${\mathfrak {H}}$ *tailed* if it has a left or right tail.

Note that in contrast to good and bad quartets as well as individual hourglasses, an hourglass chain in $(\vec {G},\sigma )$ is not necessarily an induced subgraph.

#### Observation 6

If  be an hourglass chain in $(\vec {G},\sigma )$, then  is an hourglass chain in $(\vec {G},\sigma )$ for every $1\le i < j \le k$.

Hourglass chains are composed of “overlapping” hourglasses. The additional condition that $x_i y'_j\in E(G)$ for all $1\le i<j\le k$ ensures that the two pairs $x'_k,y'_k$ and $x'_l,y'_l$ with $k\ne l$ cannot lie in the same subtree below the last common ancestor *u* which is common to all hourglasses in the chain.

#### Lemma 15

Let  be an hourglass chain in a BMG $(\vec {G},\sigma )$. Then, for every tree $(T,\sigma )$ that explains $(\vec {G},\sigma )$ there is a vertex $u\in V^0(T)$ with pairwise distinct children $v_0,v_1,\dots ,v_k,v_{k+1}$ such that $x_1\in L(T(v_0))$, $y_k\in L(T(v_{k+1}))$, and, for all $1\le i\le k$, we have $x'_i,y'_i\in L(T(v_i))$.

#### Proof

We prove the statement by induction on *k*. For the base case $k=1$, observe that the hourglass  together with Lemma 14 implies that there is a vertex $u\in V^0(T)$ with pairwise distinct children $v_0,v_1$ and $v_2$ such that $x_1\preceq _T v_0$, ${{\,\mathrm{lca}\,}}_T(x'_1,y'_1)\preceq _T v_1$ (thus $x'_1,y'_1\preceq _T v_1$) and $y_1\preceq _T v_2$.

Now let $k>1$ and assume that the statement is true for all hourglass chains containing less than *k* hourglasses. Let  be an hourglass chain. By induction hypothesis, for every subsequence  of ${\mathfrak {H}}$ with $1\le i< k$, which by Obs. 6 is again an hourglass chain, the statement is true.

Consider the subsequence ${\mathfrak {H}}_{i|}$ with $i=k-1$. By assumption, there is a vertex $u\in V^0(T)$ with pairwise distinct children $v_0,v_1,\dots ,v_i,v_{i+1}$ such that it holds $x_1\in L(T(v_0))$, $y_i\in L(T(v_{i+1}))$, and, for all $1\le j\le i$, we have $x'_j,y'_j\in L(T(v_j))$. The hourglass  and Lemma 14 imply the existence of a vertex $u'\in V^0(T)$ with pairwise distinct children $v'_i,v'_{i+1}$ and $v'_{i+2}$ such that $x_{i+1}\preceq _T v'_i$, ${{\,\mathrm{lca}\,}}_T(x'_{i+1},y'_{i+1})\preceq _T v'_{i+1}$ and $y_{i+1}\preceq _T v'_{i+2}$. By the definition of hourglass chains, we have $y_i=x'_{i+1}$ and $y'_i=x_{i+1}$. Therefore, $u'={{\,\mathrm{lca}\,}}_T(x'_{i+1},x_{i+1})={{\,\mathrm{lca}\,}}_T(y_i,y'_i)=u$. Since $v_i$ and $v'_i$ are both children of *u*, $y'_i=x_{i+1}$ and it holds both that $y'_i\preceq _T v_i$ and $x_{i+1}\preceq _T v'_i$, we conclude that $v_i=v'_i$. Similarly, it holds $v_{i+1}=v'_{i+1}$ since $v_{i+1},v'_{i+1}\in \mathsf {child}(u)$ and $y_i=x'_{i+1}$. In particular, we have $v'_{i+2}\ne v'_{i+1}=v_{i+1}$ and $v'_{i+2}\ne v'_{i}=v_{i}$. It remains to show that $v'_{i+2}\ne v_j$ for $0\le j<i$. Assume, for contradiction, that $v'_{i+2}=v_j$ for some fixed *j* with $0\le j<i$. By assumption, $x_1\preceq _T v_j$ if $j=0$, and otherwise, $x_{j+1}=y'_j\preceq _T v_j$. Moreover, since $v'_{i+2}=v_j$, we have $y_{i+1}\preceq _T v_j$. Hence, ${{\,\mathrm{lca}\,}}_T(x_{j+1},y_{i+1})\preceq _T v_j$. Furthermore, since $y'_{i+1}\preceq _T v_{i+1}\ne v_j$, it holds ${{\,\mathrm{lca}\,}}_T(x_{j+1},y'_{i+1})=u\succ _T v_j$. Since $\sigma (y_{i+1})=\sigma (y'_{i+1})$ by the definition of hourglasses, the latter two arguments contradict $x_{j+1}y'_{i+1}\in E(G)$, which must hold by the definition of hourglass chains. Hence, we can conclude that $v'_{i+2}\ne v_j$ for and $0\le j<i$ and we set $v_{i+2}:=v'_{i+2}$. In summary, the statement holds for the hourglass chain ${\mathfrak {H}}_{i+1|} = {\mathfrak {H}}$. $\square$

It is straightforward to generalize the latter statement to tailed hourglass chains.

#### Lemma 16

Let  be an hourglass chain with left (resp. right) tail *z* in a BMG $(\vec {G},\sigma )$. Then, every tree $(T,\sigma )$ that explains $(\vec {G},\sigma )$ contains a vertex $u\in V^0(T)$ with pairwise distinct children $v_0,v_1,\dots ,v_k,v_{k+1}$ such that it holds $x_1\in L(T(v_0))$, $y_k\in L(T(v_{k+1}))$, and, for all $1\le i\le k$, we have $x'_i,y'_i\in L(T(v_i))$. Furthermore, we have $z\preceq _T v_0$ (resp. $z\preceq _T v_{k+1}$).

#### Proof

By Lemma 15, there is a vertex $u\in V^0(T)$ with pairwise distinct children $v_0,v_1,\dots ,v_k,v_{k+1}$ such that it holds $x_1\in L(T(v_0))$, $y_k\in L(T(v_{k+1}))$, and, for all $1\le i\le k$, we have $x'_i,y'_i\in L(T(v_i))$.

Suppose that *z* is a left tail of ${\mathfrak {H}}$. We need to show that $z\preceq _T v_0$. By definition, $(z,x_1)\in E(\vec {G})$, $(z,x'_1)\notin E(\vec {G})$, and $\sigma (x_1)=\sigma (x'_1)$. Therefore, $zx_1|x'_1$ is an informative triple for $(\vec {G},\sigma )$, and hence ${{\,\mathrm{lca}\,}}_{T}(z,x_1) \prec _T{{\,\mathrm{lca}\,}}_{T}(z,x'_1)={{\,\mathrm{lca}\,}}_{T}(x_1,x'_1)=u$. Since $v_0$ is the unique child of *u* with $x_1\prec _T v_0$, we can conclude that ${{\,\mathrm{lca}\,}}_{T}(z,x_1)\preceq _{T} v_0$ and thus, $z\preceq _{T}v_0$.

If *z* is a right tail of ${\mathfrak {H}}$, a similar argument using the informative triple $z'y_k|y'_k$, which must be displayed by *T* because $(z,y_k)\in E(\vec {G})$ and $(z,y'_k)\notin E(\vec {G})$, implies $z\preceq _T v_{k+1}$. $\square$

We are now in the position to show that hourglass chains identify additional *u-fp* edges that are not contained in a single hourglass.

#### Lemma 17

Let  be an hourglass chain in $(\vec {G},\sigma )$, possibly with a left tail *z* or a right tail $z'$. Then every edge $e\in \{x_1y_k, zy_k, x_1z', zz'\}\cap E(G)$ is *u-fp*, where *G* denotes the symmetric part of $\vec {G}$.

#### Proof

Let $(T,\sigma )$ be an arbitrary tree that explains $(\vec {G},\sigma )$. By the definition of hourglass chains, we have $k\ge 1$. Hence, the sequence contains at least the hourglass . Since  in $\vec {G}(T,\sigma )$, Lemma 16 implies the existence of a vertex $u\in V^0(T)$ with pairwise distinct children $v_0,v_1,\dots ,v_k,v_{k+1}$ such that it holds $x_1\in L(T(v_0))$, $y_k\in L(T(v_{k+1}))$, and, for all $1\le i\le k$, we have $x'_i,y'_i\in L(T(v_i))$. Furthermore, this lemma also implies $z\preceq _T v_0$ if *z* is a left tail of ${\mathfrak {H}}$, and $z'\preceq _T v_{k+1}$ if $z'$ is a right tail of ${\mathfrak {H}}$. Note that ${{\,\mathrm{lca}\,}}_T(x_1,x'_1)=u$, and $x_1$ and $x'_1$ lie below distinct children of *u*. More precisely $x_1\preceq _T v_0$ and $x'_1\preceq _T v_1$. Since $\sigma (x_1)=\sigma (x'_1)$, we have $\sigma (L(T(v_0)))\cap \sigma (L(T(v_1)))\ne \emptyset$. Moreover, ${{\,\mathrm{lca}\,}}_{T}(a,b)=u$ for every edge $e=ab$ in $\vec {G}$ that coincides with one of $x_1y_k$, $zy_k$, $x_1z'$, and $zz'$. The latter two arguments together with Lemma 10 imply that every such edge is $(T,\sigma )$-*fp*. Since $(T,\sigma )$ was chosen arbitrarily, every such edge is also *u-fp*. $\square$

It is important to note that the construction of hourglass chains does not imply that an edge $e\in \{x_1y_k, zy_k, x_1z', zz'\}$ must exist in $(\vec {G},\sigma )$. Nevertheless, whenever such an edge occurs, it is *u-fp*. We will take a closer look at the properties of hourglass chains in Sec. D.

## Characterization of unambiguous false-positive edges

### Color-set intersection graphs

In this section, we take a closer look at the trees that explain a given BMG. In particular, we consider the color allocation to the subtrees below each vertex of a tree explaining a given BMG. This leads us to the idea of a color intersection graph.

#### Definition 15

The *color-set intersection graph* ${\mathfrak {C}}_T(u)$ of an inner vertex *u* of a leaf-colored gene tree $(T,\sigma )$ is the undirected graph with vertex set $V:=\mathsf {child}_T(u)$ and edge set

$$\begin{aligned} E:=\{ v_1v_2 \mid v_1,v_2\in V \text {, }v_1\ne v_2 \text { and } \sigma (L(T(v_1)))\cap \sigma (L(T(v_2)))\ne \emptyset \}. \end{aligned}$$

Shortest paths in the color-set intersection graphs will play an important role in identifying many *u-fp* edges.

#### Lemma 18

Let $v_1$ and $v_k$ be two distinct vertices in the same connected component of the color-set intersection graph ${\mathfrak {C}}_T(u)$ of a leaf-colored gene tree $(T,\sigma )$, and let $P(v_1,v_{k}) = (v_1, \dots , v_{k})$ be a shortest path in ${\mathfrak {C}}_T(u)$ connecting $v_1$ and $v_k$. Then $\sigma (L(T(v_i)))\cap \sigma (L(T(v_j)))=\emptyset$ for all *i* and *j* satisfying $1\le i<i+2 \le j\le k$.

#### Proof

Assume, for contradiction, that $\sigma (L(T(v_i)))\cap \sigma (L(T(v_j)))\ne \emptyset$ for some *i*, *j* with $1\le i<i+2 \le j\le k$. Then the edge $v_iv_j$ must be contained in ${\mathfrak {C}}_T(u)$, contradicting the fact that $P(v_1,v_{k})$ is a *shortest* path. $\square$

The following lemma establishes a close connection between color-set intersection graphs and hourglass chains.

#### Lemma 19

Let $(\vec {G},\sigma )$ be a BMG that is explained by $(T,\sigma )$ and suppose that $x,y\in L(T)$ are two distinct leaves with $u:={{\,\mathrm{lca}\,}}_T(x,y)$ and $v_x, v_y\in \mathsf {child}_T(u)$ such that (i) $x\preceq _T v_x$ and $y\preceq _T v_y$, and (ii) there is a shortest path $(v_x=v_0,v_1, \dots , v_{k}, v_{k+1}=v_y)$ of length at least two in ${\mathfrak {C}}_T(u)$. Then there is an hourglass chain  in $(\vec {G},\sigma )$. In particular, precisely one of the following conditions is satisfied:

1. $$x_1=x$$ and $$y_k=y$$;
2. $y_k=y$ and $z:=x$ is a left tail of ${\mathfrak {H}}$;
3. $x_1=x$ and $z':=y$ is a right tail of ${\mathfrak {H}}$; or
4. $z:=x$ is a left tail and $z':=y$ is a right tail of ${\mathfrak {H}}$.

#### Proof

Lemma 18 implies ${\mathcal {S}}^{\cap }(x,y)=\sigma (L(T(v_x)))\cap \sigma (L(T(v_y))) = \sigma (L(T(v_0)))\cap \sigma (L(T(v_{k+1})))=\emptyset$. We proceed by showing that the BMG $\vec {G}(T,\sigma )$ contains an hourglass chain  possibly with left tail *z* and right tail $z'$ such that one of the Conditions 1–4 is satisfied.

We first consider the two cases: either (A) $\sigma (x)\in \sigma (L(T(v_1)))$ or (B) $\sigma (x)\notin \sigma (L(T(v_1)))$. In Case (A), we set $x_1:=x$ and $c_0:=\sigma (x)$. In Case (B), we set $z:=x$, choose $c_0 \in \sigma (L(T(v_0)))\cap \sigma (L(T(v_1)))$ arbitrarily (note $v_0v_1$ forms an edge in ${\mathfrak {C}}_T(u)$ and thus, the latter intersection is non-empty) and we set $x_1 = v$ for some $v\in L(T(v_0))\cap L[c_0]$ such that ${{\,\mathrm{lca}\,}}(v,x)\preceq _T {{\,\mathrm{lca}\,}}_T(v',x)\preceq _T v_0$ for all $v'\in L(T(v_0))\cap L[c_0]$. Clearly, such a vertex *v* exists. Moreover, $c_0\ne \sigma (x)$ and we obtain $(x, v) = (z,x_1)\in E(\vec {G})$ as necessary requirement for left tails. In summary, we have in Case (A) $x_1=x$ and in Case (B) *x* plays the role of the left tail *z* and $x_1$ is some other vertex. Moreover, in both Cases (A) and (B), we have $\sigma (x_1)=c_0\in \sigma (L(T(v_0)))\cap \sigma (L(T(v_1)))$.

We now consider the “other end” of the hourglass chain, that is, vertex $y_k$ and the possible right tail. Again, we have two cases: either (A’) $\sigma (y)\in \sigma (L(T(v_{k+1})))$ or (B’) $\sigma (y)\notin \sigma (L(T(v_{k+1})))$. In Case (A’), we set $y_k:=y$ and $c_k:=\sigma (y)$. In Case (B’), we set $z':=y$, and , by similar arguments as in Case (A) and (B), we can choose $c_k \in \sigma (L(T(v_k)))\cap \sigma (L(T(v_{k+1})))$ arbitrarily and set $y_k = w$ for some vertex $w\in L(T(v_{k+1}))\cap L[c_k]$ such that $(y,w) = (z',y_k)\in E(\vec {G})$ as a necessary requirement for right tails. Again, for both cases (A’) and (B’) we have $\sigma (y_k)=c_k\in \sigma (L(T(v_k)))\cap \sigma (L(T(v_{k+1})))$.

We continue by picking an arbitrary color $c_i$ from $\sigma (L(T(v_i)))\cap \sigma (L(T(v_{i+1})))$ for each $1\le i < k$. This is possible because $v_i v_{i+1}\in E({\mathfrak {C}}_T(u))$, and thus $\sigma (L(T(v_i)))\cap \sigma (L(T(v_{i+1})))\ne \emptyset$. Note that now $c_i\in \sigma (L(T(v_i)))\cap \sigma (L(T(v_{i+1})))$ holds for all $0\le i \le k$. In particular, the colors $c_0,c_1,\dots ,c_k$ are pairwise distinct. To see this, assume, for contradiction, that $c_i=c_j$ for some *i*, *j* with $i<j$. Then $c_i\in \sigma (L(T(v_i)))$ and $c_i=c_j\in \sigma (L(T(v_{j+1})))$ which implies $c_i\in \sigma (L(T(v_i)))\cap \sigma (L(T(v_{j+1})))$. This contradicts Lemma 18 for $j+1\ge i+2$.

For each $1\le i\le k$, we have $c_{i-1}, c_i\in \sigma (L(T(v_i)))$. Thus Lemma 3 ensures the existence of vertices $x'_i\in L(T(v_i))\cap L[c_{i-1}]$ and $y'_i\in L(T(v_i))\cap L[c_{i}]$ that form an edge $x'_i y'_i$ in $\vec {G}$. By assumption we have $x'_i y'_i\in E(G)$ for all $1\le i\le k$ since  is an hourglass. We already set $x_1$ and $y_k$. We furthermore set $x_i:=y'_{i-1}$ for all $1<i\le k$, and $y_i:=x'_{i+1}$ for all $1\le i < k$. Thus ensures that (H1) in Def. 14 is satisfied. Moreover, since $\sigma (x_1)=c_0=\sigma (x'_1)$ and $\sigma (x_i)=\sigma (y'_{i-1})=c_{i-1}$ for all $1<i\le k$, we have $\sigma (x_i)=c_{i-1}=\sigma (x'_i)$ for all $1\le i\le k$. Similar arguments imply $\sigma (y_i)=c_{i}=\sigma (y'_i)$ for all $1\le i\le k$.

We next show that the induced subgraph $\vec {G}[x_i,x'_i,y_i,y'_i]$ is an hourglass for $1\le i \le k$ and thus $x_i y'_j$ is an edge in $\vec {G}$ for all $i<j\le k$. We also know, by construction, that $x'_i y'_i$ is an edge in $\vec {G}$.

Independent of whether $x_1$ was constructed based on the cases (A) or (B), we have $x_i\preceq _T v_0$ if $i=1$ and $x_i=y'_{i-1}\preceq _T v_{i-1}$ otherwise. Thus $x_i\preceq _T v_{i-1}$. Likewise, independent of whether $y_k$ was constructed based on the cases (A’) or (B’), we have $y_i\preceq _T v_{k+1}$ if $i=k$ and $y_i=x'_{i+1}\preceq _T v_{i+1}$ otherwise. Thus $y_i\preceq _T v_{i+1}$. In summary, we have $x_i\preceq _T v_{i-1}$; $x'_i,y'_i\preceq _T v_{i}$; and $y_i\preceq _T v_{i+1}$ for all $i\in \{1,\dots ,k\}$. This implies ${{\,\mathrm{lca}\,}}_T(x_i,y'_i)={{\,\mathrm{lca}\,}}_T(x_i,y_i)={{\,\mathrm{lca}\,}}_T(x'_i,y_i)=u$. Since $i+1 \ge (i-1)+2$ and $P(v_0,v_{k+1})$ is a shortest path, Lemma 18 implies $\sigma (L(T(v_{i-1})))\cap \sigma (L(T(v_{i+1})))=\emptyset$.

From $\sigma (x_i)\in \sigma (L(T(v_{i-1})))$ and $\sigma (y_i)\in \sigma (L(T(v_{i+1})))$ we obtain $\sigma (x_i)\notin \sigma (L(T(v_{i+1})))$ and $\sigma (y_i)\notin \sigma (L(T(v_{i-1})))$. Thus, there is no ${\widetilde{y}}$ such that $\sigma ({\widetilde{y}})=\sigma (y'_i)=\sigma (y_i)$ and ${{\,\mathrm{lca}\,}}_T(x_i,{\widetilde{y}})\prec _T u ={{\,\mathrm{lca}\,}}_T(x_i,y'_i)={{\,\mathrm{lca}\,}}_T(x_i,y_i)$, and no ${\widetilde{x}}$ such that $\sigma ({\widetilde{x}})=\sigma (x'_i)=\sigma (x_i)$ and ${{\,\mathrm{lca}\,}}_T(y_i,{\widetilde{x}})\prec _T u ={{\,\mathrm{lca}\,}}_T(y_i,x'_i)={{\,\mathrm{lca}\,}}_T(y_i,x_i)$. Hence, $\vec {G}$ contains the arcs $(x_i,y'_i)$, $(x_i,y_i)$, $(y_i,x_i)$ and $(y_i,x'_i)$. Moreover, $x_i y_i$ is an edge in $\vec {G}$. However, since $\sigma (x'_i)=\sigma (x_i)$ and ${{\,\mathrm{lca}\,}}_T(x'_i,y'_i)\preceq _T v_i\prec _T u = {{\,\mathrm{lca}\,}}_T(x_i,y'_i)$ we conclude $(y'_i,x_i)\notin E(\vec {G})$. Likewise, $\sigma (y'_i)=\sigma (y_i)$ and ${{\,\mathrm{lca}\,}}_T(x'_i,y'_i)\preceq _T v_i\prec _T u = {{\,\mathrm{lca}\,}}_T(x'_i,y_i)$ imply that $(x'_i,y_i)\notin E(\vec {G})$. In summary,  is an hourglass, for all $i\in \{1,\dots ,k\}$, and $x_i\preceq _T v_{i-1}$ and $y'_j\preceq _T v_{j}$ for all $1\le i<j\le k$.

Since $j\ge (i-1)+2$ and $P(v_0,v_{k+1})$ is a shortest path, Lemma 18 implies that $\sigma (L(T(v_{i-1})))\cap \sigma (L(T(v_{j})))=\emptyset$. Thus, there is no ${\widetilde{y}}$ such that $\sigma ({\widetilde{y}})=\sigma (y'_j)$ and ${{\,\mathrm{lca}\,}}_T(x_i,{\widetilde{y}})\prec _T u ={{\,\mathrm{lca}\,}}_T(x_i,y'_j)$, and no ${\widetilde{x}}$ such that $\sigma ({\widetilde{x}})=\sigma (x_i)$ and ${{\,\mathrm{lca}\,}}_T(y'_j,{\widetilde{x}})\prec _T u ={{\,\mathrm{lca}\,}}_T(y'_j,x_i)$. This implies that $(x_i,y'_j)\in E(\vec {G})$ and $(y'_j,x_i)\in E(\vec {G})$, respectively. Therefore $x_i y'_j$ is an edge in $\vec {G}$ for $1\le i<j\le k$. In summary, (H2) of in Def. 14 is always satisfied.

Hence, if $x_1$ and $y_1$ are constructed based on Case (A) and (A’), respectively, we are done.

It remains to show that *z* and $z'$ are a left and a right tail, resp., of the hourglass chain in Case (B) or (B’). First assume Case (B), and thus $z=x$. We have $z,x_1\preceq _T v_0$ by construction and $(z, x_1)\in E(\vec {G})$ as shown above. Together with $x'_1\preceq _T v_1$, this implies that ${{\,\mathrm{lca}\,}}_T(z,x_1)\preceq _T v_0 \prec _T u = {{\,\mathrm{lca}\,}}_T(z,x'_1)$. Using $\sigma (x_1)=\sigma (x'_1)$ we therefore obtain $(z,x'_1)\notin E(\vec {G})$. and hence *z* is a left tail of the constructed hourglass chain. Now assume Case (B’), and thus, $z'=y$. We have $z',y_k\preceq _T v_{k+1}$ and $(z', y_k)\in E(\vec {G})$ by construction. Together with $y'_k\preceq _T v_k$ this implies ${{\,\mathrm{lca}\,}}_T(z',y_k)\preceq _T v_{k+1} \prec _T u = {{\,\mathrm{lca}\,}}_T(z',y'_k)$. Using $\sigma (y_k)=\sigma (y'_k)$, we obtain $(z',y'_k)\notin E(\vec {G})$ and hence $z'$ is a right tail of the constructed hourglass chain.

In summary,  is an hourglass chain, possibly with left tail *z* and right tail $z'$. Furthermore, precisely one of the Conditions 1–4 in the statement holds by construction. $\square$

### Hug-edges and no-hug graphs

#### Definition 16

An edge *xy* in a vertex-colored graph $(\vec {G},\sigma )$ is a *hug-edge* if it satisfies at least one of the following conditions:

*(C1)*: *xy* is the middle edge of a good quartet in $(\vec {G},\sigma )$;
*(C2)*: *xy* is the first edge of an ugly quartet in $(\vec {G},\sigma )$; or
*(C3)*: there is an hourglass chain in $(\vec {G},\sigma )$, and one of the following cases holds: 1.: $x_1=x$ and $y_k=y$; 2.: $y_k=y$ and $z:=x$ is a left tail of ${\mathfrak {H}}$; 3.: $x_1=x$ and $z':=y$ is a right tail of ${\mathfrak {H}}$; or 4.: $z:=x$ is a left tail and $z':=y$ is a right tail of ${\mathfrak {H}}$.

The term **hug**-edge refers to the fact *xy* is a particular edge of an **h**ourglass-chain, an **u**gly quartet, or a **g**ood quartet.

#### Theorem 7

An edge *xy* in $\vec {G}(T,\sigma )$ with $u:={{\,\mathrm{lca}\,}}_T(x,y)$, $v_x, v_y\in \mathsf {child}_T(u)$, $x\preceq _T v_x$, and $y\preceq _T v_y$ is a hug-edge if $v_x$ and $v_y$ belong to the same connected component of ${\mathfrak {C}}_T(u)$. Moreover, every hug-edge is *u-fp*.

#### Proof

We show first that *xy* satisfies one of the Conditions (C1), (C2), or ((C3), and hence is hug-edge. First, note that $v_x\ne v_y$. Moreover, Lemma 4 implies $\sigma (x)\notin \sigma (L(T(v_y)))$ and $\sigma (y)\notin \sigma (L(T(v_x)))$. Since by assumption $v_x,v_y$ belong to the same connected component, there is a shortest path $P:=(v_x=v_0,\dots ,v_{k+1}=v_y)$ in ${\mathfrak {C}}_T(u)$. For $k=0$, $v_x v_y\in E({\mathfrak {C}}_T(u))$. This implies ${\mathcal {S}}^{\cap }(x,y)=\sigma (L(T(v_x)))\cap \sigma (L(T(v_y)))\ne \emptyset$. By Prop. 5, the edge *xy* is either the middle edge of a good quartet or the first edge of an ugly quartets in $(\vec {G},\sigma )$. Hence, Condition (C1) or (C2) is satisfied. If $k>0$, Lemma 19 implies Condition (C3).

For each of the three cases we have already shown that *xy* is *u-fp*: For (C1) Prop. 2 applies, for (C2) Prop. 4 provides the desired result, and for (C3) we use Lemma 17. $\square$

#### Lemma 20

If the BMG $\vec {G}(T,\sigma )$ contains a hug-edge *xy* in a BMG $\vec {G}(T,\sigma )$, then there are distinct vertices $v_1,v_2\in \mathsf {child}_{T}({{\,\mathrm{lca}\,}}_T(x,y))$ such that $\sigma (L(T(v_1)))\cap \sigma (L(T(v_2)))\ne \emptyset$.

#### Proof

Let *xy* be a hug-edge in the BMG $(\vec {G},\sigma ) = \vec {G}(T,\sigma )$, i.e. one of (C1), (C2), or (C3) applies.

If $e=xy$ satisfies (C1), then *xy* is the middle edge of a good quartet $\langle zxyz'\rangle$ in $(\vec {G},\sigma )$. By (Geiß et al. 2020c, Lemma 36), there is a vertex $u:={{\,\mathrm{lca}\,}}_{T}(x,y,z,z')$ such that $x,z\preceq _{T}v_1$ and $y,z'\preceq _{T}$ for some distinct $v_1,v_2\in \mathsf {child}_{T}(u)$. Thus, $u={{\,\mathrm{lca}\,}}_{T}(x,y)$. Moreover, since $\sigma (z)=\sigma (z')$, we have $\sigma (L(T(v_1)))\cap \sigma (L(T(v_2)))\ne \emptyset$ for two distinct vertices $v_1,v_2\in \mathsf {child}_{T}(u)$.

If $e=xy$ satisfies (C2), then it is the first edge of some ugly quartet, which w.l.o.g. has the form $\langle xyx'z\rangle$. Re-using the arguments in the proof of Prop. 4 shows that there must be two distinct children $v_1$ and $v_2$ of vertex $u={{\,\mathrm{lca}\,}}_{T}(x,y)$ such that $\sigma (L(T(v_1)))\cap \sigma (L(T(v_2)))\ne \emptyset$.

If $e=xy$ satisfies (C3), then there is a (tailed) hourglass chain , $k\ge 1$, in $\vec {G}(T,\sigma )$, such that either $x=x_1$ or $z:=x$ is a left tail of ${\mathfrak {H}}$, and either $y=y_k$ or $z':=y$ is a right tail of ${\mathfrak {H}}$. In either case, Lemma 16 implies $x\preceq _{T} v_0$ and $y\preceq _{T} v_{k+1}$. Since $x_1$ and $x'_1$ lie below distinct children $v_0$ and $v_1$ of vertex ${{\,\mathrm{lca}\,}}_T(x,y)$ and $\sigma (x_1)=\sigma (x'_1)$ by the definition of hourglasses, it holds that $\sigma (L(T(v_0)))\cap \sigma (L(T(v_1)))\ne \emptyset$.

In each case, therefore, there are distinct vertices $v_1,v_2\in \mathsf {child}_{T}({{\,\mathrm{lca}\,}}_T(x,y))$ such that $\sigma (L(T(v_1)))\cap \sigma (L(T(v_2)))\ne \emptyset$. $\square$

The fact that all hug-edges are *u-fp* by Thm. 7 suggests to consider the subgraph of a BMG that is left after removing all these unambiguously recognizable false-positive orthology assignments.

#### Definition 17

Let $(\vec {G},\sigma )$ be a BMG with symmetric part *G* and let *F* be the set of its hug-edges. The *no-hug* graph ${\mathbb {N}}{\mathbb {H}}(\vec {G},\sigma )$ is the subgraph of *G* with vertex set $V(\vec {G})$, coloring $\sigma$ and edge set $E(G){\setminus } F$.

The ${\mathbb {N}}{\mathbb {H}}(\vec {G},\sigma )$ is therefore the subgraph of the underlying RBMG of $\vec {G}$ that contains all edges that cannot be identified as *u-fp* by using only good quartets, ugly quartets and (tailed) hourglass chains as outlined in Thm. 7.

#### Corollary 5

Let $(T,\sigma )$ be a leaf-colored tree and $\mu$ a reconciliation map from $(T,\sigma )$ to some species tree *S*. Then,

$$\begin{aligned} \Theta (T,t_{\mu }) \subseteq \Theta (T, {{\widehat{t}}_T}) \subseteq {\mathbb {N}}{\mathbb {H}}(\vec {G}(T,\sigma )) \subseteq \vec {G}(T,\sigma ). \end{aligned}$$

#### Proof

By Thm. 2, $\Theta (T,t_{\mu }) \subseteq \Theta (T,{{\widehat{t}}_T}) \subseteq \vec {G}(T,\sigma )$; and by definition, we have ${\mathbb {N}}{\mathbb {H}}(\vec {G}(T,\sigma )) \subseteq \vec {G}(T,\sigma )$. Now, let *xy* be an edge in $\Theta (T,{{\widehat{t}}_T})$ and thus, . By definition of ${{\widehat{t}}_T}$, we have $\sigma (L(T(v_1))) \cap \sigma (L(T(v_2)))=\emptyset$ for any two distinct $v_1,v_2\in \mathsf {child}_T({{\,\mathrm{lca}\,}}_T(x,y))$. The contraposition of Lemma 20 implies that *xy* is not a hug-edge and thus an edge of ${\mathbb {N}}{\mathbb {H}}(\vec {G}(T,\sigma ))$, which completes the proof. $\square$

The no-hug graph still may contain false-positive orthology assignments, i.e., ${\mathbb {N}}{\mathbb {H}}(\vec {G}(T,\sigma ))=\Theta (T,t_{\mu })$ does not hold in general. In the following section, we shall see that there are, however, no *u-fp* edges left in the no-hug graph.

### Resolving least resolved trees

Since every BMG $(\vec {G},\sigma )$ at least implicitly contains all information needed to identify its *u-fp* edges, this is also true for its unique least resolved tree $(T^*,\sigma )$. It is not always possible, however, to assign an event labeling *t* to $T^*$ such that $(T^*,t)$ is the cotree for the correct orthology relation. Fig. 7 shows that $T^*$ may not be “resolved enough”. To tackle this problem, we analyze the redundant edges of more resolved trees that explain $(\vec {G},\sigma )$. Cor. 1 implies that all edges below a speciation vertex are redundant because, by Lemma 2, the color sets of distinct subtrees below a speciation vertex do not overlap. More precisely, we have

#### Observation 8

Let $\mu$ be a reconciliation map from $(T,\sigma )$ to *S* and assume that there is a vertex $u\in V^0(T)$ such that $\mu (u)\in V^0(S)$ and thus, $t_{\mu }(u)=\newmoon$. Then every inner edge *uv* of *T* with $v\in \mathsf {child}_{T}(u)$ is redundant w.r.t. $\vec {G}(T,\sigma )$. Moreover, if an inner edge *uv* with $v\in \mathsf {child}_{T}(u)$ is non-redundant, then *u* must have two children with overlapping color sets, and hence, $t_{\mu }(u)=\square$.

To identify the vertices in $(T^*,\sigma )$ that can be expanded to yield a tree that still explains $\vec {G}(T^*,\sigma )$, we introduce a particular way of “augmenting” a leaf-colored tree.

#### Definition 18

Let $(T,\sigma )$ be a leaf-colored tree, *u* be an inner vertex of *T*, ${\mathfrak {C}}_T(u)$ the corresponding color-set intersection graph, and ${\mathcal {C}}$ the set of connected components of ${\mathfrak {C}}_T(u)$. Then the tree $T_u$ *augmented at vertex* *u* is obtained by applying the following editing steps to *T*:

- If ${\mathfrak {C}}_T(u)$ is connected, do nothing.
- Otherwise, for each $C\in {\mathcal {C}}$ with $|C|>1$
  - introduce a vertex *w* and attach it as a child of *u*, i.e., add the edge *uw*,
  - for every element $v_i\in C$, substitute the edge $uv_i$ by the edge $wv_i$.

The augmentation step is *trivial* if $T_u=T$, in which case we say that *no edit step was performed*.

An example of an augmentation is shown in Fig. 8. It is easy to see that the tree $T_u$ obtained by an augmentation of a phylogenetic tree *T* is again a phylogenetic tree. The augmentation step at vertex *u* of *T* is trivial if and only if either ${\mathfrak {C}}_T(u)$ is connected or all connected components $C\in {\mathcal {C}}$ are singletons, i.e., $|C|=1$. If $(T_u,\sigma )$ is obtained by augmenting $(T,\sigma )$ at node *u*, we denote the set of newly introduced vertices by $V_{\lnot T}:=V(T_u){\setminus } V(T)$. Note that $V_{\lnot T}=\emptyset$ whenever no edit step was performed.

Since augmentation only inserts vertices between *u* and its children, it affects neither *L*(*T*(*u*)) nor *L*(*T*(*v*)) for $v\in \mathsf {child}(u)$. As an immediate consequence we find

#### Observation 9

Let $(T,\sigma )$ be a leaf-colored tree, $u\ne v$ two inner vertices of *T*, ${\mathfrak {C}}_T(u)$ the corresponding color-set intersection graph, and $(T_u,\sigma )$ the tree obtained by augmenting *T* at *u*. Then ${\mathfrak {C}}_{T_u}(v)={\mathfrak {C}}_{T}(v)$.

#### Lemma 21

Let $(T,\sigma )$ be a leaf-colored tree. Let $u\in V^0(T)$ and $T_u$ be the tree after augmenting *T* at vertex *u*. If ${\mathfrak {C}}_T(u)$ is disconnected, then $\sigma (L(T_u(w_1)))\cap \sigma (L(T_u(w_2)))=\emptyset$ for any two distinct vertices $w_1,w_2\in \mathsf {child}_{T_u}(u)$.

#### Proof

By construction, the vertex $w_i$ in $T_u$, $i=1,2$, is either a child of *u* in *T* or was inserted in the augmentation step. Therefore, the two connected components $C_1$ and $C_2$ of ${\mathfrak {C}}_T(u)$ to which $w_1$ and $w_2$ belong are disjoint. Thus $\sigma (L(T(v_i)))\cap \sigma (L(T(v_j)))= \emptyset$ for all $v_i,v_j\in \mathsf {child}_T(u)$ with $v_i\in C_1$ and $v_j\in C_2$ because otherwise there would be an edge $v_iv_j$ in ${\mathfrak {C}}_T(u)$ and thus, $C_1=C_2$. Since $w_i$ is either the single vertex in $C_i$ or $w_i$ has as children the vertices of $C_i$ in $T_u$, $i\in \{1,2\}$, we conclude that $\sigma (L(T_u(w_1)))\cap \sigma (L(T_u(w_2)))=\emptyset$. $\square$

The following result shows that no further edit step can be performed at vertices that have been newly introduced by a previous augmentation step or have already undergone an augmentation.

#### Lemma 22

Let $(T,\sigma )$ be a leaf-colored tree, $u\in V^0(T)$, $(T_u,\sigma )$ the tree obtained by augmenting *T* at *u*, and denote by $(T_{uw},\sigma )$ the tree obtained by augmenting $T_u$ at *w*. Then $T_{uw}=T_u$ for $w=u$ as well as for all newly introduced vertices, i.e., for all $w\in V_{\lnot T}\cup \{u\}$.

#### Proof

If $T_u=T$, then $V_{\lnot T}=\emptyset$ and thus $T_{uu}=T_{u}=T$. If $T_u\ne T$, then the definition of the augmentation step at *u* implies that either ${\mathfrak {C}}_{T_u}(u)$ is connected or all connected components of ${\mathfrak {C}}_{T_u}(u)$ are singletons. In either case Lemma 21 ensured that augmentation at *u* leaves $T_{u}$ unchanged, i.e., $T_{uu}=T_u$. By construction, ${\mathfrak {C}}_{T_u}(w)$ is connected for $w\in V_{\lnot T}{\setminus }\{u\}$ and thus, we have $T_{uw}=T_{u}$. $\square$

The tree obtained by augmenting a set of inner vertices of $(T,\sigma )$ is therefore independent of the order of the augmentation steps.

#### Definition 19

*(Augmented tree)* Let $(T,\sigma )$ be a leaf-colored tree. The *augmented tree of* $(T,\sigma )$, denoted by $({{\,\mathrm{{\mathcal {A}}}\,}}(T),\sigma )$, is obtained by augmenting all inner vertices of $(T,\sigma )$.

#### Lemma 23

For every leaf-colored tree $(T,\sigma )$ there is a unique tree $({{\,\mathrm{{\mathcal {A}}}\,}}(T),\sigma )$ obtained from $(T,\sigma )$ by repeated application of augmentation steps until only trivial augmentation steps remain. The tree $({{\,\mathrm{{\mathcal {A}}}\,}}(T),\sigma )$ is computed by Alg. 1.

#### Proof

Lemma 22 together with Obs. 9 implies that (i) every vertex *u* in *T* can be non-trivially augmented at most once, (ii) the newly introduced vertices cannot be non-trivially augmented at all, and (iii) augmentation of two distinct inner vertices of *T* yields the same result irrespective of the order of the augmentation steps. Thus, $({{\,\mathrm{{\mathcal {A}}}\,}}(T),\sigma )$ is unique. The correctness of Alg. 1 now follows immediately. $\square$

#### Lemma 24

Alg. 1 with input $T=(V,E)$ and $\sigma$ runs in $O(|V|^2|{\mathscr {S}}|)$ time and $O(|V|^2)$ space, where ${\mathscr {S}} = \sigma (L(T))$ is the set of species under consideration.

#### Proof

Assigning the color set *L*(*T*(*u*)) to each *u* requires $O(|V| |{\mathscr {S}}|)$ time, where $|{\mathscr {S}}|<|V|$. The total effort to construct all ${\mathfrak {C}}_T(u)$ is bounded by $O(|V|^2|{\mathscr {S}}|)$, corresponding to comparing the color sets of all pairs of vertices of *T*. The total size of all color-set intersection graphs in $O(|V|^2)$. Computation of the connected components is linear in the size of the graph, which also bounds the editing effort for each *u*, implying the claim. $\square$

We finally show that augmentation does not affect the underlying BMG.

#### Proposition 7

For every leaf-colored tree $(T,\sigma )$, it holds $\vec {G}(T,\sigma )=\vec {G}({{\,\mathrm{{\mathcal {A}}}\,}}(T),\sigma )$.

#### Proof

Let $u\in V^0(T)$ and $T_u$ be the tree after augmenting *T* at vertex *u*. Put $A:=\{uw\mid w\in V_{\lnot T}\}$ and note that all edges of $T_u$ in *A* are inner edges. Now consider $e\in A$. Since $w\in V_{\lnot T}$, an edit step was performed to obtain *w* and thus, $|{\mathcal {C}}|>1$ in ${\mathfrak {C}}_T(u)$. Lemma 21 and $|{\mathcal {C}}|>1$ imply that for any $v'\in \mathsf {child}_{T_u}(u)$ with $v'\ne w$ we have $\sigma (L(T_u(v')))\cap \sigma (L(T_u(w)))=\emptyset$. Thus, Cor. 1 implies that the edge *uw* is redundant in $(T_u,\sigma )$ w.r.t. $\vec {G}(T,\sigma )$.

Denoting by $T_{u_A}$ the tree obtained from $T_u$ by contraction of all edges in *A*, we obtain $(T,\sigma ) = (T_{u_A},\sigma )$. Lemma 9 now implies $\vec {G}(T_u,\sigma )=\vec {G}(T_{u_A},\sigma )=\vec {G}(T,\sigma )$ for every augmentation step. By Lemma 23, we can repeat this argument for every augmentation in the arbitrary order in which $\vec {G}({{\,\mathrm{{\mathcal {A}}}\,}}(T),\sigma )$ is obtained from $\vec {G}(T,\sigma )$, and thus $\vec {G}({{\,\mathrm{{\mathcal {A}}}\,}}(T),\sigma )=\vec {G}(T,\sigma )$. $\square$

### Extremal labeling of augmented trees

While the least resolved tree in general cannot support an event labeling that properly reflects the underlying true history of a gene family, we shall see here that the augmented tree $({{\,\mathrm{{\mathcal {A}}}\,}}(T),\sigma )$ does feature sufficient resolution. To this end, we investigate the extremal event labeling of $({{\,\mathrm{{\mathcal {A}}}\,}}(T),\sigma )$.

#### Lemma 25

Let ${{\widehat{t}}}:={{\widehat{t}}_{{{\,\mathrm{{\mathcal {A}}}\,}}(T)}}$ be the extremal event labeling of the augmented tree $({{\,\mathrm{{\mathcal {A}}}\,}}(T),\sigma )$ obtained from $(T,\sigma )$ and let *u* be some vertex of ${{\,\mathrm{{\mathcal {A}}}\,}}(T)$. Then it holds ${{\widehat{t}}}(u)=\square$ if and only if ${\mathfrak {C}}_{{{\,\mathrm{{\mathcal {A}}}\,}}(T)}(u)$ is connected.

#### Proof

By the definitions of the extremal event labeling and ${\mathfrak {C}}_{{{\,\mathrm{{\mathcal {A}}}\,}}(T)}(u)$, the ‘if’-direction is clear. Now suppose that ${{\widehat{t}}}(u)=\square$. There are two possibilities:

(1) $u\in V^0(T)$. If ${\mathfrak {C}}_T(u)$ is connected, then ${\mathfrak {C}}_{{{\,\mathrm{{\mathcal {A}}}\,}}(T)}(u)={\mathfrak {C}}_T(u)$. Otherwise, Lemma 21 implies that $\sigma (L({{\,\mathrm{{\mathcal {A}}}\,}}(T)(w_1)))\cap \sigma (L({{\,\mathrm{{\mathcal {A}}}\,}}(T)(w_2))) = \emptyset$ for all $w_1,w_2\in \mathsf {child}_{{{\,\mathrm{{\mathcal {A}}}\,}}(T)}(u)$, thus the definition of the extremal event labeling implies ${{\widehat{t}}}(u)\ne \square$, a contradiction.

(2) $u\in V_{\lnot T}$, i.e., *u* is newly created by augmenting some $u'\in V^0(T)$, hence ${\mathfrak {C}}_{T}(u)$ is connected and, by Obs. 9 and Lemma 22, ${\mathfrak {C}}_{{{\,\mathrm{{\mathcal {A}}}\,}}(T)}(u)$ is connected. $\square$

For later reference, we need the following

#### Lemma 26

Let $(\vec {G},\sigma )$ be a BMG, $(T^*,\sigma )$ its least resolved tree, and ${{\widehat{t}}}:={{\widehat{t}}_{{{\,\mathrm{{\mathcal {A}}}\,}}(T^*)}}$ the extremal event labeling of the augmented tree $({{\,\mathrm{{\mathcal {A}}}\,}}(T^*),\sigma )$. Then, $({{\,\mathrm{{\mathcal {A}}}\,}}(T^*),{{\widehat{t}}},\sigma )$ does not contain adjacent speciation vertices, i.e., if ${{\widehat{t}}}(u)=\newmoon$ for a vertex *u* of ${{\,\mathrm{{\mathcal {A}}}\,}}(T^*)$, then ${{\widehat{t}}}(v)=\square$ for any of its non-leaf children $v\in \mathsf {child}_{{{\,\mathrm{{\mathcal {A}}}\,}}(T^*)}(u){\setminus } L({{\,\mathrm{{\mathcal {A}}}\,}}(T^*))$.

#### Proof

Set ${{\,\mathrm{{\mathcal {A}}}\,}}:={{\,\mathrm{{\mathcal {A}}}\,}}(T^*)$ and note that, by Prop. 7, $({{\,\mathrm{{\mathcal {A}}}\,}},\sigma )$ explains $(\vec {G},\sigma )$. Assume, for contradiction, that there is an inner edge *uv* in ${{\,\mathrm{{\mathcal {A}}}\,}}$ with $v\prec _{{{\,\mathrm{{\mathcal {A}}}\,}}} u$ such that ${{\widehat{t}}}(u)={{\widehat{t}}}(v)=\newmoon$. By the definition of the extremal event labeling ${{\widehat{t}}}$, we have $\sigma (L({{\,\mathrm{{\mathcal {A}}}\,}}(v)))\cap \sigma (L({{\,\mathrm{{\mathcal {A}}}\,}}(v')))=\emptyset$ for any $v'\in \mathsf {child}_{{{\,\mathrm{{\mathcal {A}}}\,}}}(u){\setminus }\{v\}$. Together with Cor. 1 this implies that *uv* is redundant for $(\vec {G},\sigma )$, and hence, not an edge in the least resolved tree $(T^*,\sigma )$. Now consider the augmentation in which the edge *uv*, and thus vertex *v* was created; resulting in a tree $(T',\sigma )$. By the definition of augmenting (Def. 18), it clearly holds that ${\mathfrak {C}}_{T'}(v)$ is connected. By Lemma 22, the edges adjacent to *v* do not change in any subsequent augmentation. Thus ${\mathfrak {C}}_{{{\,\mathrm{{\mathcal {A}}}\,}}}(v)$ must be connected as well. Lemma 25 now implies that ${{\widehat{t}}}(v)=\square$; a contradiction. $\square$

#### Lemma 27

Let $(\vec {G},\sigma )$ be a BMG and $(T^*,\sigma )$ its unique least resolved tree. Moreover, let ${{\widehat{t}}}:={{\widehat{t}}_{{{\,\mathrm{{\mathcal {A}}}\,}}(T^*)}}$ be the extremal event labeling of the augmented tree $({{\,\mathrm{{\mathcal {A}}}\,}}(T^*),\sigma )$. Then, $\Theta ({{\,\mathrm{{\mathcal {A}}}\,}}(T^*),{{\widehat{t}}}) \subseteq \vec {G}$.

#### Proof

Since $(T^*,\sigma )$ explains $(\vec {G},\sigma )$, we have $(\vec {G},\sigma ) = \vec {G}(T^*,\sigma )$. By Prop. 7, we have $\vec {G}(T^*,\sigma )=\vec {G}({{\,\mathrm{{\mathcal {A}}}\,}}(T^*),\sigma )$. Let *xy* be an edge in $\Theta ({{\,\mathrm{{\mathcal {A}}}\,}}(T^*),{{\widehat{t}}})$. By definition, ${{\widehat{t}}}({{\,\mathrm{lca}\,}}_{{{\,\mathrm{{\mathcal {A}}}\,}}(T^*)}(u))=\newmoon$ where $u:={{\,\mathrm{lca}\,}}_{{{\,\mathrm{{\mathcal {A}}}\,}}(T^*)}(x,y)$. By definition of the extremal event labeling, $\sigma (L({{\,\mathrm{{\mathcal {A}}}\,}}(T^*)(v_1)))\cap \sigma (L({{\,\mathrm{{\mathcal {A}}}\,}}(T^*)(v_2)))=\emptyset$ for all two distinct vertices $v_1,v_2\in \mathsf {child}_{{{\,\mathrm{{\mathcal {A}}}\,}}(T^*)}(u)$. The latter is true, in particular, for the two children $v_x,v_y\in \mathsf {child}_{{{\,\mathrm{{\mathcal {A}}}\,}}(T^*)}(u)$ with $x\preceq _{{{\,\mathrm{{\mathcal {A}}}\,}}(T^*)} v_x$ and $y\preceq _{{{\,\mathrm{{\mathcal {A}}}\,}}(T^*)} v_y$. Therefore, $\sigma (x)\notin \sigma (L({{\,\mathrm{{\mathcal {A}}}\,}}(T^*)(v_y)))$ and $\sigma (y)\notin \sigma (L({{\,\mathrm{{\mathcal {A}}}\,}}(T^*)(v_x)))$. We conclude that *x* and *y* are reciprocal best matches in ${{\,\mathrm{{\mathcal {A}}}\,}}(T^*)$. Finally, $(\vec {G},\sigma ) =\vec {G}({{\,\mathrm{{\mathcal {A}}}\,}}(T^*),\sigma )$ implies that *xy* is an edge in $\vec {G}$. $\square$

Now we are in the position to prove the main results of this contribution.

#### Theorem 10

Let $(\vec {G},\sigma )$ be a BMG, $(T^*,\sigma )$ its unique least resolved tree, and ${{\widehat{t}}}:={{\widehat{t}}_{{{\,\mathrm{{\mathcal {A}}}\,}}(T^*)}}$ the extremal event labeling of the augmented tree $({{\,\mathrm{{\mathcal {A}}}\,}}(T^*),\sigma )$. Then $(\Theta ({{\,\mathrm{{\mathcal {A}}}\,}}(T^*),{{\widehat{t}}}),\sigma ) = {\mathbb {N}}{\mathbb {H}}(\vec {G},\sigma )$.

#### Proof

Let $(G,\sigma )$ be the symmetric part of $(\vec {G}=(V,E),\sigma )$. For simplicity, we write $G_{\Theta } :=\Theta ({{\,\mathrm{{\mathcal {A}}}\,}}(T^*),{{\widehat{t}}})$ and $G_{{\mathbb {N}}{\mathbb {H}}} :=(V, E({\mathbb {N}}{\mathbb {H}}(\vec {G},\sigma )))$. Recall that, by definition, $G_{{\mathbb {N}}{\mathbb {H}}}\subseteq G$ and, by Lemma 27, $G_{\Theta }\subseteq \vec {G}$. Finally, as *G* contains only edges of $\vec {G}$, we have $G_{\Theta }\subseteq G$. Let $F :=E(G) {\setminus } E(G_{{\mathbb {N}}{\mathbb {H}}})$ be the set of all edges of *G* that are hug-edges, and let $F' :=E(G){\setminus } E(G_{\Theta })$ be the set of all edges in *G* that do not form orthologous pairs. Since $G_{{\mathbb {N}}{\mathbb {H}}},G_{\Theta }\subseteq G$ it suffices to verify that $F=F'$ in order to show that $(G_{\Theta },\sigma )=(G_{{\mathbb {N}}{\mathbb {H}}},\sigma )$.

Assume $e=xy\in F'$. Hence, $xy\notin E(G_{\Theta })$ and therefore, ${{\widehat{t}}}(u)=\square$ where $u:={{\,\mathrm{lca}\,}}_{{{\,\mathrm{{\mathcal {A}}}\,}}(T^*)}(x,y)$. By Lemma 25, ${\mathfrak {C}}_{{{\,\mathrm{{\mathcal {A}}}\,}}(T^*)}(u)$ has exactly one connected component. This together with Thm. 7 implies that *xy* is a hug-edge and thus, $xy\in F$, and hence $F'\subseteq F$.

Assume $e=xy\in F$ is a hug-edge. Assume, for contradiction, that $e\notin F'$ and thus, ${{\widehat{t}}}(u)=\newmoon$ where $u:={{\,\mathrm{lca}\,}}_{{{\,\mathrm{{\mathcal {A}}}\,}}(T^*)}(x,y)$. By definition of the extremal event labeling, it must therefore hold that $\sigma (L({{\,\mathrm{{\mathcal {A}}}\,}}(T^*)(v_1)))\cap \sigma (L({{\,\mathrm{{\mathcal {A}}}\,}}(T^*)(v_2)))=\emptyset$ for any two distinct vertices $v_1,v_2\in \mathsf {child}_{{{\,\mathrm{{\mathcal {A}}}\,}}(T^*)}(u)$. By Prop. 7, $({{\,\mathrm{{\mathcal {A}}}\,}}(T^*),\sigma )$ explains $(\vec {G},\sigma )$. This together with Lemma 20 implies that there are two distinct vertices $v_1,v_2\in \mathsf {child}_{{{\,\mathrm{{\mathcal {A}}}\,}}(T^*)}(u)$ such that $\sigma (L({{\,\mathrm{{\mathcal {A}}}\,}}(T^*)(v_1)))\cap \sigma (L({{\,\mathrm{{\mathcal {A}}}\,}}(T^*)(v_2)))\ne \emptyset$; a contradiction. Therefore, $e\in F'$, and hence $F\subseteq F'$. $\square$

#### Theorem 11

An edge *xy* in a BMG $(\vec {G},\sigma )$ is *u-fp* if and only if *xy* is a hug-edge of $(\vec {G},\sigma )$.

#### Proof

Let $(\vec {G},\sigma )$ be a BMG, $(T^*,\sigma )$ its unique least resolved tree, and ${{\widehat{t}}}:={{\widehat{t}}_{{{\,\mathrm{{\mathcal {A}}}\,}}(T^*)}}$ the extremal event labeling of the augmented tree $({{\,\mathrm{{\mathcal {A}}}\,}}(T^*),\sigma )$. As shown in the proof of Thm. 10, every edge *xy* of of the symmetric part *G* that is not a hug-edge satisfies $xy\in E(G_{\Theta })$ and therefore ${{\widehat{t}}}(u)=\newmoon$, where $u:={{\,\mathrm{lca}\,}}_{{{\,\mathrm{{\mathcal {A}}}\,}}(T^*)}(x,y)$. Lemma 10 implies that *e* is not $({{\,\mathrm{{\mathcal {A}}}\,}}(T^*),\sigma )$-*fp* and thus, in particular, not *u-fp*. That is, all edges in $(G_{\Theta },\sigma )=(G_{{\mathbb {N}}{\mathbb {H}}},\sigma )$ are non-*u-fp* edges. Moreover, Thm. 7 implies that all hug-edges in $E(G) {\setminus } E(G_{{\mathbb {N}}{\mathbb {H}}})$ are *u-fp*. Since $(G_{{\mathbb {N}}{\mathbb {H}}},\sigma )$ does not contain *u-fp* edges, all *u-fp* edges must also be hug-edges, which completes the proof. $\square$

We next show that ${\mathbb {N}}{\mathbb {H}}(\vec {G},\sigma )$ can be computed in polynomial time. In fact, the effort is dominated by computing the least resolved tree $(T^*,\sigma )$ for a given BMG.

#### Theorem 12

For a given BMG $(\vec {G},\sigma )$, the set of all *u-fp* edges can be computed in $O(|L|^3 |{\mathscr {S}}|)$ time, where $L=V(\vec {G})$ and ${\mathscr {S}} = \sigma (L(T))$ is the set of species under consideration.

#### Proof

Given a BMG $(\vec {G},\sigma )$, its least resolved tree $(T^*,\sigma )$ can be computed in $O(|L|^3 |{\mathscr {S}}|)$ time (cf. Thm. 3 and (Geiß et al. 2019, Sec. 5)). The augmented tree $({{\,\mathrm{{\mathcal {A}}}\,}}(T^*),\sigma )$ can be obtained from $(T^*,\sigma )$ in $O(|L|^2 |{\mathscr {S}}|)$ time according to Lemma 24. The extremal event labeling ${{\widehat{t}}}$ can be obtained from the connectivity information on the ${\mathfrak {C}}_{{{\,\mathrm{{\mathcal {A}}}\,}}(T^*)}(u)$ in linear time. Computing $(\Theta ({{\,\mathrm{{\mathcal {A}}}\,}}(T^*),{{\widehat{t}}}),\sigma ) = {\mathbb {N}}{\mathbb {H}}(\vec {G},\sigma )$ then only requires evaluation of ${{\,\mathrm{lca}\,}}_{{{\,\mathrm{{\mathcal {A}}}\,}}(T^*)}(x,y)$, which can be achieved in polynomial time in $O(|L|^2)$ as described in (Geiß et al. 2019, Sec. 5)). $\square$

### Additional unidentified false-positives

For an event-labeled, leaf-colored tree $(T,t,\sigma )$, we consider the triple set

3 $$\begin{aligned} \begin{aligned} {\mathfrak {S}}(T,t,\sigma ) = \{\sigma (a)\sigma (b)|\sigma (c) :&ab|c\le T;\; t({{\,\mathrm{lca}\,}}_T(a,b,c))=\newmoon ; \\&\sigma (a),\sigma (b),\sigma (c) \text { pairwise distinct} \}. \end{aligned} \end{aligned}$$

Moreover, we will need the following characterization of biologically plausible event-labeled gene trees:

#### Theorem 13

Hernandez-Rosales et al. (2012), Hellmuth (2017) There is a species tree *S* together with a reconciliation map $\mu$ from $(T,t,\sigma )$ to *S* such that $t_{\mu }=t$ if and only if ${\mathfrak {S}} (T,t,\sigma )$ is compatible. In this case, every species tree *S* that displays ${\mathfrak {S}}(T,t,\sigma )$ can be reconciled with $(T,t,\sigma )$. Moreover, there is a polynomial-time algorithm that determines whether a species tree for $(T,t, \sigma )$ exists, and if so, returns a species tree *S* together with a reconciliation map $\mu :T\rightarrow S$.

Throughout this section we are only concerned with the extremal event labeling ${{\widehat{t}}_{{{\,\mathrm{{\mathcal {A}}}\,}}(T^*)}}$ of the augmented trees $({{\,\mathrm{{\mathcal {A}}}\,}}(T^*),\sigma )$ of least resolved trees $(T^*,\sigma )$. For brevity, we simply write ${{\widehat{t}}}$. For a BMG $(\vec {G},\sigma )$, we consider the set of trees

4 $$\begin{aligned} {\mathfrak {T}} :=\left\{ (T,t,\sigma ) \;|\; {\mathbb {N}}{\mathbb {H}}(\vec {G},\sigma ) = (\Theta (T,t),\sigma )\right\} . \end{aligned}$$

An orthology relation ${\mathbb {N}}{\mathbb {H}}(\vec {G},\sigma )$ obtained from a BMG $(\vec {G},\sigma )$ by removing all of its *u-fp* edges is biologically feasible only if there is an event-labeled gene tree $(T,t,\sigma )\in {\mathfrak {T}}$ that can be reconciled with some species tree. To show that this condition can be tested in polynomial time, we first need a technical result.

#### Lemma 28

Let $(\vec {G},\sigma )$ be a BMG with LRT $(T^*,\sigma )$, and let ${\mathfrak {T}}$ be be given by Eq. (4). If *ab*|*c* is displayed by ${{\,\mathrm{{\mathcal {A}}}\,}}(T^*)$ and ${{\widehat{t}}}({{\,\mathrm{lca}\,}}_{{{\,\mathrm{{\mathcal {A}}}\,}}(T^*)}(a,b,c)) = \newmoon$, then *ab*|*c* is also displayed by every tree $(T,t,\sigma )\in {\mathfrak {T}}$ and $t({{\,\mathrm{lca}\,}}_{T}(a,b,c))=\newmoon$.

#### Proof

Suppose that *ab*|*c* is displayed by ${{\,\mathrm{{\mathcal {A}}}\,}}(T^*)$ and ${{\widehat{t}}}({{\,\mathrm{lca}\,}}_{{{\,\mathrm{{\mathcal {A}}}\,}}(T^*)}(a,b,c)) = \newmoon$. Thm. 10 implies $(\Theta ({{\,\mathrm{{\mathcal {A}}}\,}}(T^*),{{\widehat{t}}}),\sigma )={\mathbb {N}}{\mathbb {H}}(\vec {G},\sigma )$. Thus ${\mathbb {N}}{\mathbb {H}}(\vec {G},\sigma )$ is a cograph by Thm. 1. Let $(T',t',\sigma )$ be a least resolved tree for the cograph ${\mathbb {N}}{\mathbb {H}}(\vec {G},\sigma )$. Clearly, $(T',t',\sigma )\in {\mathfrak {T}}$. This tree is unique and any other tree in ${\mathfrak {T}}$ must be a refinement of $(T',t',\sigma )$ Corneil et al. (1981), Böcker and Dress (1998). We proceed with showing that (1) $t'({{\,\mathrm{lca}\,}}_{T'}(a,b,c)) = \newmoon$ and (2) *ab*|*c* is displayed by $T'$.

In order to show (1), assume for contradiction that $t'({{\,\mathrm{lca}\,}}_{T'}(a,b,c)) = \square$ and note that $(T',t',\sigma )\in {\mathfrak {T}}$ implies ${\mathbb {N}}{\mathbb {H}}(\vec {G},\sigma ) = (\Theta (T',t'),\sigma )$. Since ${{\widehat{t}}}({{\,\mathrm{lca}\,}}_{{{\,\mathrm{{\mathcal {A}}}\,}}(T^*)}(a,b,c)) = \newmoon$ and $ab|c \le {{\,\mathrm{{\mathcal {A}}}\,}}(T^*)$, the induced subgraph of ${\mathbb {N}}{\mathbb {H}}(\vec {G},\sigma )$ on $\{a,b,c\}$ contains at least the two edges *ac* and *bc*. However, if $t'({{\,\mathrm{lca}\,}}_{T'}(a,b,c)) = \square$, then this induced subgraph can contain at most one edge; a contradiction. Hence, $t'({{\,\mathrm{lca}\,}}_{T'}(a,b,c)) = \newmoon$.

Next, we show (2). Since ${{\,\mathrm{{\mathcal {A}}}\,}}(T^*)$ displays *ab*|*c* and $T'$ is obtained from ${{\,\mathrm{{\mathcal {A}}}\,}}(T^*)$ by a series of edge contractions, $T'$ can neither display *ac*|*b* nor *bc*|*a*, thus either $ab|c\le T'$ or ${{\,\mathrm{lca}\,}}_{T'}(a,b)={{\,\mathrm{lca}\,}}_{T'}(a,b,c)$. By Lemma 26, $({{\,\mathrm{{\mathcal {A}}}\,}}(T^*),{{\widehat{t}}})$ does not contain adjacent (consecutive) speciation vertices. Therefore and since ${{\,\mathrm{{\mathcal {A}}}\,}}(T^*)$ displays *ab*|*c*, the path from ${{\,\mathrm{lca}\,}}_{{{\,\mathrm{{\mathcal {A}}}\,}}(T^*)}(a,b,c)$ to ${{\,\mathrm{lca}\,}}_{{{\,\mathrm{{\mathcal {A}}}\,}}(T^*)}(a,b)$ in ${{\,\mathrm{{\mathcal {A}}}\,}}(T^*)$ must contain at least one duplication vertex. Since $T'$ can be obtained from ${{\,\mathrm{{\mathcal {A}}}\,}}(T^*)$ by contracting all edges *uv* in ${{\,\mathrm{{\mathcal {A}}}\,}}(T^*)$ with ${{\widehat{t}}}(u)={{\widehat{t}}}(v)$ Corneil et al. (1981), Böcker and Dress (1998), the path from ${{\,\mathrm{lca}\,}}_{T'}(a,b,c)$ to ${{\,\mathrm{lca}\,}}_{T'}(a,b)$ in $T'$ must contain at least one duplication vertex. Together with $t'({{\,\mathrm{lca}\,}}_{T'}(a,b,c)) = \newmoon$ this implies ${{\,\mathrm{lca}\,}}_{T'}(a,b)\ne {{\,\mathrm{lca}\,}}_{T'}(a,b,c)$, and hence, *ab*|*c* is displayed by $T'$.

Since every tree $(T,t,\sigma )\in {\mathfrak {T}}$ is a refinement of $(T',t',\sigma )$, the triple *ab*|*c* is also displayed by *T*. Finally, since ${\mathbb {N}}{\mathbb {H}}(\vec {G},\sigma ) = (\Theta (T,t),\sigma )$ for every tree $(T,t,\sigma )\in {\mathfrak {T}}$, we can re-use the arguments from the proof of Statement (1) to conclude that $t({{\,\mathrm{lca}\,}}_{T}(a,b,c))=\newmoon$. $\square$

#### Lemma 29

Let $(\vec {G},\sigma )$ be a BMG with LRT $(T^*,\sigma )$ and let ${\mathfrak {T}}$ be given by Eq. (4). Then, the following statements are equivalent:

1. There is no reconciliation map $\mu$ from $({{\,\mathrm{{\mathcal {A}}}\,}}(T^*),{{\widehat{t}}},\sigma )$ to any species tree such that $t_{\mu }={{\widehat{t}}}$.
2. For all trees $(T,t,\sigma )$ in ${\mathfrak {T}}$ there is no reconciliation map $\mu$ from $(T,t,\sigma )$ to any species tree such that $t_{\mu }=t$.

In particular, Condition (1) can be verified in polynomial time.

#### Proof

First note that $({{\,\mathrm{{\mathcal {A}}}\,}}(T^*),{{\widehat{t}}},\sigma )\in {\mathfrak {T}}$ since, by Thm. 10, $(\Theta ({{\,\mathrm{{\mathcal {A}}}\,}}(T^*),{{\widehat{t}}}),\sigma ) ={\mathbb {N}}{\mathbb {H}}(\vec {G},\sigma )$. Hence, Statement (2) implies (1).

For the converse, let *ab*|*c* be displayed by ${{\,\mathrm{{\mathcal {A}}}\,}}(T^*)$ where $\sigma (a)=A$, $\sigma (b)=B$, $\sigma (c)=C$ are pairwise distinct, and ${{\widehat{t}}}({{\,\mathrm{lca}\,}}_{{{\,\mathrm{{\mathcal {A}}}\,}}(T^*)}(a,b,c)) = \newmoon$. By definition, $AB|C \in {\mathfrak {S}}({{\,\mathrm{{\mathcal {A}}}\,}}(T^*),{{\widehat{t}}},\sigma )$. Lemma 28 implies that *ab*|*c* is also displayed by every tree $(T,t,\sigma )\in {\mathfrak {T}}$ and $t({{\,\mathrm{lca}\,}}_{T}(a,b,c))=\newmoon$. Therefore, we have ${\mathfrak {S}}({{\,\mathrm{{\mathcal {A}}}\,}}(T^*),{{\widehat{t}}},\sigma ) \subseteq {\mathfrak {S}}(T,t,\sigma )$ for all $(T,t,\sigma )\in {\mathfrak {T}}$. Now suppose that Condition (1) holds. Then, by Thm. 13, ${\mathfrak {S}}({{\,\mathrm{{\mathcal {A}}}\,}}(T^*),{{\widehat{t}}},\sigma )$ is incompatible. Thus, ${\mathfrak {S}}(T,t,\sigma )$ must be incompatible as well for every tree $(T,t,\sigma )\in {\mathfrak {T}}$. Together with Thm. 13, this implies Condition (2).

Using the arguments in the proof of Thm. 12 and Thm. 13 we find that Condition (1) can be verified in polynomial time by checking whether ${\mathfrak {S}}({{\,\mathrm{{\mathcal {A}}}\,}}(T^*),{{\widehat{t}}},\sigma )$ is incompatible. $\square$

It is possible, therefore to check in polynomial time whether the cograph ${\mathbb {N}}{\mathbb {H}}(\vec {G},\sigma )$ is a biologically feasible orthology relation for $(\vec {G},\sigma )$ or whether ${\mathbb {N}}{\mathbb {H}}(\vec {G},\sigma )$ contains further false-positive edges.

Now consider again a true evolutionary scenario $({\widetilde{T}},{\widetilde{t}},\sigma )$. While ${\widetilde{T}}$ always displays the LRT $(T^*,\sigma )$ of the BMG $\vec {G}({\widetilde{T}},\sigma )$, it does not necessarily display the augmented tree ${{\,\mathrm{{\mathcal {A}}}\,}}(T^*)$. As an example consider the scenario in Fig. 7. Augmenting the only multifurcation in this case further resolves the root of $T^*$ and thus yields a tree that is not displayed by ${\widetilde{T}}$. It is interesting to ask, therefore, whether there are situations in which ${\widetilde{T}}$ does display ${{\,\mathrm{{\mathcal {A}}}\,}}(T^*)$.

#### Lemma 30

Let $(T,t,\sigma )$ be an event-labeled tree explaining the BMG $(\vec {G},\sigma )$, and let $(T^*,\sigma )$ be the least resolved tree of $(\vec {G},\sigma )$. If $(\Theta (T,t),\sigma ) = {\mathbb {N}}{\mathbb {H}}(\vec {G},\sigma )$, then ${{\,\mathrm{{\mathcal {A}}}\,}}(T^*)$ is displayed by *T*.

#### Proof

Let ${\mathfrak {T}}$ be the set of trees corresponding to $(\vec {G},\sigma )$ as given by Eq. (4). First note that $(T,t,\sigma )\in {\mathfrak {T}}$ and that $(T^*,\sigma )$ is displayed by $(T,\sigma )$ (cf. Geiß et al. 2019, Thm. 8). Now consider the set $r({{\,\mathrm{{\mathcal {A}}}\,}}(T^*))$ of all triples displayed by ${{\,\mathrm{{\mathcal {A}}}\,}}(T^*)$. For any triple $ab|c\in r({{\,\mathrm{{\mathcal {A}}}\,}}(T^*))$, there are exactly two cases: (a) ${{\widehat{t}}}(u)=\newmoon$ and (b) ${{\widehat{t}}}(u)=\square$, where $u:={{\,\mathrm{lca}\,}}_{{{\,\mathrm{{\mathcal {A}}}\,}}(T^*)}(a,b,c)$.

In Case (a), Lemma 28 together with $(T,t,\sigma )\in {\mathfrak {T}}$ immediately implies that *ab*|*c* is also displayed by *T*.

In Case (b), we have ${{\widehat{t}}}(u)=\square$. Consider the child $v\in \mathsf {child}_{{{\,\mathrm{{\mathcal {A}}}\,}}(T^*)}(u)$ with $a,b\prec _{{{\,\mathrm{{\mathcal {A}}}\,}}(T^*)}v$. Assume, for contradiction, that *v* is not a vertex in $T^*$, i.e., it was newly created by augmenting a vertex $u'$. We have $u'=u$ by Lemma 22 since $u'$ cannot be (non-trivially) augmented any further. Since ${{\,\mathrm{{\mathcal {A}}}\,}}(T^*)$ does not depend on the order of augmentation steps, we may assume w.l.o.g. that *v* was created in the first augmentation step; resulting in the augmented tree $T_{u}$. Def. 18 implies that ${\mathfrak {C}}_{T}(u)$ is disconnected. Together with Lemma 21, this implies $\sigma (L(T_u(w_1)))\cap \sigma (L(T_u(w_2)))=\emptyset$ for any two distinct vertices $w_1,w_2\in \mathsf {child}_{T_u}(u)$. This must still hold for $({{\,\mathrm{{\mathcal {A}}}\,}}(T^*),\sigma )$ since the edges *uw*, where $w\in \mathsf {child}_{T_u}(u)$ correspond to the vertices that have been newly introduced in the first augmentation step, do not change in any subsequent augmentation due to Lemma 22. The definition of the extremal event labeling now implies ${{\widehat{t}}}(u)=\newmoon$; a contradiction. Therefore, we conclude that *v* is a vertex in $T^*$, and in particular, $a,b\in L(T^*(v))$ and $c\notin L(T^*(v))$, which in turn implies that *ab*|*c* is displayed by $T^*$. From $T^*\le T$ we finally conclude that *T* also displays *ab*|*c*. Denoting by *r*(*T*) the set of all triples displayed by *T* we therefore have $r({{\,\mathrm{{\mathcal {A}}}\,}}(T^*))\subseteq r(T)$. Finally, we apply Thm. 1 of Bryant and Steel (1995) to conclude that ${{\,\mathrm{{\mathcal {A}}}\,}}(T^*)$ is displayed by *T*. $\square$

## Quartets, hourglasses, and the structure of reciprocal best match graphs

### Hourglass-free BMGs

#### Definition 20

A BMG $(\vec {G},\sigma )$ is *hourglass-free* if it does not contain an hourglass as an induced subgraph.

In particular, an hourglass-free BMG also does not contain an hourglass chain. We will need the following technical result

#### Lemma 31

Let $(\vec {G},\sigma )$ be a BMG explained by $(T,\sigma )$. Then $(\vec {G},\sigma )$ has an hourglass  as an induced subgraph if and only if there is a vertex $u\in V^0(T)$ with distinct children $v_1$, $v_2$, and $v_3$ and two distinct colors *r* and *s* satisfying

1. $$r\in \sigma (L(T(v_1)))$$, $$r,s\in \sigma (L(T(v_2)))$$, and $$s\in \sigma (L(T(v_3)))$$, and
2. $$s\notin \sigma (L(T(v_1)))$$, and $$r\notin \sigma (L(T(v_3)))$$.

#### Proof

First assume that $(\vec {G},\sigma )$ contains the hourglass  as an induced subgraph. Then by Lemma 14, $(T,\sigma )$ contains a vertex $u\in V^0(T)$ with three distinct children $v_1$, $v_2$, and $v_3$ such that $x\preceq _T v_1$, ${{\,\mathrm{lca}\,}}_T(x',y')\preceq _T v_2$ and $y\preceq _T v_3$. Putting $r:=\sigma (x)=\sigma (x')$ and $s:=\sigma (y)=\sigma (y')$ immediately implies Condition (1). Now, assume for contradiction that Condition (2) is violated and thus $s\in \sigma (L(T(v_1)))$ or $r\in \sigma (L(T(v_3)))$. If $s \in \sigma (L(T(v_1)))$, then there is a leaf $y''\prec _T v_1$ with $\sigma (y'') =s$. In this case, however, ${{\,\mathrm{lca}\,}}(x,y'')\preceq _T v_1 \prec _T u = {{\,\mathrm{lca}\,}}_T(x,y')$ implies that $(x,y')$ cannot be an arc in $(\vec {G},\sigma )$; a contradiction to  being an hourglass. By similar arguments, $r\in \sigma (L(T(v_3)))$ is not possible. Therefore, Condition (2) must be satisfied.

Now assume that there is a vertex $u\in V^0(T)$ with pairwise distinct children $v_1$, $v_2$, and $v_3$ and two distinct colors *r* and *s* satisfying Conditions (1) and (2). It is now straightforward to see that $(\vec {G},\sigma )$ contains an hourglass: Condition (1) immediately implies the existence of vertices $x\in L[r]\cap L(T(v_1))$ and $y\in L[s]\cap L(T(v_3))$. Moreover, $r,s\in \sigma (L(T(v_2)))$ together with Lemma 3 imply that there is an edge $x'y'$ in $(\vec {G},\sigma )$ with $x'\in L[r]\cap L(T(v_2))$ and $y'\in L[s]\cap L(T(v_2))$. Clearly, the vertices in $\{x,x',y,y'\}$ are pairwise distinct. By Condition (2) and the location of the four leaves, we obtain the arcs $(x,y')$, (*x*, *y*), $(y,x')$, and (*y*, *x*), and thus, in particular the edge *xy*. Since $T(v_2)$ contains both colors *r* and *s*, we can furthermore conclude that $(x',y)$ and $(y',x)$ are not arcs in $(\vec {G},\sigma )$. In summary, the subgraph of $(\vec {G},\sigma )$ induced by the set $\{x,x',y,y'\}$ is an hourglass . $\square$

In the following a tree $(T,\sigma )$ is called *refinable* if there is a proper refinement $(T',\sigma )$ of $(T,\sigma )$, i.e., $T\le T'$ and $T\ne T'$, such that $\vec {G}(T',\sigma )=\vec {G}(T,\sigma )$. Otherwise, $(T,\sigma )$ is *non-refinable*. An inner vertex of a tree is *non-refinable* if it cannot be refined without changing the best match graph induced by the tree.

Clearly, for every BMG $(\vec {G},\sigma )$, there is a tree that has the maximum number of vertices among all trees that explain $(\vec {G},\sigma )$ and thus, a tree that cannot be further resolved. Hence, every BMG can be explained by a non-refinable tree. We will need the following useful property of non-refinable vertices:

#### Lemma 32

Let $(\vec {G},\sigma )$ be a BMG explained by a tree $(T,\sigma )$, and let $u\in V^0(T)$ be a non-refinable vertex of $(T,\sigma )$. Then, for any proper subset $C\subsetneq \mathsf {child}_{T}(u)$ with $|C|\ge 2$, there are two distinct vertices $v,v'\in C$, a vertex $v''\in \mathsf {child}_{T}(u){\setminus } C$, and two vertices $a\preceq _{T} v$ and $b\preceq _{T} v'$ such that $(a,b)\in E(\vec {G})$ and $\sigma (b)\in \sigma (L(T(v'')))$.

#### Proof

First note that the statement is trivially true if *u* is binary, since then there is no proper subset $C\subsetneq \mathsf {child}_{T}(u)$ such that $|C|\ge 2$. Thus, assume $|\mathsf {child}_{T}(u)|\ge 3$ in the following.

We refine $(T,\sigma )$ at vertex *u* as follows: Take an arbitrary subset $C\subsetneq \mathsf {child}_{T}(u)$ such that $|C|\ge 2$ (which exists since $|\mathsf {child}_{T}(u)|\ge 3$) and place all vertices in *C* as the children of a new vertex *w*, and connect *w* as a child of *u*. Since *u* is a non-refinable vertex of $(T,\sigma )$, this refinement leads to a tree $(T',\sigma )$ that does not explain $(\vec {G},\sigma )$, and therefore, the inner edge *uw* must be non-redundant w.r.t. $\vec {G}(T',\sigma )$. By Lemma 7, there must be an arc (*a*, *b*) in $\vec {G}(T',\sigma )$ such that ${{\,\mathrm{lca}\,}}_{T'}(a,b)=w$ and $\sigma (b)\in \sigma (L(T'(u)){\setminus } L(T'(w)))$. In particular, ${{\,\mathrm{lca}\,}}_{T'}(a,b)=w$ implies that $a\preceq _{T} v$ and $b\preceq _{T} v'$ for two distinct vertices $v,v'\in \mathsf {child}_{T'}(w)=C$. Note that $(T,\sigma )$ can be obtained from $(T',\sigma )$ by contraction of the edge *uw*. Hence, we can apply Lemma 8 to conclude that $\vec {G}(T',\sigma )\subseteq (\vec {G},\sigma )$. Therefore, $(a,b)\in E(\vec {G})$. Taking the latter arguments together, for any subset $C\subsetneq \mathsf {child}_{T}(u)$ with $|C|\ge 2$, there are vertices $a\preceq _{T} v$ and $b\preceq _{T} v'$ with distinct $v,v'\in C$ such that $(a,b)\in E(\vec {G})$ and $\sigma (b)\in \sigma (L(T(v'')))$ for some $v''\in \mathsf {child}_{T}(u){\setminus } C$. $\square$

#### Proposition 8

A BMG $(\vec {G},\sigma )$ can be explained by a binary tree if and only if it is hourglass-free.

#### Proof

If the BMG $(\vec {G},\sigma )$ can be explained by a binary tree, it must be hourglass-free as a consequence of Lemma 14. To prove the converse, we assume, for contradiction, that $(\vec {G},\sigma )$ is hourglass-free and cannot be explained by any binary tree. Then there is a non-refinable non-binary tree $(T,\sigma )$ that explains $(\vec {G},\sigma )$. By construction, furthermore, *T* contains a non-binary vertex $u\in V^0(T)$, which by assumption is non-refinable.

The key device for our proof are pairs $({\mathscr {M}},{\mathscr {N}})$ where ${\mathscr {M}}:=\{v_1,\dots ,v_k\}$ is an ordered set of $k\ge 2$ pairwise distinct children of *u* and ${\mathscr {N}}:=\{c_1,\dots ,c_{k-1}\}$ is an ordered set of $k-1$ pairwise distinct colors. We call $({\mathscr {M}},{\mathscr {N}})$ an *hourglass-free pair (hf-pair) of order* *k* for *u* if the following conditions are satisfied:

(i) For all $c_i\in {\mathscr {N}}$ we have $c_i\in \sigma (L(T(v_j)))$, $i\le j\le k-1$,
(ii) For all $c_i\in {\mathscr {N}}$ we have $c_i\notin \sigma (L(T(v_j)))$, $1\le j<i$, and
(iii) $${\mathscr {N}} \subseteq \sigma (L(T(v_k)))$$.

If $({\mathscr {M}},{\mathscr {N}})$ is an hf-pair of order *k*, then Condition (i) implies by construction that ${\mathscr {N}} \subseteq \sigma (L(T(v_{k-1})))$. Therefore, $({\mathscr {M}}'=(v_1,\dots ,v_{k},v_{k-1}),{\mathscr {N}})$ is also an hf-pair where ${\mathscr {M}}'$ is obtained from ${\mathscr {M}}$ by exchanging the positions of its last two elements. Hf-pairs and the following arguments are illustrated in Fig. 15. In order to obtain the desired contradiction, we show by induction that the children of the non-binary, non-refinable vertex *u* harbor hf-pairs of arbitrary large order *k*.

**Base case.** There is an hf-pair $({\mathscr {M}},{\mathscr {N}})$ of order 2 for *u*.

#### Proof of Claim

Consider an arbitrary subset $\{v,v'\}\subsetneq \mathsf {child}_{T}(u)$ consisting of two distinct children *v* and $v'$ of the non-binary vertex *u*. By Lemma 32 and since *u* is non-refinable, there are vertices $a\preceq _{T} v$ and $b\preceq _{T} v'$ such that w.l.o.g. $(a,b)\in E(\vec {G})$ and $\sigma (b)\in \sigma (L(T(v'')))$ for some $v''\in \mathsf {child}_{T}(u){\setminus } \{v,v'\}$. The latter implies that there is a vertex $b'\preceq _{T}v''$ of color $\sigma (b)$. Clearly, *b* and $b'$ are distinct and the color $\sigma (b)$ is also present in the subtree $T(v')$. Thus we can set ${\mathscr {M}}:=(v_1:=v', v_2:=v'')$ and ${\mathscr {N}}:=(c_1:=\sigma (b))$. It is an easy task to verify that $({\mathscr {M}},{\mathscr {N}})$ satisfies Conditions (i)–(iii). $\square$

**Induction step.** The existence of an hf-pair of order *k* implies the existence of an hf-pair of order $k+1$ for *u*.

#### Proof of Claim

Let $({\mathscr {M}}= (v_1,\dots ,v_k),{\mathscr {N}}= (c_1,\dots ,c_{k-1}))$ be an hf-pair, and consider the set $\{v_{k-1},v_k\}\subsetneq \mathsf {child}_{T}(u)$. By Lemma 32 and since *u* is non-refinable, there are again vertices $a\preceq _{T} v$ and $b\preceq _{T} v'$ for distinct $v,v'\in \{v_{k-1},v_k\}$ such that $(a,b)\in E(\vec {G})$ and $\sigma (b)\in \sigma (L(T(v'')))$ for some $v''\in \mathsf {child}_{T}(u){\setminus } \{v_{k-1},v_k\}$. We can assume w.l.o.g. that $a\preceq _{T} v=v_{k-1}$ and $b\preceq _{T} v'=v_k$ since otherwise we can simply swap $v_{k-1}$ and $v_{k}$ in the ordered set ${\mathscr {M}}$ as argued above. Since (*a*, *b*) is an arc in $(\vec {G},\sigma )$ and ${{\,\mathrm{lca}\,}}_{T}(a,b)=u$, the color $\sigma (b)$ cannot be present in the subtree $T(v_{k-1})$. Since ${\mathscr {N}}\subseteq \sigma (L(T(v_{k-1})))$ and $\sigma (b)\notin \sigma (L(T(v_{k-1})))$, we conclude that $\sigma (b)\notin {\mathscr {N}}$.

We continue to show that $v''$ is distinct from all elements in ${\mathscr {M}}$. Clearly, in the case $k=2$, $v''$ is distinct from all elements in ${\mathscr {M}} = \{v_1,v_2\}=\{v,v'\}$ by construction. Now let $k>2$ and assume, for contradiction, that there is a vertex $v_j\in \{v_1,\dots ,v_{k-2}\}$ such that $\sigma (b)\in \sigma (L(T(v_j)))$. In this case, $j<k-1$ and Condition (ii) imply that $c_{k-1}\notin \sigma (L(T(v_j)))$. In addition, we have $c_{k-1}\in \sigma (L(T(v_{k-1})))$ and $c_{k-1}\in \sigma (L(T(v_{k})))$ by Conditions (i) and (iii), respectively. Recall that $v'=v_k$. In summary, we obtain three distinct vertices $v_j,v_k,v_{k-1}$ and two distinct colors $\sigma (b)$ and $c_{k-1}$ satisfying Conditions (1) and (2) in Lemma 31, which implies that $(\vec {G},\sigma )$ contains an hourglass; a contradiction. Hence, $\sigma (b)\notin \sigma (L(T(v_j)))$ for all $j\in \{1,\dots , k-2\}$. This implies that $v''$ is distinct from $v_1,\dots ,v_{k-2}$. Moreover, by construction, $v''$ is distinct from $v_{k-1}$ and $v_k$. In summary, $v''$ is therefore distinct from all elements in ${\mathscr {M}}$.

Consider now the pair $({\mathscr {M}}' :=(v_1,\dots ,v_k,v_{k+1}:=v''), {\mathscr {N}}':=(c_1,\dots ,c_{k-1},c_k:=\sigma (b)))$. Since $({\mathscr {M}},{\mathscr {N}})$ is an hf-pair, and since, by construction, $c_k=\sigma (b)\notin \sigma (L(T(v_{j})))$ for $1\le j\le k-1$ and $c_k=\sigma (b)\in \sigma (L(T(v_{k})))$, we can immediately conclude that Conditions (i) and (ii) are satisfied for $({\mathscr {M}}',{\mathscr {N}}')$. It remains to show that Condition (iii) is satisfied as well, i.e., $c_i\in \sigma (L(T(v_{k+1})))$ for all $1\le i\le k$. By construction, we have $c_k\in \sigma (L(T(v_{k+1})))$. Now assume that $c_i\notin \sigma (L(T(v_{k+1})))$ for some $1\le i \le k-1$. We have $c_i\in \sigma (L(T(v_{k-1})))$ and $c_i,c_k\in \sigma (L(T(v_{k})))$ by Condition (i), and $c_k\notin \sigma (L(T(v_{k-1})))$ by Condition (ii). Taken together, we obtain three distinct vertices $v_{k-1},v_{k},v_{k+1}$ and two distinct colors $c_i$ and $c_k$ satisfying Conditions (1) and (2) in Lemma 31, which implies that $(\vec {G},\sigma )$ contains an hourglass; a contradiction. Therefore, Condition (iii) must be satisfied as well, and $({\mathscr {M}}',{\mathscr {N}}')$ is an hf-pair of order $k+1$. $\square$

Repeated application of the induction step implies that children of a non-refinable non-binary vertex *u* in a non-refinable tree $(T,\sigma )$ explaining an hourglass-free BMG harbor an hf-pair of arbitrary order. This is of course impossible since *G* is finite, i.e, no such vertex *u* can exist. Therefore, every hourglass-free BMG $(\vec {G},\sigma )$ can be explained by a binary tree. $\square$

Prop. 8 gives rise to a procedure for determining whether a BMG $(\vec {G},\sigma )$ can be explained by a binary tree. We simply need to check whether $(\vec {G},\sigma )$ is hourglass-free, a task that can be done trivially in $O(|E(\vec {G})|^2)$ time by checking, for all pairs of edges *ab* and $a'b'$ (in constant time), whether or not they induce an hourglass  or , respectively. Hence, we obtain

#### Corollary 6

It can be decided in polynomial time whether a BMG $(\vec {G},\sigma )$ can be explained by a binary tree.

It remains open, however, whether such a tree can be constructed efficiently.

Geiß et al. (2020c) found that a certain type of colored 6-cycles is an important characteristic of RBMGs with a “complicated” structure that can only be explained by multifurcating trees. Let us write $\langle x_1 x_2\dots x_k\rangle$ for an induced cycle $C_k$ with edges $x_i x_{i+1}$, $1 \le i \le k-1$, and $x_k x_1$ in the symmetric part *G* of $\vec {G}$. We say that $(\vec {G},\sigma )$ contains a *hexagon* if the corresponding RBMG $(G,\sigma )$ contains an induced $C_6 = \langle x_1 x_2\dots x_6\rangle$ such that any three consecutive vertices of $C_6$ have pairwise distinct colors, i.e., $\sigma (x_i)=\sigma (x_i+3)$, $1\le i\le 3$. Since hexagons contain $P_4$s and, by (Geiß et al. 2020c, Lemma 32), any $P_4$ is either a good or a bad quartet, there are exactly two possible induced subgraphs spanned by a hexagon $C_6 = \langle x_1 x_2\dots x_6\rangle$, which are shown in Fig. 16. A graph $(\vec {G},\sigma )$ is *hexagon-free* if it does not contain a hexagon.

#### Lemma 33

Every hourglass-free BMG $(\vec {G},\sigma )$ is hexagon-free.

#### Proof

By Prop. 8, every hourglass-free BMG $(\vec {G},\sigma )$ can be explained by a binary tree. Lemma 9 in Geiß et al. (2020b) implies that hexagons can only be explained by non-binary trees. Hence, $(\vec {G},\sigma )$ must be hexagon-free. $\square$

Clearly, the converse of Lemma 33 is not always satisfied, since, by Obs. 5, an hourglass is a BMG without hexagons.

A very useful observation in previous work is the fact that every 3-colored vertex induced subgraph of an RBMG $(G,\sigma )$ is again an RBMG (Geiß et al. 2020c, Thm. 7). Furthermore, the connected components $(C,\sigma )$ of every 3-colored vertex induced subgraph of $(G,\sigma )$ belong to precisely one of the three types (Geiß et al. 2020c, Thm. 5):

**Type (A)**: $(C,\sigma )$ contains a $K_3$ on three colors but no induced $P_4$.
**Type (B)**: $(C,\sigma )$ contains an induced $P_4$ on three colors whose endpoints have the same color, but no induced cycle $C_n$ on $n\ge 5$ vertices.
**Type (C)**: $(C,\sigma )$ contains a hexagon.

The graphs for which all such 3-colored connected components are of Type (A) are exactly the RBMGs that are cographs, or co-RBMGs for short (Geiß et al. 2020c, Thm. 8 and Remark 2). Together with Lemma 33, this classification immediately implies

#### Corollary 7

Let $(\vec {G},\sigma )$ be an hourglass-free BMG. Then its symmetric part $(G,\sigma )$ is either a co-RBMG or it contains an induced $P_4$ on three colors whose endpoints have the same color, but no induced cycle $C_n$ on $n\ge 5$ vertices.

Since all *u-fp* edges in an hourglass-free BMG are contained in quartets, we have

#### Corollary 8

Let $(\vec {G},\sigma )$ be an hourglass-free BMG. Then its symmetric part $(G,\sigma )$ is a co-RBMG if and only if there are no *u-fp* edges in $(\vec {G},\sigma )$.

#### Proof

Since $(G,\sigma )$ is a cograph, it contains no induced $P_4$s and thus, $(\vec {G},\sigma )$ contains no good or ugly quartets. By Thm. 11, all hug-edges are determined by hourglass chains and good or ugly quartets. Since none of them is contained in $(\vec {G},\sigma )$, it also does not contain *u-fp* edges. Conversely, suppose that $(\vec {G},\sigma )$ contains no *u-fp* edges. Then, by Thm. 10, $(G,\sigma ) = {\mathbb {N}}{\mathbb {H}}(\vec {G},\sigma )$ is an orthology graph and thus, by Thm. 1, a cograph. $\square$

### *u-fp* edges in hourglass chains

The situation is much more complicated in the presence of hourglasses. We start by providing sufficient conditions for *u-fp* edges that are identified by hourglass chains.

#### Proposition 9

Let  be an hourglass chain in $(\vec {G},\sigma )$, possibly with a left tail *z* or a right tail $z'$. Then, an edge in $\vec {G}$ is *u-fp* if it is contained in the set

$$\begin{aligned} F =&\{x_iy_j\mid 1\le i \le j \le k\} \cup \{zz'\} \cup \{zy_{i}, x_iz', zy'_{i}, x'_{i}z' \mid 1 \le i \le k \}\\&\cup \{ x_{i}x_{j+1} \mid 1\le i< j< k \} \cup \{ y_{i}y_{j+1} \mid 1\le i< j < k \} \\&\cup \{x'_1 y'_i, x'_1 y_i \mid 2 \le i \le k \} \cup \{x_i y'_k, x'_i y'_k \mid 1 \le i \le k-1 \} \\&\cup \{x'_1 z, x'_1 z', y'_k z, y'_k z'\} \end{aligned}$$

#### Proof

Let $(T,\sigma )$ be an arbitrary tree that explains $(\vec {G},\sigma )$. By analogous arguments as in the proof of Lemma 17 and by Lemma 16, there is a vertex $u\in V^0(T)$ with pairwise distinct children $v_0,v_1,\dots ,v_k,v_{k+1}$ such that it holds $x_1\in L(T(v_0))$, $y_k\in L(T(v_{k+1}))$ and, for all $1\le i\le k$, we have $x'_i,y'_i\in L(T(v_i))$. Since $x_{i+1}=y'_i$ and $x'_{i+1}=y_i$ by definition of hourglass chains, it is an easy task to verify that for all edges $e=ab\in F$ the vertices *a* and *b* are located below distinct children of *u* and thus, ${{\,\mathrm{lca}\,}}_T(a,b)=u$ for all such edges. As argued in the proof of Lemma 17, we have $\sigma (L(T(v_0)))\cap \sigma (L(T(v_1)))\ne \emptyset$. The latter arguments together with Lemma 10 imply that every edge in *F* is *u-fp*. $\square$

Figs. 6 and 17 furthermore show that hourglass chains identify false-positive edges that are not associated with quartets in the BMG: The BMG in Fig. 6(A) has the *u-fp* edge *xy*, and the BMG in Fig. 17(B) contains the *u-fp* edges $x_1y_2$, $x_1z'$ and $x'_1z'$. A careful investigation shows that these edges are either not even part of an induced $P_4$ (such as *xy* in Fig. 6 and $x'_1z'$ in Fig. 17), or at least not identifiable as *u-fp* via good, bad or ugly quartets according to Props. 2, 3 and 4, as it is the case for $x_1y_2$ and $x_1z'$ in Fig. 17.

### Four-colored $P_4$s

Geiß et al (2020c, Thm. 8) established that the RBMG $(G,\sigma )$ is a co-RBMG, i.e., a cograph, if and only if every subgraph induced on three colors is a cograph. Therefore, if $(G,\sigma )$ contains an induced 4-colored $P_4$, it also contains an induced 3-colored $P_4$. For hourglass-free BMGs $(\vec {G},\sigma )$ it is clear that a 4-colored $P_4$ always overlaps with a 3-colored $P_4$: In this case ${\mathbb {N}}{\mathbb {H}}(\vec {G},\sigma )$ is obtained by deleting middle edges of good quartets and first edges of ugly quartets. Since ${\mathbb {N}}{\mathbb {H}}(\vec {G},\sigma )$ is a cograph, there is no $P_4$ left, and thus at least one edge of any 4-colored $P_4$ was among the deleted edges. It is natural to ask whether this is true for BMGs in general. Fig. 18 shows that good and ugly quartets are not sufficient on their own: there are 4-colored $P_4$s that do not overlap with the middle edge of a good quartet or the first edge of an ugly quartet. On the other hand, it is clear that at least one of its edges is *u-fp*. This does not imply, however, that the *u-fp* edges in a 4-colored $P_4$ are also edges of 3-colored $P_4$s.

Still, in the context of cograph-editing approaches it is of interest whether the 3-colored $P_4$-s are sufficient. In the following we provide an affirmative answer.

#### Lemma 34

Let $(\vec {G},\sigma )$ be a BMG and ${\mathscr {P}}$ a 4-colored induced $P_4$ in the symmetric part of $(\vec {G},\sigma )$. Then at least one of the edges of ${\mathscr {P}}$ is either the middle edge of some good quartet or the first edge of a bad or ugly quartet in $(\vec {G},\sigma )$.

#### Proof

Let $(T,\sigma )$ be an arbitrary tree that explains $(\vec {G},\sigma )$ and suppose that ${\mathscr {P}}:=\langle abcd \rangle$ is a 4-colored induced $P_4$ in the symmetric part $(G,\sigma )$.

If one of the edges *ab*, *bc*, or *cd* of ${\mathscr {P}}$ is the middle edge of some good quartet or the first edge of some ugly quartet, then we are done. Hence, we assume in the following that this is not the case and show that at least one of the edges of ${\mathscr {P}}$ is the first edge in a bad quartet.

By contraposition of Prop. 5, we have ${\mathcal {S}}^{\cap }(a,b)=\emptyset$, ${\mathcal {S}}^{\cap }(b,c)=\emptyset$ and ${\mathcal {S}}^{\cap }(c,d)=\emptyset$. We set $v:={{\,\mathrm{lca}\,}}_T(b,c)$ with children $v_b,v_c\in \mathsf {child}_{T}(v)$ such that $b\preceq _{T}v_b$ and $c\preceq _{T}v_c$, and $w:={{\,\mathrm{lca}\,}}_T(a,b)$ with children $w_a, w_b\in \mathsf {child}_T(w)$ such that $a\preceq _{T}w_a$ and $b\preceq _{T}w_b$. Note, that $v,v_b,w$, and $w_b$ are pairwise comparable, since they are all ancestors of *b*.

We show that $w=v$. Assume, for contradiction, that (i) $w\prec _T v$ or (ii) $v \prec _T w$. In Case (i), we have $w_a\prec _Tw\preceq _T v_b$ and thus, $\sigma (a)\in \sigma (L(T(v_b)))$. Hence, as ${\mathcal {S}}^{\cap }(b,c)=\emptyset$, it must hold that $\sigma (a)\notin \sigma (L(T(v_c)))$ and $\sigma (c)\notin \sigma (L(T(v_b)))$. Lemma 4 implies $ac\in E(G)$. But then ${\mathscr {P}}$ is not an induced $P_4$; a contradiction. In Case (ii), we have $v_c\preceq _Tv\preceq w_b$ and thus, $\sigma (c)\in \sigma (L(T(w_b)))$. Since ${\mathcal {S}}^{\cap }(a,b)=\emptyset$ we thus have $\sigma (c)\notin \sigma (L(T(w_a)))$ and $\sigma (a)\notin \sigma (L(T(w_b)))$. By Lemma 4, $ac\in E(G)$; again a contradiction. Thus $w=v$. Analogous arguments can be used to establish ${{\,\mathrm{lca}\,}}_T(c,d)=v$. We therefore have $v={{\,\mathrm{lca}\,}}_T(a,b)={{\,\mathrm{lca}\,}}_T(b,c)={{\,\mathrm{lca}\,}}_T(c,d)$. In the following $v_x$ denotes the child of *v* with $x\preceq _Tv_x$ for $x\in \{a,b,c,d\}$. Note, $v_a\ne v_b$, $v_b\ne v_c$ and $v_c\ne v_d$.

We next show that $v_a$, $v_b$, $v_c$, and $v_d$ are pairwise distinct. Fist, assume for contradiction that $v_a=v_c$. Together with ${\mathcal {S}}^{\cap }(c,d)=\emptyset$, this assumption implies that $\sigma (a)\notin \sigma (L(T(v_d)))$ and $\sigma (d)\notin \sigma (L(T(v_c)))$. By Lemma 4, $ad\in E(G)$, contradicting the assumption that ${\mathscr {P}}$ is an induced $P_4$. Hence, $v_a\ne v_c$. By symmetry of ${\mathscr {P}}$, we can use similar arguments to conclude that $v_b\ne v_d$. Finally, assume for contradiction that $v_a= v_d$. Then, $\sigma (d)\in \sigma (L(T(v_a)))$. Hence, ${\mathcal {S}}^{\cap }(a,b)=\emptyset$ implies that $\sigma (d)\notin \sigma (L(T(v_b)))$ and $\sigma (b)\notin \sigma (L(T(v_d)))$. Again Lemma 4 implies $bd\in E(G)$; a contradiction. In summary, $v_a$, $v_b$, $v_c$, and $v_d$ must be pairwise distinct.

We claim $\sigma (c)\in \sigma (L(T(v_a)))$. Since $ad\notin E(G)$ and ${{\,\mathrm{lca}\,}}_{T}(a,d)=v$, Lemma 4 implies that $\sigma (a)\in \sigma (L(T(v_d)))$ or $\sigma (d)\in \sigma (L(T(v_a)))$. By symmetry of ${\mathscr {P}}$, we can w.l.o.g. assume that $\sigma (a)\in \sigma (L(T(v_d)))$ and thus, there is a vertex $a_d\in L(T(v_d))$ with $\sigma (a_d)=\sigma (a)$. In this case, ${\mathcal {S}}^{\cap }(c,d)=\emptyset$ implies that $\sigma (a)\notin \sigma (L(T(v_c)))$. This together with $ac\notin E(G)$ and Lemma 4 implies that $\sigma (c)\in \sigma (L(T(v_a)))$.

We claim $\sigma (d)\in \sigma (L(T(v_a)))$. We assume for contradiction that this is not the case and show that this implies the existence of an ugly quartet $\langle cdc'a'\rangle$ containing *cd* as its first edge, which leads to a contradiction to our initial assumption that none of the edges in ${\mathscr {P}}$ is the first, resp., middle edge of an ugly, resp., good quartet. To see this, note that $\sigma (a),\sigma (c)\in \sigma (L(T(v_a)))$ and Lemma 3 imply that there is an edge $a'c'$ for two vertices $a',c'\prec _T v_a$ with $\sigma (a')=\sigma (a)$ and $\sigma (c')=\sigma (c)$. Since $\sigma (a)=\sigma (a')$ and ${{\,\mathrm{lca}\,}}_T(a',c')\preceq _Tv_a\prec _T v={{\,\mathrm{lca}\,}}_T(a',c)$, we have $a'c\notin E(G)$. Since $\sigma (a_d)=\sigma (a')$ and ${{\,\mathrm{lca}\,}}_T(a_d,d)\preceq _Tv_d\prec _T v={{\,\mathrm{lca}\,}}_T(a',d)$, we have $a'd\notin E(G)$. Now, ${\mathcal {S}}^{\cap }(c,d)$ implies that $\sigma (c)\notin \sigma (L(T(v_d)))$. This and $\sigma (d)\notin \sigma (L(T(v_a)))$ together with Lemma 4 implies that there is an edge $c'd\in E(G)$. Thus, we obtain the ugly quartet $\langle cdc'a'\rangle$ and hence, the desired contradiction. Therefore, $\sigma (d)\in \sigma (L(T(v_a)))$. Because of ${\mathcal {S}}^{\cap }(a,b)=\emptyset$ we also have $\sigma (d)\notin \sigma (L(T(v_b)))$.

Since $\sigma (d)\in \sigma (L(T(v_a)))$, there is a vertex $d_a\preceq v_a$ with $\sigma (d_a)=\sigma (d)$. Moreover, $\sigma (b)\notin \sigma (L(T(v_a))$ and $\sigma (d)\notin \sigma (L(T(v_b)))$ together with Lemma 4 implies that $bd_a\in E(G)$. Furthermore, $\sigma (c)\in \sigma (L(T(v_a)))$ and Lemma 4 imply that $cd_a\notin E(G)$. Now, ${\mathcal {S}}^{\cap }(c,d)=\emptyset$ implies $\sigma (d)\notin \sigma (L(T(v_c)))$ and therefore, ${{\,\mathrm{lca}\,}}_T(c,d_a)=v\preceq {{\,\mathrm{lca}\,}}_T(c,d')$ for all $d'\in L[\sigma (d)]$. Hence, $(c,d_a)\in E(\vec {G})$.

In summary, $\langle dcbd_a \rangle$ is an induced $P_4$ in *G*. By (Geiß et al. 2020c, Lemma 32), every such induced $P_4$ forms either a good, bad, or ugly quartet in $(\vec {G},\sigma )$ and, since $(c,d_a)\in E(\vec {G})$, we can conclude that $\langle dcbd_a \rangle$ is a bad quartet with first edge *cd*, which completes the proof. $\square$

#### Corollary 9

(Geiß et al. 2020c, Thm. 8) Let $(G,\sigma )$ be an RBMG. Then, $(G,\sigma )$ is a cograph if and only if all subgraphs induced by three colors are cographs.

#### Proof

If $(G,\sigma )$ is a cograph, then all its induced subgraphs are also cographs Corneil et al. (1981). Conversely, if $(G,\sigma )$ is not a cograph, then it contains at least one induced $P_4$. By Lemma 34, $(G,\sigma )$ cannot contain only 4-colored $P_4$s and therefore the restriction to at least one combination of three colors contains a $P_4$ and is thus not a cograph. $\square$

## Footnotes

1 A good advice in the time of COVID-19
